# Insights into functional and evolutionary analysis of carbaryl metabolic pathway from *Pseudomonas* sp. strain C5pp

**DOI:** 10.1038/srep38430

**Published:** 2016-12-07

**Authors:** Vikas D. Trivedi, Pramod Kumar Jangir, Rakesh Sharma, Prashant S. Phale

**Affiliations:** 1 Department of Biosciences and Bioengineering, Indian Institute of Technology Bombay, Powai, Mumbai 400076, India; 2 Microbial Biotechnology and Genomics Unit, CSIR-Institute of Genomics and Integrative Biology, Mathura Road, New Delhi 110020, India

## Abstract

Carbaryl (1-naphthyl *N*-methylcarbamate) is a most widely used carbamate pesticide in the agriculture field. Soil isolate, *Pseudomonas* sp. strain C5pp mineralizes carbaryl *via* 1-naphthol, salicylate and gentisate, however the genetic organization and evolutionary events of acquisition and assembly of pathway have not yet been studied. The draft genome analysis of strain C5pp reveals that the carbaryl catabolic genes are organized into three putative operons, ‘upper’, ‘middle’ and ‘lower’. The sequence and functional analysis led to identification of new genes encoding: i) hitherto unidentified 1-naphthol 2-hydroxylase, sharing a common ancestry with 2,4-dichlorophenol monooxygenase; ii) carbaryl hydrolase, a member of a new family of esterase; and iii) 1,2-dihydroxy naphthalene dioxygenase, uncharacterized type-II extradiol dioxygenase. The ‘upper’ pathway genes were present as a part of a integron while the ‘middle’ and ‘lower’ pathway genes were present as two distinct class-I composite transposons. These findings suggest the role of horizontal gene transfer event(s) in the acquisition and evolution of the carbaryl degradation pathway in strain C5pp. The study presents an example of assembly of degradation pathway for carbaryl.

The advent of industrialization has led to release of anthropogenic chemicals into the environment imposing a selective pressure on microbes. This pressure is countered by displaying the high propensity for rapid evolution of novel metabolic pathways as well as its spread through facile horizontal transfer of catabolic genes within the microbial population^1^,^2^,^3^,^4^,^5^,^6^,^7^. Carbaryl (1-naphthyl *N*-methylcarbamate), a carbamate class of compound, is the third most widely used broad-spectrum insecticide in the agriculture field since 1960. The insecticidal activity is due to the ester bond which inhibits acetylcholine esterase competitively^8^. Microorganisms have been reported to utilize carbaryl^9^,^10^,^11^,^12^,^13^,^14^,^15^,^16^, and the complete pathway has been demonstrated at the functional level in soil isolates, *Pseudomonas* sp. strains C4, C5 and C6^15^. Carbaryl is metabolized to the central carbon cycle intermediates *via* 1-naphthol, 1,2-dihydroxynaphthalene, salicylate and gentisate^15^. Based on the metabolic studies, the pathway has been hypothesized to be organized into ‘upper’ (carbaryl to salicylate), ‘middle’ (salicylate to gentisate) and ‘lower’ (gentisate to TCA cycle intermediate) segments^17^. The detailed genetic organization and evolutionary origin of the carbaryl degradation pathway have not yet been reported. The enzyme, 1-naphthol 2-hydroxylase (1NH) responsible for the conversion of 1-naphthol to 1,2-dihydroxynaphthalene in the upper pathway, has been purified and characterized at kinetic level from various carbaryl degrading *Pseudomonas* sp.^18^,^19^,^20^, however the gene encoding 1NH has not been reported so far.

Microorganisms have been exposed to carbaryl since 1960 s. *Pseudomonas* sp. strain C5pp draft genome analysis provides a unique opportunity to explore the probable ancestral origin of various genes and evolutionary events responsible for the evolution of carbaryl metabolic pathway. In the present study, we report the genetic organization of degradation genes and functional identification of carbaryl hydrolase (CH), 1-naphthol 2-hydroxylase (1NH) and 1,2-dihydroxynaphthalene dioxygenase (12DHNDO) involved in carbaryl metabolism in *Pseudomonas* sp. strain C5pp (formerly known as strain C5). Based on the analysis of Supercontig-A, we hypothesize that the degradation property must have been acquired through horizontal gene transfer (HGT) which has further evolved to degrade carbaryl as the carbon source more efficiently.

## Results and Discussion

### Construction and functional screening of genomic DNA library

The genomic DNA library of *Pseudomonas* sp. strain C5pp was constructed in CopyControl fosmid pCC2FOS with a phage titer of 3 × 10^6^ CFU.ml^−1^. The G1 clone, with the ability to degrade gentisate as the carbon source, was digested with *Bam*HI, partially sequenced and annotated (Table S1).

**Figure 1:**
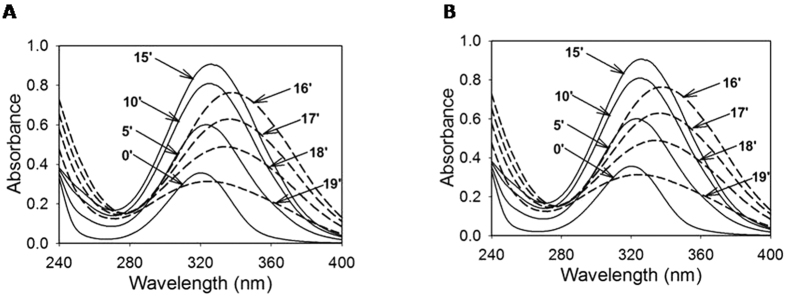
Time-dependent spectral changes observed in the cell-free extracts prepared from cells of (**A**) *E. coli* harboring G1 fosmid DNA (G1 cells) grown on gentisate (0.1%) and (**B**) *Pseudomonas* sp. strain C5pp grown on carbaryl (0.1%) as the carbon source. The enzyme reactions (Phosphate buffer, 50 mM, pH 7.5; gentisate 100 μM and 100 μg of total protein) were scanned from 240–400 nm at every 5 min interval (solid lines). After 20 or 15 min of reaction, glutathione (1 mM) was added in the reaction mixture and spectral changes were recorded at interval of 1 min (dashed lines). The solid lines in both panel represents gentisate dioxygenase (GDO) mediated conversion of gentisate to maleylpyruvate (increase in absorbance at 330 nm, ε_340nm_ = 13,000 M^−1^.cm^−1^). The dashed lines represent the spectral scans observed after addition of glutathione. The observed shift in the absorption peak at 340 nm was due to conversion of maleylpyruvate to fumarylpyruvate by GSH-dependent maleylpyruvate isomerase (MPI). Further, the observed decrease in the absorbance at 340 nm was due to hydrolysis of fumarylpyruvate to fumarate and pyruvate by fumarate-pyruvate hydrolase (FPH). Based on the metabolic studies, the gentisate metabolic pathway in strain C5pp and G1 cells is proposed in panel.

**Figure 2:**
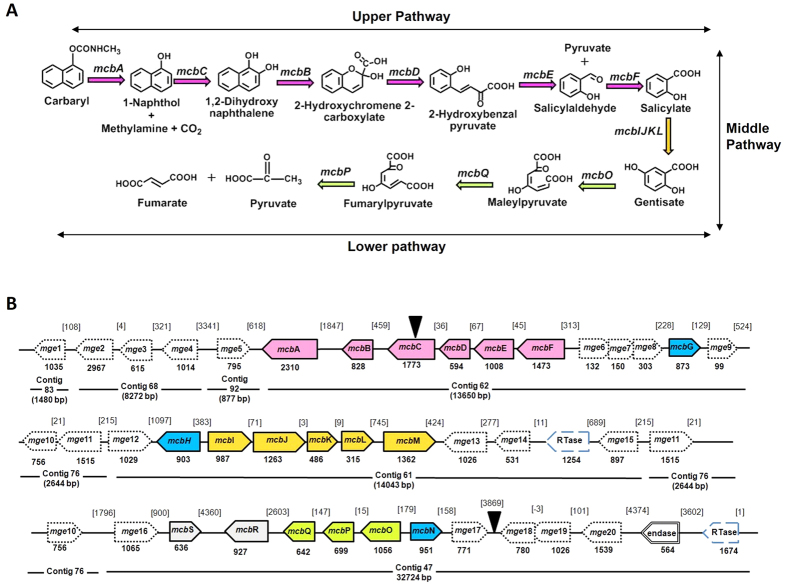
The carbaryl degradation pathway in *Pseudomonas* sp. strain C5pp. The metabolic steps involved in the degradation of carbaryl are depicted in panel (**A**). The arrangement of genes on the Supercontig-A involved in the carbaryl metabolism is represented in panel (**B**). The arrow indicates the direction of gene transcription, numbers indicate the gene length in bp and the number in parenthesis indicate the intergenic distances in bp. *mge* indicates the genes encoding various mobile genetic elements. The details of genes are given in Table 1. The ‘upper’ pathway gens are marked with pink, ‘middle’ pathway with mango yellow and ‘lower’ pathway with green color. The probable functional regulators are marked in blue color. Regulator genes probably not related to the carbaryl metabolism are marked in grey color. ‘RTase’ and ‘endase’ indicates reverse transcriptase and endonuclease, respectively. Supercontig-A consist of contigs from the draft genome sequence of strain C5pp in the order 83-68-92-62-76-61-76-47. The G1 DNA is mapped on Supercontig-A from 17,600 bp to 60,826 bp and is marked by filled arrow head.

*E. coli* EPI300 cells harboring fosmid clone G1-DNA (here onward referred as G1 cells) showed a good growth (O.D_540 nm_ = 0.8 in 24 h) in M9 medium containing gentisate as the sole carbon source. The G1-DNA showed amplification of genes *viz.* salicylaldehyde dehydrogenase (SalDH, 0.5 kb), salicylate 5-hydroxylase (S5H) β-subunit (0.5 kb) and gentisate dioxygenase (GDO, 0.25 kb) by PCR using primers reported earlier^17^, however G1 cells failed to grow on carbaryl or salicylate. The cell-free extract (CFE) prepared from G1 cells grown on M9-glucose showed significantly lower activity of GDO (1 nmole.min^−1^.mg^−1^) as compared to M9-gentisate (276 nmole.min^−1^.mg^−1^) suggesting the inducible nature of GDO. The CFE prepared from G1 (Fig. 1A) as well as strain C5pp (Fig. 1B) cells in the presence of gentisate (100 μM) showed the formation of maleylpyruvate (~47 μM) by the action of GDO. Addition of GSH led to isomerization of maleylpyruvate to fumarylpyruvate by maleylpyruvate isomerase (MPI) and further conversion of fumarylpyruvate to fumarate and pyruvate by fumarylpyruvate hydrolase (FPH, Fig. 2A). The carbon-source dependent enzyme activity and induction studies suggest that the G1-DNA harbors essential ‘lower’ pathway, gentisate metabolic genes along with regulatory elements, thus enabling *E. coli* to use gentisate as the sole source of carbon and energy. Inability of G1 cells to utilize carbaryl is due to lack carbaryl hydrolase (CH) and 1,2-dihydroxy naphantlene dioxygenase (12DHNDO, Fig. 2B). Further cells failed to utilize salicylate, this is probably due to inability of *mcb*H regulatory element to express or function in *E. coli*.

### Genome analysis and validation of genes involved in the carbaryl degradation

The draft genome features and the statistics of *Pseudomonas sp.* strain C5pp (JWLN00000000.1 ref. 21) are summarized in Fig. S1. The sequences obtained from six sub-clones of G1-fosmid DNA (Table S1), when used as a query against C5pp draft genome, retrieved contig 47 (32.72 kb, ‘lower’ pathway), 62 (13.65 kb, ‘upper’ pathway), 61 (14.04 kb, ‘middle’ pathway) and 76 (2.64 kb). Gaps present between these contigs were filled by primer walking and gap filling PCR reactions (Table S2). The assembled contig (76334 bp) here onward is referred as Supercontig-A (KU522233). The genomic region covered in G1-fosmid (~40 kb) is depicted in Fig. 2B and annotation details are summarized in Table S3. Genes involved in the carbaryl degradation and their homology/identity and arrangement are summarized in Table 1 and Fig. 2.

The activities of CH, 1NH, 12DHNDO, SalDH and GDO from strain C5pp has been demonstrated earlier^15^,^22^. NCBI annotation and BLASTp analysis of Supercontig-A identifies following genes with putative functions: i) *mcb*D, 2-hydroxychromene 2-carboxyl isomerase; *mcb*E, *trans*-*o*-hydroxybenzilidinepyruvate aldolase-hydratase; and *mcb*F, SalDH involved in the ‘upper’ pathway; ii) *mcb*IJKL, salicylate 5-hydroxylase involved in the ‘middle’ pathway; and iii) *mcb*O, GDO; *mcb*P, FPH; *mcb*Q, MPI involved in the ‘lower’ pathway of carbaryl metabolism (Fig. 2, Table 1). Beside these genes, it was also found to harbor *mcb*A, *mcb*B and *mcb*C which were analyzed by sequence and function based approaches to ascertain their role in the carbaryl degradation.

### *mcbA* encodes carbaryl hydrolase

**Figure 3:**
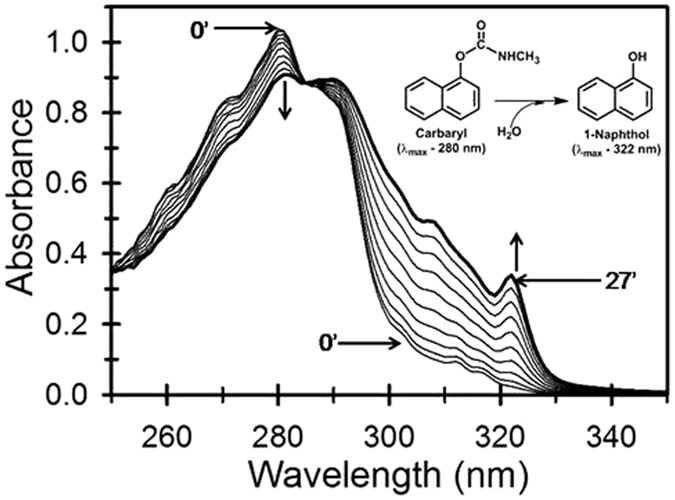
Functional analysis of carbaryl hydrolase (rCH). Time-dependent spectral changes observed in the CFE prepared from *E. coli* BL21(DE3) cells induced with IPTG harboring pET28-CH. The enzyme reaction was scanned from 200-340 nm every 3 min interval for 10 cycles with carbaryl (400 μM) as the substrate. The decrease in the absorbance at 280 nm (down arrow) and increase in the absorbance peak at 322 nm (up arrow) indicated the conversion of carbaryl to 1-naphthol, respectively. The crossover point at 290 nm indicates the isobestic point for the conversion of the carbaryl into 1-naphthol. The lysate from *E. coli* carrying vector alone failed to show any increase in the absorbance at 322 nm over the course of 30 min of incubation indicating absence of CH activity in *E. coli*.

The partially purified CH from strain C5pp was found to be a monomeric protein with mol. wt. of ~80 kDa (Fig. S2). BLASTp analysis of McbA (769 a.a.) annotated as hypothetical protein (Table 1) displayed 24% identity with CehA from *Rhizobium* sp. AC100 23. The predicted molecular mass of McbA (83 kDa, Expasy), was found to be similar to that observed for partially purified CH from strain C5pp (Fig. S2) hence, this hypothetical protein was predicted to be a putative CH. The *mcb*A was cloned into pET-28a(+) and expressed into *E. coli* (Fig. S3A). The CFE (soluble fraction) prepared from IPTG-induced *E. coli* harboring pET28a-CH showed a CH activity of 28 nmole.min^−1^.mg^−1^ as compared to uninduced cells (11 nmole.min^−1^.mg^−1^). Time-dependent spectral scan showed the decrease in the absorbance at 280 nm with concomitant increase in the absorbance at 322 nm due to formation of 1-naphthol (Fig. 3). The observed spectral changes were similar to those reported from wild type strain C5pp^15^. In HPLC analysis, besides carbaryl peak (RT 4.4 min), the reaction mixture gave an additional peak (RT 5.2 min) corresponding to the standard 1-naphthol (Fig. S3B–E). Carbaryl possesses an amide as well as ester linkage, hence hydrolysis can be catalyzed either by esterase or amidase type of enzyme to yield 1-naphthol. The CH from *Rhizobium* sp. strain AC100^23^ and *Arthrobacter* sp. strain RC100^24^ was reported to act as esterase and amidase, respectively. To determine the mode of hydrolysis (either as esterase or amidase type), the activity was tested on 1-naphthylacetate^23^,^24^ or 1-naphthalene acetamide. Recombinant CH from the soluble fraction was found to be active on carbaryl (16 nmole.min^−1^.mg^−1^, 100%), 1-naphthylacetate (5.7 nmole.min^−1^.mg^−1^, 36%) but not on 1-naphthalene acetamide. Similar results were obtained for the partially purified CH from strain C5pp (data not shown), suggesting that the enzyme from strain C5pp is an esterase type CH.

Significant divergence at the functional (amidase or esterase) as well as at the sequence level has been observed for CH. The phylogenetic analysis revealed the clustering of CH from strain C5pp with esterase type of enzymes. The amino acid sequences of CH from strain C5pp and *Rhizobium* sp. AC100 were compared with functionally characterized esterase belonging to fifteen different families. The functionally characterized CHs clustered together and showed unique conserved motif {W-X-S-[AGST]-D-X-H-[ILV]-H-[AIL]-X(3)-[APST]} suggesting a new family of esterases (Fig. S4 and Fig. S5).

### *mcbB* encodes 1,2-dihydroxynaphthalene dioxygenase

**Figure 4:**
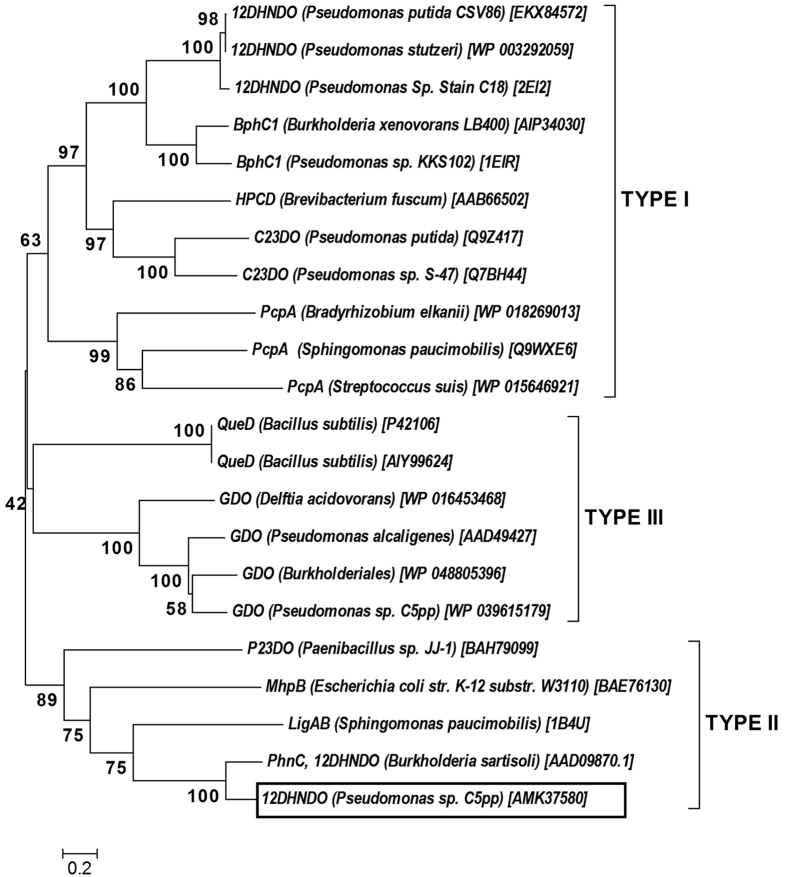
The phylogenetic analysis of 1,2-dihydroxynaphthalene dioxygenase (12DHNDO) from strain C5pp with representative members of three types of EDO . The EDO from strain C5pp clusters with type II EDO which includes functionally characterized PhnC from *Burkholderia*. The numbers in parentheses indicates the protein accession id. Enzyme abbreviations: 12DHNDO, 1,2-dihydroxynaphthalene dioxygenase; BphC1, 2,3-dihydroxybiphenyl 1,2 dioxygenase; HPCD, homoprotocatechuate 2,3-dioxygenase; C23DO, catechol 2,3-dioxygenase; PcpA, 2,6-dichlorohydroquinone 1,2-dioxygenase; QueD, quercetin 2,3-dioxygenase; GDO, gentisate 1,2-dioxygenase; P23DO, protocatechuate 2,3-dioxygenase; MhpB, 2,3-dihydroxyphenylpropionate 1,2-dioxygenase; LigAB, protocatechuate 4,5-dioxygenase.

The BLASTp analysis of McbB (275 a.a., ~30 kDa) retrieved homologs which belong to type-II extradiol dioxygenase (EDO). Phenanthrene and naphthalene degrading *Burkholderia* sp. was reported to harbor *phn*C gene (828 bp, 275 a.a.) which encodes type-II EDO with activity on 1,2-dihydroxynaphthalene^25^. So far, the reported 12DHNDOs are 302 a.a. long, moderately conserved and found to be a member of type-I EDO catalyzing the ring-fission of 1,2-dihydroxynaphthalene to 2-hydroxychromene 2-carboxylate. Thus it was speculated that the protein McbB, encoded by *mcb*B in strain C5pp might catalyze the ring-fission of 1,2-dihydroxynaphthalene. The sequence alignment of the putative McbB with 12DHNDO from *Burkholeria* sp. RP007 showed 68% identity and three conserved motifs- DHY, DHG and GXSH (Fig. S6A) essential for the ring-cleavage activity^26^. The phylogenetic analysis reveals that McbB clustered with type-II EDO of *Burkholderia* and *Ralstonia* (Fig. 4). So far, there are no reports on 12DHNDO from *Pseudomonas* sp. which belongs to type-II EDO.

To validate that *mcb*B encodes 12DHNDO, the gene was cloned into pET-28a(+) and over-expressed in *E. coli* (Fig. S6B). The CFE of IPTG-induced *E. coli* cells harboring pET28a-12DHNDO construct showed 12DHNDO activity (95 nmole.min^−1^.mg^−1^). Partially purified enzyme (Fig. S6B) showed the specific activity of 1.9 μmole.min^−1^.mg^−1^ with 1,2-dihydroxy naphthalene as the substrate. The observed activity for the partially purified r12DHNDO appears to be low as compared to purified 12DHNDO from *Pseudomonas putida* (75 μmole.min^−1^.mg^−1^, type-I EDO^27^) and r12DHNDO form *Burkholderia* sp RP007 (564 μmole.min^−1^.mg^−1^, type-II EDO^25^). Though NCBI server annotated *mcb*B as protocatechuate 3,4-dioxygenase, the partially purified enzyme failed to show activity on protocatechuate, suggesting that *mcb*B codes for 12DHNDO.

### *mcbC* encodes 1-naphthol 2-hydroxylase

**Figure 5:**
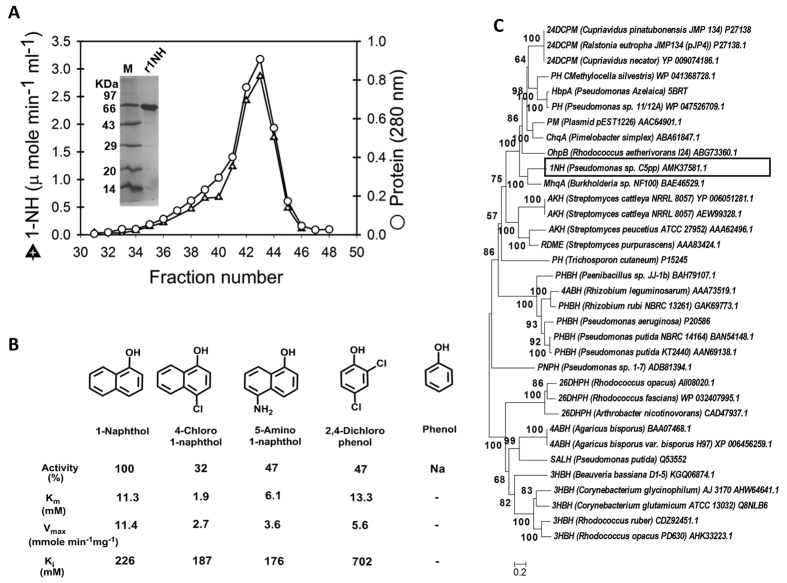
Characterization of 1-naphthol 2-hydroxylase (1NH). (**A**) Gel filtration (Sephacryl S-200 HR) elution profile for recombinant 1NH. The native mol. wt. was determined to be ~145 kDa. Gel filtration column was calibrated with β-amylase (200 kDa), alcohol dehydrogenase (150 kDa), BSA (66 kDa), carbonic anhydrase (29 kDa), and cytochrome c (12.4 kDa). Inset represents the SDS-PAGE profile of purified 1NH (sub unit mol wt. ~66 kDa). The image has been cropped to depict one of the fractions of Sephacryl S-200HR. The complete gel image can be seen in the Supplementary information (Figure S7B). (**B**) Substrate specificity and kinetic constants observed for recombinant 1NH. (**C**) Phylogenetic analysis of 1NH from C5pp with members of PHBH family of monooxygenase. The numbers in parentheses indicates the protein accession id. Enzyme abbreviations are : 24DCPM, 2,4-dichlorophenol monooxygenase; PH, phenol hydroxylase; HbpA, 3-hydroxybiphenyl monooxygenase; PM, phenol monoxygenase; ChqA, chlorobenzoquinol monooxygenase; OhpB 3-(2-hydroxyphenyl) propionic acid monooxygenase; 1NH, 1-naphthol 2-hydroxylase; MhqA, methylbenzoquinol monooxygenase; AKH, aklavinone 11-hydroxylase; RdmE, aklavinone 12-hydroxylase; PHBH, *p*-hydroxybenzoate hydroxylase; 4ABH, 4-aminobenzoate hydroxylase; PnpH, *p*-nitrophenol hydroxylase; 3HBH, 3-hydroxybenzoate hydroxylase; 26DHPH, 2,6-dihydroxyphenol hydroxylase; SALH, salicylate 1-hydroxylase. Na; no activity, -; not determined.

1NH belongs to class-A monooxygenase which consist of single component external flavoproteins requiring NAD(P)H as electron donor^18^,^19^,^20^,^28^. However, the gene encoding 1NH has not been reported till date. N-terminal (MLXNIFLKDE) and partial peptide (FTLLTGIGGEGWIR) sequences of 1-NH purified from strain C5pp showed identity (100%) with amino acid sequence derived from *mcb*C (1773 bp, 590 a.a.) suggesting the gene probably encodes 1NH (Fig. 2). The RAST analysis^29^ predicted McbC of strain C5pp to be a 2,4-dichlorophenol monooxygenase (24DCPM, Table 1). The *mcb*C gene was cloned into pET-28a(+) and over-expressed in *E. coli* (Fig. S7A). The CFE of IPTG-induced *E. coli* cells harboring pET28a-1NH construct showed 1.78 μmole.min^−1^.mg^−1^ 1NH activity. The recombinant 1NH (r1NH) was purified to homogeneity (Fig. S7B, specific activity of 8.2 μmole.min^−1^.mg^−1^; fold purification 3.5 and yield 33%) by Ni-NTA followed by Sephacryl S-200HR gel filtration chromatography (homodimeric protein, native mol. wt. of ~145 kDa and subunit mol. wt. of ~66 kDa; Fig. 5A). The MALDI-TOF/TOF analysis of r1NH and 1NH purified from strain C5pp confirmed that both proteins are identical (Fig. S7C). The observed molecular, kinetic properties and substrate specificities (Fig. 5B) are similar to those reported for wild type 1NH from strain C5pp^20^. Phylogenetic study showed 1NH to be related to monooxygenases acting on phenol and its derivatives (Fig. 5C). r1NH showed activity on 2,4-dichlorophenol (47%) as compared to activity on 1-naphthol (100%), but failed to hydroxylate phenol (Fig. 5B). Results suggests the identification of a new gene *mcb*C encoding 1NH in *Pseudomonas* sp. strain C5pp. The promiscuity of 1NH on unrelated substrate like 2,4-dichlorophenol and the good amino acid sequence identity with 24DCPM (55%) suggests a probable common ancestral origin for enzymes 1NH and 24DCPM. Upon acquiring by strain C5pp, the gene has probably evolved to code for efficient 1NH, thus allowing cells to mineralize carbaryl more rapidly at higher concentrations.

### Phylogenetic analysis of ‘upper’ and ‘middle’ pathway genes

The carbaryl degradation pathway follows metabolic steps similar to naphthalene pathway from 1,2-dihydroxynaphthalene onwards. Based on this observation, we speculate that the genes for carbaryl degradation pathway in strain C5pp must have been acquired and evolved from these more prevalent systems in the presence of positive selection pressure. One of the non-parametric indicators is the systematic phylogenetic studies to aid in understanding the evolutionary relatedness. The analysis of McbD, McbE and McbF indicates that their putative functions as hydroxychromene 2-carboxylate isomerase, *trans*-*o*-hydroxybenzylidenepyruvate hydratase-aldolase, and SalDH, respectively (Fig. S8A–C). Phylogeny analysis of McbIJKL suggested the close relatedness to functionally characterized S5H from *Ralstonia* sp. U2^30^ (Fig. S8D–G).

### Analysis of putative regulators involved in the carbaryl metabolism

Supercontig-A harboring carbaryl degradation genes was found to contain five genes (*mcb*G, *mcb*H, *mcb*N, *mcb*R and *mcb*S) encoding putative regulators. The phylogenetic analysis of McbG, McbH, McbN, McbR and McbS reveals that they are branched into five distinct clusters of LysR/TetR regulators (Fig. S9A).

The amino acid sequence of the McbG (290 a.a., putative regulator of ‘upper’ pathway segment) was similar to that of LysR-type transcriptional regulators. The closest homolog of McbG was found to be LysR-type PhnS (58% identity at a.a. level) regulator involved in phenanthrene metabolism in *Burkholderia* sp. strain RP007^25^. PhnS was found to be a part of polycistronic mRNA with direction of transcription *in-sync* with the downstream genes involved in phenanthrene metabolism. The distance between *mcb*G and its upstream metabolic genes (*mcb*F encoding SalDH) was found to be 1146 bp in contrast to 52 bp observed for *phn*S and its downstream genes. The analysis of the intervening sequence between *mcb*G and their respective upstream genes indicated the presence of fragmented transposases, suggesting that the regulator is acquired by HGT and probably involved in the regulation of ‘upper’ pathway enzymes.

Gene *mcb*H (903 bp, 300 a.a.), a part of the ‘middle’ pathway segment (salicylate degradation) encodes a McbH and showed 67–69% identity to a group of NagR/DntR/NahR type LysR transcriptional regulators from *Pseudomonas* sp. and *Burkholderia* sp. Members of this family are reported to recognize salicylate as the specific effector molecule to induce the degradation of aromatics^31^.

In the vicinity of genes involved in the utilization of gentisate (lower pathway), three genes encoding putative regulator proteins *viz.* McbN (951 bp, 316 a.a., LysR type), McbR (927 bp, 308 a.a., LysR type) and McbS (636 bp, 211 a.a., TetR type) were identified. Among these three regulators, McbN was found to share 79% identity with HbzR (LysR type regulator) from *Pseudomonas alcaligenes* NCIMB 9867 which was reported to be involved in the gentisate metabolism^32^. McbR and S showed relatedness with LysR family transcription regulator from *Burkholderia* sp. lig30 (68%) and TetR family transcription regulator from *Brenneria* sp. EniD312 (64%) with unknown functions.

The ClustalW alignment supports the possibility that McbG, McbH and McbN might recognize same co-inducers as their orthologs (Fig. S9B). Though the co-inducer for PhnS is not known, NahR and HbzR have been shown to recognize salicylate and gentisate, respectively. Overall, the observed gene organization with respective regulator into three distinct ‘upper’, ‘middle’ and ‘lower’ pathways for the carbaryl degradation in strain C5pp corroborates well with biochemical studies reported earlier^17^.

### Evolution of carbaryl degradation pathway

**Figure 6:**
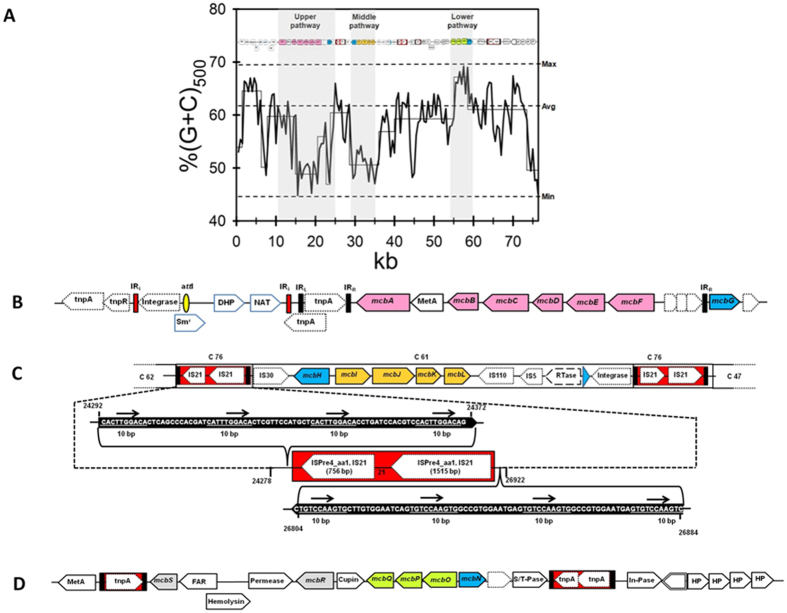
Analysis of carbaryl degradation cluster. (**A**) G+C skew plot of 76333 bp region proposed to be involved in the carbaryl degradation. Thin line indicates G+C content as calculated by G+C viewer (http://tubic.tju.edu.cn/GC-Profile/). Thick line indicates G+C content variation as calculated manually with 500 bp window. Horizontal dashed lines indicate maximum (max), minimum (min) and average (avg) G+C content. The genetic organization of carbaryl degrading genes is depicted at the top. The area shaded in grey represents the G+C content of upper, middle and lower pathway involved in the carbaryl degradation. Genetic organization of genes and mobile genetic elements involved in **(B)** ‘upper’ pathway segment genes, filled yellow oval indicates *att*I, filled red box indicate 25 bp IR_i_ and black rectangular boxes indicate invert repeats IR_L_ and IR_R_, Sm^r^ - streptomycin resistance; DHP, dihydropterate synthase; NAT, *N*-acetyl transferase; IR_t_ site of the integron is missing. (**C**) ‘middle’ pathway segment genes, Red box depicts IS element containing transpsosases flanked by IRs highlighted in black boxes. Blue arrowhead indicates group II intron and (**D**) ‘lower’ pathway segment genes; Red box depicts IS element containing transpsosases flanked by IRs highlighted in black boxes; double lined box represents recombinase; MetA, a conserved protein; FAR, fusaric acid resistance; S/T-Pase, serine/threonine protein phosphatase; In-Pase, inositol phosphatase; HP, hypothetical protein; regulators are marked in blue arrow heads. The orientation of arrow heads indicate the direction of transcription. Genes coding for ‘upper’ pathway enzymes are marked with pink, ‘middle’ pathway with mango yellow and ‘lower’ pathway with green color.

The G+C profile viewer^33^ (http://tubic.tju.edu.cn/GC-Profile/) revealed a skewing in the G+C content of Supercontig-A (Fig. 6A). The G+C content in the region 10629–36324 bp, which harbored ‘upper’ (carbaryl to salicylic acid) and ‘middle’ (salicylate to gentisate) pathway genes, was significantly lower (~54%) than that of strain C5pp (62.65%). The G+C content from 36325–76334 bp, which harbors genes involved in the gentisate metabolism, was observed to be ~60%. The remarkable difference in the G+C content suggests probably a different ancestral origin for genes involved in the ‘upper’ and ‘middle’ pathway. RAST analysis identified 42 transposases in C5pp draft genome, of which 17 (40%) were present in Supercontig-A (Table 1), indicating this region to be hotspot for genome alterations. The upstream region of ‘upper’ pathway genes was found to harbor class-I integron with features like presence of transposase, 25 bp left-end repeat (IR_i_), *att*I site, 5′ and 3′ conserved segment (CS) (Fig. 6B). It also harbors additional 25 bp direct repeat (92% homology to IR_i_), aminoglycoside nucleotidyl transferase (Sm^r^, streptomycin resistance, ANT3class), dihydropteroate synthase (folic acid biosynthesis) and *N*-acetyltransferase (GCN5 family) genes. The invert repeat (IR_t_) at the right-end of integron was absent. Downstream, a transposase (tnpA) of IS6100 family flanked by 25 bp invert repeat was found to be present as seen for Tn6217 followed by genes for the ‘upper’ pathway. Interestingly, the regulator McbG proposed to be involved in the transcription of upper pathway genes was flanked by two truncated transposases (Fig. 6B). The presence of transposon fragments suggest that they have been lost by decay-linked recombination events and might be the remnant of previously functional integrative conjugative element.

Strain C5pp carries a catabolic transposon ‘TnC5ppSal’, which harbors *mcb*IJKL (‘middle’ pathway) and exhibits class-I composite transposon features (IS21 family insertion element repeats, Fig. 6C). The presence of IS elements in inverted orientation probably imparts stability to TnC5ppSal in the genome. The salicylate gene arrangment (*mcb*IJKL) is similar to *Ralstonia* sp. strain U2^34^. However, the region containing transpsoase at either ends showed ~80% identity to *Pseudomonas resinovorans*. This indicates that though there is a synteny between strain C5pp and U2, the transposon for salicylate in strain C5pp has probably a different ancestral origin. Similarly, the ‘lower’ pathway was found to be a part of class-I composite transposon flanked by non-identical IS elements (Fig. 6D). The transposon, is bordered by IS element similar to ISPa1635 and IS481, respectively. Overall, these observations indicate the acquisition of pathway as result of multiple transposition events.

In conclusion, the draft genome sequence of *Pseudomonas* sp. strain C5pp along with concerted approach involving bioinformatics, molecular biology and biochemical analysis led to decipher the carbaryl degradation pathway at genetic level into three distinct putative operons. Genome analysis further reveals that the degradation capability must have been acquired through HGT events. The present study helps to understand the molecular mechanisms involved in the construction and evolution of metabolic pathway for carbaryl. In this perspective, the information becomes particularly important giving avenue to engineer a strain for effective degradation of array of aromatic compounds.

## Materials and Methods

### Bacterial culture and Genomic DNA isolation

Soil isolate *Pseudomonas* sp. strain C5pp (earlier referred as *Pseudomonas* sp. strain C5) utilizes carbaryl as the sole source of carbon and energy was used in this study^15^. Strain C5pp was grown on MSM supplemented with glucose (0.25%) or appropriate aromatics (carbaryl/gentisate, 0.1%) as described previously^15^. *E. coli* EPI300 was grown on Luria-Bertani (LB) broth^35^ or M9-medium^36^ containing glucose or aromatics (carbaryl/salicylate/gentisate, 0.1%) supplemented with leucine (100 μg.ml^−1^), thiamine (10 μg.ml^−1^) and arabinose (0.001%).

The genomic DNA was isolated from strain C5pp grown on carbaryl (0.1%) using UltraClean^®^ Microbial DNA isolation kit (MoBio Laboratories, USA).

### Construction and functional screening of *Pseudomonas* sp. strain C5pp genomic fosmid library

The genomic DNA isolated from strain C5pp was end-repaired using T4 DNA polymerase and T4 polynucleotide kinase (NEB, USA). The fragments of ~40 kb size were eluted from low melting agarose gel using β-agarse (NEB, USA), ligated to the fosmid vector (pCC2FOS) and packaged as per manufacturer’s instructions (Epicentre, USA). The packaged fosmids were transfected into *E. coli* EPI300 and appropriate dilutions were plated onto LB plates containing chloramphenicol (12.5 μg.ml^−1^). The genomic DNA library of *Pseudomonas* sp. strain C5pp was constructed in CopyControl fosmid pCC2FOS with a phage titer of 3 × 10^6 ^CFU.ml^−1^ and a pool of ~9600 colonies was used for screening.

The library pool was plated onto M9 medium supplemented with leucine (100 μg.ml^−1^), thiamine (10 μg.ml^−1^), arabinose (0.001%), aromatic compound (carbaryl/salicylate/gentisate, 0.1%) and chloramphenicol (12.5 μg.ml^−1^). Twenty eight colonies were observed on M9 plates containing gentisate at the end of 8^th^ day indicating the ability to utilize gentisate as the carbon source, whereas plates containing carbaryl or salicylate did not show any colonies.

Five colonies were picked up randomly from M9-gentisate plates and analyzed further. Fosmid DNA was isolated by Fosmid Max DNA purification kit (FMAX 046, USA) and restriction digested with *Bam*HI (~12, 8, 5, 2.8, 2.5 and 1.8 kb) or *Not*I (20, 11, 5, 4.8, 2.7 and 2.3 kb). One of the randomly chosen clone designated as G1 was digested with *Bam*HI and all six fragments were sub-cloned into pUC19 and partially sequenced using universal M13 forward and reverse primers (M13F, 5′-GTTTTCCCAGTCACGAC-3′ and M13R, 5′-CAGGAAACAGCTATGAC-3′, Promega, USA) after cloning it into pUC19 at *Bam*HI site.

### Preparation of cell-free extracts and enzyme assays

Randomly selected single gentisate positive colony of *E. coli* EPI300 was grown for 24 h on M9 medium (150 ml) supplemented with: a) gentisate (0.1%), chloramphenicol (12.5 μg ml^−1^); and b) glucose (0.25%), chloramphenicol (12.5 μg.ml^−1^). Cells were harvested by centrifugation, suspended in ice-cold phosphate buffer (50 mM, pH 7.5) and disrupted by sonication at 4 °C (four cycles with 4 min interval; each cycle: 15 pulses, output 11 W, Ultrasonic processor GE130). Cell homogenate was centrifuged at 40,000×*g* for 30 min. The clear supernatant obtained was referred as CFE and used as the enzyme source. Protein was estimated by Bradford method using bovine serum albumin as the standard^37^. CH, 1NH, and GDO were monitored spectrophotometrically (Perkin Elmer; model Lambda 35) as described^15^,^22^. 12DHNDO was assayed as described^38^ after reactivation. Specific activities are reported as nmole.min^−1^.mg^−1^ of protein. To identify the reaction product of CH, the reaction mixture was subjected to HPLC (Agilent 1200 series) using RP-C18 column (4.6 × 250 mm, particle size 5 μM, Eclipse plus C-18, Agilent) using solvent system methanol:water (60:40 v/v, flow rate 1 ml.min^−1^). The detection was with diode array detector at 280 and 322 nm for carbaryl and 1-naphthol, respectively.

### Identification of genes involved in carbaryl degradation

To predict the genes for carbaryl degradation pathway, the partial sequences obtained from six sub-clones of G1 were blast analysed against the draft genome of strain C5pp, which retrieved contigs 47, 61, 62 and 76. These contigs were further subjected to ORF finder (http://www.ncbi.nlm.nih.gov/projects/gorf/). The predicted genes were analysed further using the function and sequence based approach. Contigs were assembled using gap-filling reactions using primers as mentioned in Table S2.

### Phylogenetic analysis

The phylogenetic trees were inferred for amino acid sequences of similar enzymes/proteins obtained from NCBI BLAST using Neighbor-Joining (NJ) algorithm by using MEGA6 software. The protein sequences were aligned using ClustalW program. The robustness of the tree topology was assessed by bootstrap analysis of 500 replicons using Jones-Taylor-Thornton (JTT) model^39^.

### Cloning and over-expression of carbaryl hydrolase, 1-naphthol 2-hydroxylase and 1,2-dihydroxynaphthalene dioxygenase

The gene predicted for CH, 1NH and 12DHNDO were PCR amplified from genomic DNA of strain C5pp using primers: CHF- 5′-CTAGCTAGCATGGCGGTCACGGCAAATTATTTGC-3′ (under line represents restriction site for *Nhe*I) and CHR- 5′-CCGCTCGAGTCAATGCGCGGCAAGCCGG-3′ (*Xho*I); 1NHF- 5′-CCGGAATTCCATATGCTGAAAAATATTT-3′ (*Eco*RI and *Nde*I) and 1NHR- 5′-CCGGAATTCTTAAAGACAGAGAATTGC-3′ (*Eco*RI); and 12DHNDOF- 5′-CATGCCATGGCTCAGATTGTAGCTGG-3′ (*Nco*I) and 12DHNDOR- 5′CCGCTCGAGGCTAGGCTTCATCTCCATATACCC-3′ (*Xho*I), respectively. The gel purified PCR products (2.31, 1.77, and 0.825 kb for CH, 1NH and 12DHNDO, respectively) were digested with appropriate restriction enzymes and cloned into pET-28a(+) (Novagen). The expressed protein contains His_6_-tag at N-terminus. pET28-CH, pET28–1NH and pET28-12DHNDO were transformed into *E. coli* BL21 (DE3). A single colony obtained was grown on LB medium (500 ml) containing kanamycin (30 μg.ml^−1^) at 37 °C till OD_600 nm_ = 0.8. The culture was chilled on ice for an hour and induced by addition of IPTG (100 μM) for 16 h at 18 °C. Cells were harvested, re-suspended (1:10 g wt./vol.) in potassium-phosphate buffer (50 mM, pH7.5) with glycerol (5%) [Buffer-A] and CFE was prepared. CFE from *E. coli* cells as well as *E. coli* carrying vector alone (absence of gene of interest) were used as control.

### Purification and characterization of recombinant 12DHNDO and 1NH

The recombinant 12DHNDO and 1NH (r1NH) were purified using Ni-NTA matrix (Qiagen, USA, See Supporting Information). r1NH was characterized for substrate specificity and kinetic constants as described earlier (Trivedi *et al*., 2014). r1NH and 1NH purified from strain C5pp were subjected to MALDI-TOF/TOF-MS/MS analysis as described^40^.

## Additional Information

**How to cite this article**: Trivedi, V. D. *et al*. Insights into functional and evolutionary analysis of carbaryl metabolic pathway from *Pseudomonas* sp. strain C5pp. *Sci. Rep.* **6**, 38430; doi: 10.1038/srep38430 (2016).

**Publisher’s note:** Springer Nature remains neutral with regard to jurisdictional claims in published maps and institutional affiliations.

## Supplementary Material

Supplementary Information

## Figures and Tables

**Table 1:** Genes involved in the carbaryl degradation and mobile genetic elements present on Supercontig-A of *Pseudomonas* sp. strain C5pp.

Gene	Predicted function (* indicates activity demonstrated/reported in strain C5pp)	Size (bp)	Protein Homolog	Coverage/Identity	E-value
**A] Enzymes involved in carbaryl metabolism**					
*mcb*A	Carbaryl hydrolase (CH)*	2310	Hypothetical protein HRUBRA_00780 [*Haliea rubra* DSM 19751] (KGE04621)	92/40	0.0
			**Carbaryl hydrolase [*****Rhizobium*** **sp. AC100] (BAB85626.1)**	**46**/**24**	**8e-14**
*mcb*C	1-Napthol 2-hydroxylase (1NH)*	1773	**Methylhydroquinone monooxygenase MhqA [*****Burkholderia*** **sp. NF100] (BAE46529.1)**	**97**/**55**	**0.0**
			2,4-Dichlorophenol 6-monooxygenase [*Burkholderia zhejiangensis*] (KDR27163)	97/55	0.0
			**2,4-Dichlorophenol 6-hydroxylase [*****Ralstonia eutropha*****JMP134 (pJP4)] (P27138.1)**	**97**/**41**	**7e-147**
*mcb*B	1,2-Dihydroxynaphthalene dioxygenase (12DHNDO)*	828	Protocatechuate 3,4-dioxygenase [*Burkholderia* sp. lig30] (WP_038712660.1)	99/73	1e-146
			**Extradiol dioxygenase PhnC [*****Burkholderia*** **sp. strain RP007] (AAD09870.1)**	**99**/**68**	**3e-134**
*mcb*D	2-Hydroxychromene 2-carboxylate isomerase	594	2-Hydroxychromene 2-carboxylate isomerase [*Marinomonas profundimaris*] (WP_024024135.1)	97/55	5e-75
			**2-Hydroxychromene-2-carboxylate isomerase [*****Pseudomonas putida*****] (AAA66358.1)**	**97**/**53**	**9e-75**
*mcb*E	*trans*-o-hydroxybenzylidenepyruvate hydratase-aldolase	1008	*trans*-2′-Carboxybenzalpyruvate hydratase-aldolase [*Burkholderia multivorans*] (AIO75636)	96/79	0.0
			***trans*****-2′-Carboxybenzalpyruvate hydratase-aldolase [*****Pseudomonas putida*****] (AAA66357.1)**	**95**/**78**	**0.0**
*mcb*F	Salicylaldehyde dehydrogenase (SalDH)*	1473	Salicylaldehyde dehydrogenase [*Comamonas testosteroni*] (KGH21325.1)	100/77	0.0
			**Salicylaldehyde dehydrogenase [*****Pseudomonas putida*****G7] (4JZ6)**	**98**/**66**	**0.0**
*mcb*G	LysR	873	LysR-type transcriptional regulator, PhnS [*Burkholderia sartisoli*] (AAD09867.1)	95/58	2e-158
*mcb*H	Transcriptional regulator (NahR)	903	Naphthalene degradation LysR-family transcriptional activator [*Pseudomonas* sp. CF161] (WP_020298853)	100/74	7e-163
			**DntR [*****Burkholderia*** **sp.] (1UTB)**	**100**/**67**	**6e-148**
*mcb*I	Ferredoxin reductase	987	Oxidoreductase component of 2,4-dinitrotoluene dioxygenase DntAa [uncultured bacterium] (BAO02623.1)	100/72	e-46
			**Ferredoxin reductase [*****Ralstonia*** **sp. U2] (AAD12606.1)**	**100**/**75**	**0.0**
*mcb*J	Salicylate 5-hydroxylase large oxygenase component	1263	Salicylate 5-hydroxylase large oxygenase component [*Burkholderia* sp. C3] (ACT53246)	100/90	0.0
			**Salicylate 5-hydroxylase large oxygenase component [*****Ralstonia*** **sp. U2] (AAD12607.1)**	**98**/**91**	**0.0**
*mcb*K	Salicylate 5-hydroxylase small oxygenase component	486	Salicylate 5-hydroxylase small oxygenase component [*Pseudomonas* sp. C6 (2012)]	100/100	0.0
			**Salicylate 5-hydroxylase small oxygenase component [*****Ralstonia*** **sp. U2] (AAD12608.1)**	**100**/**84**	**1e-107**
*mcb*L	Ferredoxin	315	Naphthalene 1,2-dioxygenase [*Polaromonas naphthalenivorans*] (WP_011801868)	99/71	5e-49
			**Naphthalene 1,2-dioxygenase [*****Ralstonia*** **sp. U2] (AAD12609.1)**	**99**/**63**	**1e-55**
*mcb*M	4-Hydroxybenzoate/salicylate transporter	1362	Major facilitator transporter [*Tistrella mobilis*] (WP_014747630.1)	95/56	e-162
			**4-hydroxybenzoate transporter (MFS superfamily) [*****Acinetobacter*** **sp. ADP1] (CAG68551.1)**	**90**/**40**	**4e-104**
*mcb*S	Transcriptional regulator (TetR)	636	LysR family transcriptional regulator [*Burkholderia* sp. lig30] (KDB08170)	99/68	9e-144
*mcb*R	Transcriptional regulator (LysR)	927	TetR family transcriptional regulator [*Brenneria* sp. EniD312] (WP_009111295)	96/64	3e-88
*mcb*Q	Maleyl pyruvate isomerise (MPI)*	642	Maleylacetoacetate isomerase [*Pseudomonas chlororaphis*] (WP_025810436)	99/73	8e-107
			**Maleylpyruvate isomerase [*****Ralstonia*** **sp. U2] (AAD12621.1)**	**98**/**47**	**1e-62**
*mcb*P	Fumaryl pyruvate hydrolase (FPH)*	699	5-Carboxymethyl-2-hydroxymuconate isomerase [*Pseudomonas chlororaphis*] (WP_025810433)	100/81	2e-138
			**Fumaryl pyruvate hydrolase [*****Ralstonia*** **sp. U2] (AAD12620.1)**	**81**/**48**	**5e-60**
*mcb*O	Gentisate dioxygenase (GDO)*	1056	Gentisate 1,2-dioxygenase [*Pseudomonas* sp. GM48] (EJM48134)	99/81	0.0
			**Gentisate 1,2-dioxygenase [*****Ralstonia*** **sp. U2] (AAD12619.1)**	**97**/**36**	**1e-161**
*mcb*N	Transcriptional regulator (LysR)	951	**Putative LysR type transcriptional regulator [*****Pseudomonas alcaligenes*****] (ABD64506)**	**98**/**79**	**7e-167**
**B] Mobile genetic elements present in the Supercontig-A**					
*mge*1	Transposase	1035	IS110 transposase [*Pseudomonas putida*] (AJ288910)	100/93	0
*mge*2	Transposase	2967	Transpsoase [Gammaproteobacteria] (WP_ 001138014)	100/99	0
*mge3*	Transpsoase	615	TnpR [*Pseudomonas aeruginosa*] (ACY75537)	100/98	0
*mge*4	Integrase	1014	Integrase/recombinase [*E. coli* BIDMC 82] (EZQ51649)	100/100	0
*mge*5	Transposase	795	MULTISPECIES: transposase [Gammaproteobacteria] (WP_001375121)	100/100	0
*mge*6	Mobile element protein	132	Transposase [*Pseudomonas* sp. RL] (WP_027591894)	100/84	5e-15
*mge*7	Mobile element protein	150	Transposase [*Pseudomonas* sp. RL] (WP_027591894)	100/90	8e-24
*mge*8	Mobile element protein	303	ISCja1 transposase *orf*B [*Pseudomonas putida* CSV86] (EKX86906)	99/86	4e-53
*mge*9	Transposase	99	ISPssy transposase [*Pseudomonas syringae pv. actinidiae*] (GAO96698)	100/91	1e-11
*mge*10	Mobile element protein	756	ISPre4_aa2; IS21 Family [*Pseudomonas resinovorans*] (BAH10054)	100/93	e-128
*mge*11	Mobile element protein	1515	ISPre4_aa1; IS21 Family [*Pseudomonas resinovorans*] (WP_011077976)	100/90	0
*mge*12	Transposase	1029	ISPa59_aa1; IS30 Family [*Pseudomonas aeruoginosa*] (WP_023101971)	100/90	e-173
*mge*13	Transposase	1026	IS1383_aa1; IS110 Family [*Pseudomonas putida*] (AAC98740)	100/83	e-160
*mge*14	Transposase	531	IS1384_aa1; IS5 Family [*Pseudomonas putida*] (AAC98743)	87/79	7e-67
*mge*15	Integrase	780	ISAeme21; IS481 Family [*Aeromonas media*] (AHX59485)	99/71	2e-128
*mge*16	Transposase	1635	ISPa1635_aa1; IS4 Family [*Pseudomonas aeruginosa*] (AAS59256)	100/99	0
*mge*17	Transposase	771	ISAzsp1; IS3 Family [*Azotobacter* sp.] (CAD42759)	98/86	4e-158
*mge*18	Transposase	780	ISKpn7_aa2; IS21 Family [*Klebsiella pneumoniae*] (WP_0041523940)	100/84	e-125
*mge*19	Transposase	1026	ISKpn7_aa1; IS21 Family [*Klebsiella pneumoniae*] (YP_002286833)	100/75	e-140
*mge*20	Transposase	1539	ISBmu11_aa1; IS3 Family [*Burkholderia multivorans*] (ABX19319)	100/64	0.0

The functionally characterized homologs are shown in bold.
