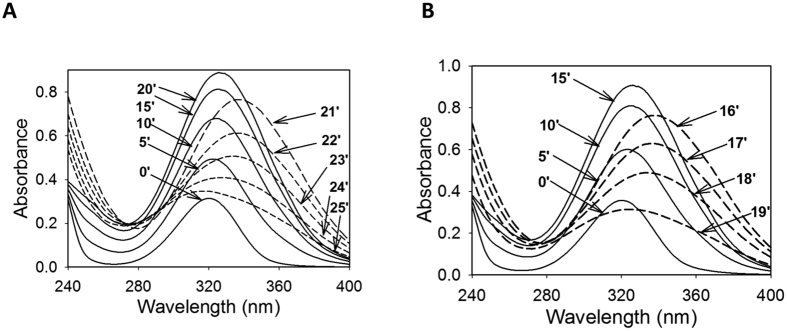# Erratum: Insights into functional and evolutionary analysis of carbaryl metabolic pathway from *Pseudomonas* sp. strain C5pp

**DOI:** 10.1038/srep40899

**Published:** 2017-03-30

**Authors:** Vikas D. Trivedi, Pramod Kumar Jangir, Rakesh Sharma, Prashant S. Phale

Scientific Reports 6: Article number: 3843010.1038/srep38430; published online: 12 07 2016; updated: 03 30 2017

This Article contains an error in Figure 1, where panel A is a duplication of panel B. The correct Figure appears below; the Figure legend is correct in the published version.

## Figures and Tables

**Figure 1:**