# Supplementary material for: Insights into functional and evolutionary analysis of carbaryl metabolic pathway from Pseudomonas sp. strain C5pp
Source: Sci Rep. 2016 Dec 7;6:38430. doi: 10.1038/srep38430 (PMC5141477; doi:10.1038/srep38430)
Supplement: Supplementary Information [file srep38430-s1.pdf]

Supplementary Information

SREP-16-29485
Revised version

Insights into functional and evolutionary analysis of carbaryl metabolic pathway from *Pseudomonas* sp. strain C5pp

Vikas D. Trivedi[1], Pramod Kumar Jangir[2+], Rakesh Sharma[2✉] and Prashant S. Phale[1✉]

[1]Department of Biosciences and Bioengineering, Indian Institute of Technology Bombay, Powai, Mumbai 400076, India.
[2]Microbial Biotechnology and Genomics Unit, CSIR-Institute of Genomics and Integrative Biology, Mathura Road, New Delhi 110020, India.

✉ Corresponding authors: Phale PS, pphale@iitb.ac.in and Sharma R: rsharma@igib.res.in

[+] **Present address**: Synthetic and Systems Biology Unit, Institute of Biochemistry, Biological Research center of the Hungarian Academy of Sciences, Szegad 6726, Hungary

Pseudomonas sp. strain C5pp utilizes carbaryl as the sole source of carbon and energy *via* 1-naphthol, 1,2-dihydroxynaphthalene, salicylaldehyde, salicylate, gentisate and maleylpyruvate[15]. Based on metabolic studies the degradation pathway has been hypothesized to be divided into 'upper' (carbaryl to salicylate), 'middle' (salicylate to gentisate) and 'lower' (gentisate to TCA cycle intermediate) pathway. In concurrent with the metabolic studies, three operonic segments were identified in the draft genome of *Pseudomonas* sp. strain C5pp which are proposed to be involved in the carbaryl degradation[17].

Many microorganisms capable of utilizing carbaryl have been identified, but little or no information is available at the molecular level. This has limited our understanding about the genetic organization, regulation, possible route for acquisition of the genes involved in degradation and evolutionary history of the strain. The sequencing of *Pseudomonas* sp. strain C5pp genome will probably answer some of these questions. Here, in this section, we present an additional data to support the results presented and discussed in the main manuscript.

Though no t-RNA gene or *tra*-elements were identified at 3' or 5' of the 76.33 kb segment identified to be involved in carbaryl degradation, the segment was found to be interspersed with number of insertion sequences, repeats, duplication of ~2.6 kb region containing integrase and skewing of G+C content. This analysis suggests the 'patchwork' in the genetic organization of the pathway which indicates a very recent acquisition of the pathway in *Pseudomonas* sp. strain C5pp.

Analysis of G1 clone

The gentisate utilizing fosmid clone, designated as G1 obtained from genomic library constructed in *E. coli* was analyzed for restriction digestion pattern with *Bam*HI. The restriction digestion of G1 with *Bam*HI gave six fragments of ~12, 8, 5, 2.8, 2.5 and 1.8 kb size. These six fragments obtained after *Bam*HI digestion were sub-cloned into pUC19 and partially sequenced. The analysis and annotation of G1-DNA sequences by BLASTn analysis is given in **Table S1**.

Genome sequencing

The sequencing of strain C5pp genome generated 249174 raw reads and ~98 % of the total reads were assembled *de novo* giving 131 contigs totaling to 6161937 bases **Figure S1A**. The large contigs (97) were used further for genome annotation and analyses. Genome features

and the statistics of assembled genome of strain C5pp are summarized in and subsystem features in **Figure S1B**.

Gap filling of contigs involved in carbaryl degradation

The sequences obtained from six sub-clones of G1 were used as a query against strain C5pp draft genome which retrieved contig number 47 (32.72 kb), 62 (13.65 kb), 61 (14.04 kb) and 76 (2.64 kb). Based on the partial sequences obtained from G1 clone, the contig was expected to be in the following order 62-61-76-47. The contigs 68 (8.2 kb), 83 (1.47 kb) and 92 (0.87 kb) were also proposed to be a part of this assembly and expected to be present at 5' end of contig 62. The assembly of contigs was attempted by gap filling PCR reactions using contig specific primers (**Table S2**). The PCR amplified fragments obtained using genomic DNA as template was sequenced to fill the gaps between the contigs. The contigs were also submitted to ORF finder to identify the probable proteins encoded in the sequence. The proteins were annotated based on blastp analysis. The annotation details for these contigs are summarized in **Table S3**. The order of the contig is 83-68-92-62-76-61-76-47 and the length of this assembled segment is 76334 bp. This is referred as Supercontig-A.

Functional analysis of carbaryl hydrolase

Carbaryl hydrolase (CH) catalyzes the conversion of carbaryl to 1-naphthol and *N*-methylcarbamic acid by hydrolysis of either ester or amide linkage. Partial purification of CH was attempted from *Pseudomonas* sp. strain C5pp grown on carbaryl. The cells (14 g) were harvested at 12 h (OD_{540} - ~1) and cell-free extract was prepared as mentioned in Materials and Methods (see main MS). Lysate (64 ml) was loaded on to Q-Sepharose (40 ml) pre-equilibrated with potassium-phosphate buffer (50 mM, pH 7.5), the enzyme was obtained in the unbound (81 ml) fraction. The unbound pool was loaded onto Phenyl Sepharose (11 ml) pre-equilibrated with potassium-phosphate buffer (50 mM, pH 7.5). The enzyme activity was observed in the unbound (111 ml) fraction. The unbound enzyme was brought to 50 % ammonium sulphate saturation and loaded onto Phenyl Sepharose (10 ml) pre-equilibriated with potassium-phosphate buffer containing ammonium sulphate (30 %). The bound enzyme was eluted using increasing concentration gradient of ethylene glycol (0-80 %) and decreasing concentration gradient of ammonium sulphate (30-0 %). CH was eluted between 50-70 % of ethylene glycol and 10-5 % of ammonium sulphate. The active fractions (1.5 ml each) were pooled and loaded onto

Sephacryl S200-HR which eluted at 49 ml. The enzyme was purified ~73 fold with yield of 4.3 % and specific activity of 11.3 μmole.min^{-1}.mg^{-1}. SDS-PAGE profile is shown in **Fig. S2**, CH was found to be a monomer with molecular weight ~80 kDa.

The molecular mass of McbA (annotated as hypothetical protein) was predicted to be 83 kDa (Expasy) which is similar to that observed for partially purified CH from strain C5pp (**Fig. S2**). Thus, this hypothetical protein was speculated to be CH. McbA was cloned in pET28-a(+) at *Nhe*I and *Xho*I site and (**Fig. S3AI & II**) expressed in *E. coli* BL21(DE3). The protein expression was optimum at 100 μM of IPTG and 18 ^{o}C for 16 h (**Fig. S3AIII**) with the majority of the protein in the inclusion bodies. At higher concentration of IPTG and higher temp (30 or 37°C) the protein was found to be present in the inclusion bodies. Even at low concentration of IPTG (5 μM) and lower temperature (18°C) the majority of the protein was found to be present in the inclusion bodies. Different strains of *E. coli* C41(DE3) or Codon plus RIL were failed to improve the expression in cytoplasmic fraction. The lysate after induction with IPTG was assayed on HPLC to detect the formation of 1-naphthol. HPLC (Agilent 1200 series with DAD) was performed using RP-C18 column (4.6×250 mm, particle size 5 μm, Eclipse plus C18, Agilent). The column was developed using solvent system methanol: water (60:40 vol/vol, flow rate 1 ml.min^{-1}). The samples were detected using diode array with wavelength set at 280 nm and 322 nm for carbaryl and 1-naphthol, respectively. In HPLC analysis, besides carbaryl peak at RT 4.4 min, the reaction mixture at the end of 30 min revealed an additional peak with RT 5.2 min which corresponds to the standard 1-naphthol (RT 5.2 min) (**Fig. S3B-E**). The lysate from *E. coli* transformed with vector pET-28a(+) failed to show any peak at RT 5.2 min. These results suggest that *mcb*A codes for CH which converts carbaryl to 1-naphthol.

Significant divergence at the functional (amidase or esterase) as well as at the sequence level has been observed for the CH. The phylogenetic analysis revealed the clustering of CH from strain C5pp with the protein sequences encoding esterases type of enzymes, suggesting the protein to be a member of esterase. The amino acid sequences of CH from strain C5pp, *Rhizobium* sp AC100 and *Pseudomonas putida* were compared with functionally characterized esterase belonging to fifteen different families (**Fig. S4**). Following enzymes were used for phylogenetic analysis, Family 1; Triacylglycerol lipase (*Rhodoferax ferrireducens*) [ABD71067], probable triacylglycerol lipase (*Chromobacterium violaceum*) [AAQ60384], lipase (*Aeromonas hydrophila*) [YP_855045], triacylglycerol lipase (*Pseudomonas aeruginosa* PAO1)

[CAA44997], LipA (*Burkholderia multivorans*) [AAZ39650], lipase precursor partial (*Staphylococcus xylosus*) [AAU88142], thermostable lipase (*Geobacillus zalihae*) [AAO92067], triacylglycerol lipase (*Geobacillus thermocatenulatus*) [CAA64621], Family 7; PnbA (*Bacillus amyloliquefaciens* subsp. plantarum str. FZB42) [YP 001422731], carboxylesterase (*Bacillus pumilus*) [AAU04567], thermostable carboxylesterase Est50 (*Geobacillus stearothermophilus*) [AAN81910], Family 4; Esterase (*Pyrobaculum calidifontis* JCM11548) [BAC06606], carboxylesterase-estA (*Archaeoglobus fulgidus* DSM4304) [AAB89533], Esterase (*Acidicaldus* sp. USBA-GBX-499) [AIE44670], lipase (*Bradyrhizobium* sp. BTAi1) [YP_001240185], Family 9; lipase class 2 (*Shewanella halifaxensis* HAW-EB4) [YP 001674955], Family 12; Lipase (uncultured bacterium) [ACB11220], Family 3; Triacylglycerol lipase (*Kineococcus radiotolerans* SRS3021), triacylglycerol acylhydrolase (*Streptomyces* sp.) [AAB51445], lipase precursor (*Streptomyces albus*) [AAA53485], Family 2; outer membrane esterase (*Salmonella enterica* subsp. enterica serovar) [AAC38796], triacylglycerol lipase (*Photorhabdus luminescens*) [CAA47020], Family 11; probable lipase (*Rhodopirellula baltica* SH1) [CAD74552], probable lipase (uncultured bacterium pFosLip) [ABE69172], Family 8; Esterase III (*Pseudomonas fluorescens*) [AAC60471], carboxylic ester hydrolase (*Arhtrobacter globiformis*) [AAA99492], Family 10; Conserved hypothetical protein (*Bacteroides fragilis* YCH46) [BAD47626], conserved hypothetical protein (*Bacteriodes fragilis* NCTC9343) [CAH06543], hypothetical protein TM0336 (*Thermotoga maritime* MSB8) [NP_623858], Family 14; Acetyltransferase (*Caldanaerobacter subterraneus* subsp. tendcongensis MB4) [NP_623858], lecithin:cholesterol acetyltransferase (*Thermobacter kivui*) [AIS53217], Acetyltransferase (*Thermoanaerobacter kivui*) [WP_049685837], Acetyltransferase (*Thermoanaerobacter ethanolicus*) [WP_003870690] Family 6; Esterase II (*Pseudomonas fluorescens*) [AAC60403], serine esterase (*Arthrospira platensis*) [AAB30793], esterase (*Rickettsia proazekii* str Madrid E) [CAA72452], Family 13; Thermostable carboxylesterase Est30 (*Geobacillus stearothermophilus*) [AAN81911], EstA (*Geobacillus thermoleovorans*) [ABB90597], EstOF4 [AGK06467], Esterase (*Bacillus cereus*) [AAX86643], Family 15; EstGtA2 (*Geobacillus thermodentrificans*) [AEN92268], Family 5; Alpha/beta hydrolase fold protein (*Sulfolobus acidocaldarius*) [WP_011279001], esterase (*Sulfobolus solfataricus*) [CCQ48704], triacylglycerol lipase (*Psychrobacter immobilis*) [CAA47949], triacylglycerol lipase (*Psychrobacter* sp. 7195) [CAJ76164], lipase partial (*Fervibacterium changbaicum*)

[ABL95965], alpha/beta hydrolase fold protein (*Fervidobacterium nodosum*) [WP_011993782]. The functionally characterised CH from *Pseudomonas* sp. strain C5pp and *Rhizobium* sp. AC100 grouped together as a separate cluster alongwith their homologs having a unique conserved motif or signature sequence {W-X-S-[AGST]-D-X-H-[ILV]-H-[AIL]-X(3)-[APST]}, suggesting it to be a new family of esterases (**Figure S4; and Figure S5**).

Functional analysis of 1,2-dihydroxynaphthalene dioxygenase

The sequence alignment of the putative McbB with 12DHNDO from *Burkholeria* sp. showed 68% identity and three conserved motifs- DHY, DHG and GXSH (**Fig. S6A**) essential for the ring-cleavage activity[26]. The phylogenetic analysis reveals that McbB clustered with type-II EDO of *Burkholderia* and *Ralstonia* (**Main text, Fig. 4).** So far, there are no reports on 12DHNDO from *Pseudomonas* sp. which belongs to type-II EDO. To validate *mcb*B encodes 12DHNDO, the gene was cloned into pET-28a(+) at *Nco*I and *Xho*I site (**Figure S6B-I**) and expressed in *E. coli*. The protein expression was optimum at 10 μM of IPTG and 30°C for 4 h (**Figure S6B-II**). The CFE of IPTG-induced *E. coli* cells harboring pET28a-12DHNDO construct showed 95 nmole.min^{-1}.mg^{-1}. Uninduced or *E. coli* cells carrying vector alone failed to show any activity. The recombinant 12DHNDO was partially purified using Ni-NTA matrix (Qiagen, USA) following batch method. Briefly, CFE (750 μl) was incubated for 45min with Ni-NTA (gel vol. 500 μl) pre-equilibrated with potassium-phosphate buffer (50 mM, pH 7.5). The matrix was washed twice with potassium-phosphate buffer (750 μl, 50 mM, pH 7.5) to remove unbound proteins. The bound 12DHNDO was eluted using step gradient of 25, 100, 250 and 500 mM of imidazole in potassium-phosphate buffer (750 μl, 50mM, pH 7.5). The fractions were analyzed on SDS-PAGE for purity and yield. The optimum elution (~85 % purity) was observed with 500 mM imidazole. The imidazole from partially purified 12DHNDO was removed using 10 kDa Centricon (Pall, USA). Partially purified enzyme by Ni-NTA chromatography (**Fig. S6B-III**) showed the activity of 1.9 μmole.min^{-1}.mg^{-1}.

Functional analysis of 1-naphthol 2-hydroxylase

The gene for 1-naphthol 2-hydroxylase (1NH), *mcb*C was cloned into pET-28a(+) at *Eco*RI and *Nde*I site (**Fig. S7A-I and II**) and expressed in *E. coli*. The protein expression was optimum at 100 μM of IPTG and 18°C for 16 h (**Fig. S7A-III**). The CFE of IPTG-induced *E.*

coli cells harboring pET28a-1NH construct showed 1.78 μmole.min^{-1}.mg^{-1} as compared to uninduced (0.1 μmole.min^{-1}.mg^{-1}) or *E. coli* plus vector alone cells (no activity). The recombinant 1NH (r1NH) was purified using Ni-NTA matrix (Qiagen, USA).CFE (10 ml) was loaded on Ni-NTA (vol. 6 ml) pre-equilibrated with Buffer-A. Unbound proteins were removed from column by washing with buffer-A (60 ml). Further, column was washed with buffer-A (30 ml) containing imidazole (5 mM) to remove non-specifically bound proteins. The protein was then eluted using a linear gradient of imidazole (5-100 mM) in Buffer-A (vol. 60 ml, flow rate 30 ml.h^{-1}, fraction size 1 ml). Fractions containing 1NH activity (eluted between 70-85 mM of imidazole) were pooled, concentrated to 1 ml and loaded on to Sephacryl S200-HR gel filtration column (vol. 74 ml, void vol. 37 ml, flow rate 5.5 ml.h^{-1}, fraction size 1 ml). Fractions containing 1NH activity were pooled, concentrated and the purity was assessed by SDS-PAGE[40] (**Figure S7B**). The identity of McbC was confirmed as 1NH by comparing its peptide sequences with enzyme purified from strain C5pp. The MALDI-TOF/TOF-MS/MS analysis of recombinant and wild type 1NH showed a mascot score of 226 and 266, respectively, indicating the significant identity with annotated 24DCPM (*mcb*C, gi|746587302) of *Pseudomonas* sp. strain C5pp. The peptide coverage and amino acid sequence alignment of recombinant and 1NH from strain C5pp was identical (**Fig. S7C**).

Phylogenetic analysis of 'upper' pathway - McbD, McbE and McbF and middle pathway – genes McbI, McbJ, McbK and McbL

The carbaryl degradation pathway follows steps similar to phenanthrene and naphthalene pathway from 1,2-dihydroxynaphthalene onwards. Thus, the presence of two promiscuous enzymes – hydrolase and hydroxylase, can allow a strain to utilize carbaryl if rest of the genes are acquired by process of horizontal gene transfer from naphthalene or phenanthrene degrading system in presence of positive selection pressure. One of the non-parametric indicators of horizontal gene transfer is the systematic study of phylogeny. The variation in clustering pattern of 'genes of interest' when compared to the other genes of an organism is a strong indicator of the same. As carbaryl, phenanthrene and naphthalene degradation follow similar steps for degradation, it was speculated that the genes for carbaryl degradation pathway must have been acquired / evolved from these more prevalent systems. Indeed, 12DHNDO in most of the pseudomonads are type-I EDO whereas, McbB (12DHNDO) from strain C5pp was found to belong to type-II EDO (**Main text, Fig. 4**) alogwith PhnC from phenanthrene degrading

Burkholderia sartisoli. Similarly, 'upper' pathway regulator McbG clustered with PhnS from phenanthrene degrading *Burkholderia sartisoli*. Thus studying the phylogeny of rest of the enzymes of 'upper' pathway – McbD; 2-hydroxychromene 2-carboxylate isomerase, McbE; *trans*-o-hydroxybenzylidenepyruvate hydratase-aldolase, McbF; salicylaldehyde dehydrogenase will further aid the understanding. McbD was found to be closely related to homologs from from *Alteromonas* sp. SN2 and *Marinomonas profundimaris* (**Fig. S8A**). Similarly McbE was found to be closely related to the homologs from *comamonas testosteroni* and *Xenophilus azovorans* (**Fig. S8B**). Whereas, McbF was found to closely related to PhnF from phenanthrene degrading *Burkholderia sartisoli* (**Fig. S8C**). This further supports the hypothesis that carbaryl degradation pathway is probably acquired from phenanthrene and naphthalene degrader *Burkholderia*.

Salicylate is a central metabolic intermediate of the aromatic hydrocarbon degradation pathways which can be converted to catechol or gentisate by the action of salicylate 1-hydroxylase or salicylate 5-hydroxylase (S5H) respectively. In *Pseudomonas* sp. strain C5pp, salicylate is metabolized *via* gentisate[15]. Though S5H can be a single component[42] or multicomponent[30,43]. The preliminary molecular studies on strain C5pp suggested it to three-component system[17]. The multicomponent S5H (*nag*GHAaAb) has been functionally proven in *Ralstonia* sp. U2 (formerly *Pseudomonas*) which degrades naphthalene *via*. gentisate involving *nag* gene cluster[44,45]. The sequence analysis of S5H-NagGH showed reasonably high identities with anthranilate dioxygenase from *Burkholderia cepacia* DBO1[46], halobenzoate dioxygenases from *P. aeruginosa* 142[47], terephthalate 1,2- dioxygenase from *Comamonas* sp. strain E6[48], and salicylate 1-hydroxylase from *Sphingomonas* sp. strain CHY-1[49]. Similar observation was made in *Pseudomonas* sp. strain C6[17]. The analysis of S5H from C5pp suggested α, β, ferredoxin and reductase component to be closely related to homologs from *Ralstonia* sp. U2 (91, 84, 63 and 75 %) and *Burkholderia* sp. C3 (90, 85, 66 and 75 %). The phylogenetic analysis of reductase, α, β-component and ferredoxin suggested it to be closely related to *Ralstonia* sp. U2 and *Burkholderia* sp. C3 (**Fig. S8D-G**) The S5H system in *Ralstonia* sp. U2 serves as an intriguing example of genetic/evolutionary parsimony in which two electron transport proteins (ferredoxin and reductase) essential for activity are shared by two enzymes, naphthalene dioxygenase and S5H (monooxygenase), that are separated by multiple steps within naphthalene metabolic pathway. The S5H genes in *Ralstonia* sp. U2 are closely linked and separated by 88bp from *cis*-dihydrodiol naphthalene dehydrogenase, involved in naphthalene metabolism. In case of strain

C5pp, the S5H is separated by 5517 bp and 17291 bp to 'upper' and 'lower' pathway operons respectively. Moreover, strain C5pp is incompetent to utilize naphthalene as carbon source and lacks the gene for naphthalene 1,2-dioxygenase. This further instigates the origin of the S5H gene cluster in strain C5pp and supports the hypothesis of HGT. S5H being a mono-oxygenase, shows high similarity to dioxygenase type of enzymes, it remains to be seen whether S5H has evolved as a result of gene duplication from a dioxygenase.

Analysis of regulators present on Supercontig-A involved in carbaryl degradation

The LysR type transcriptional regulators (LTTRs) are characterized by an N-terminal helix-turn-helix motif involved in DNA binding. The seven highly conserved residues [Ala^{27}, Thr/Ser^{33}, Gln^{34}, Pro^{35}, Ser/Thr^{38}, Leu^{44} and Glu^{45}; numbers in superscript are in accordance with the NahR sequence] found in 70 % of the LTTRs[50] were also seen in McbG, McbH and McbN. In McbG and PhnS, at position 35 a Pro-to-Ser mutation was seen (**Fig. S9B**). The C-terminal halves (NahR, 227-253 a.a.) proposed to be involved in the co-inducer recognition was found to be conserved among PhnS & McbG; NahR & McbH; and HbzR & McbS (region depicted as box in **Fig. S9B**). This paired difference might be due to the different inducers recognized by the regulatory proteins. The residues 100-150 proposed to be involved in the co-inducer recognition are less conserved[50]. However, this region was found to be conserved among PhnS & McbG, NahR & McbH and HbzR & McbS (region depicted as box in **Fig. S9B**). In the region spanning 100-150a.a. PhnS & McbG, NahR & McbH and HbzR & McbN shared 32, 35 and 41 identical residues out of 50, respectively. Similarly, the C-terminal halve (NahR, 227-253 a.a.) also considered to be important for co-inducer recognition showed only one identical residues. Whereas, PhnS & McbG, NahR & McbH and HbzR & McbS shared 15, 21 and 23 identical residues out of 28, respectively.

Supplementary references

41. Laemmli, U.K. Cleavage of structural proteins during the assembly of the head of bacteriophage T4. *Nature* **227**, 680-685 (1970).

42. Suemori, A., Kurane, R., & Tomizuka, N. Purification and properties of 3 types of monohydroxybenzoate oxygenase from *Rhodococcus erythropolis* S-1. *Biosci Biotechnol Biochem* **57**, 1487-1491 (1993).

43. Grund,E., Denecke, B., & Eichenlaub, R. Naphthalene degradation via salicylate and gentisate by *Rhodococcus* sp. strain B4. *Appl Environ Microbiol* **58**, 1874-1877 (1992).

44. Zhou, N.Y., Fuenmayor, S.L., & Williams, P.A. *nag* genes of *Ralstonia* (formerly *Pseudomonas*) sp. strain U2 encoding enzymes for gentisate catabolism. *J Bacteriol* **183**, 700-708 (2001).

45. Fang, T., & Zhou, N.Y. Purification and characterization of salicylate 5-hydroxylase, a three-component monooxygenase from *Ralstonia* sp. strain U2. *Appl Microbiol Biotechnol* **98**, 671-679 (2014).

46. Chang, H.K., Mohseni, P., & Zylstra, G.J. Characterization and regulation of the genes for a novel anthranilate 1,2-dioxygenase from *Burkholderia cepacia* DBO1. *J Bacteriol* **185**, 5871-5881 (2003).

47. Tsoi, T.V. *et al*. Cloning, expression, and nucleotide sequence of the *Pseudomonas aeruginosa* 142 *ohb* genes coding for oxygenolytic ortho dehalogenation of halobenzoates. *Appl Environ Microbiol* **65**, 2151-2162 (1999).

48. Sasoh, M. *et al*. Characterization of the terephthalate degradation genes of *Comamonas* sp. strain E6. *Appl Environ Microbiol* **72**, 1825-1832 (2006).

49. Jouanneau, Y., Micoud, J., & Meyer, C. Purification and characterization of a three-component salicylate 1-hydroxylase from *Sphingomonas* sp. strain CHY-1. *Appl Environ Microbiol* **73**, 7515-7521 (2007).

50. Schell, M.A. Molecular biology of the LysR family of transcriptional regulators. *Annu Rev Microbiol* **47**, 597-626 (1993).

51. Gough, J., Karplus, K., Hughey, R., & Chothia, C. Assignment of homology to genome sequences using a library of hidden Markov models that represent all proteins of known structure. *J Mol Biol* **313**, 903-919 (2001).

Supplementary Figures

(A)

**Genome sequencing data**	
Total no. of bases	186,421,524
Total no. of reads	2,49,174
Number of large contigs	97
Average contig size	63445
N50 contig size	147962
Largest contig size	345732
Coverage	26X
**Genome features**	
Genome size	6.15 Mb
G+C content (%)	62.65%
Protein coding sequences	5697
Total no of genes	5588
rRNA	4
tRNA	68

(B)

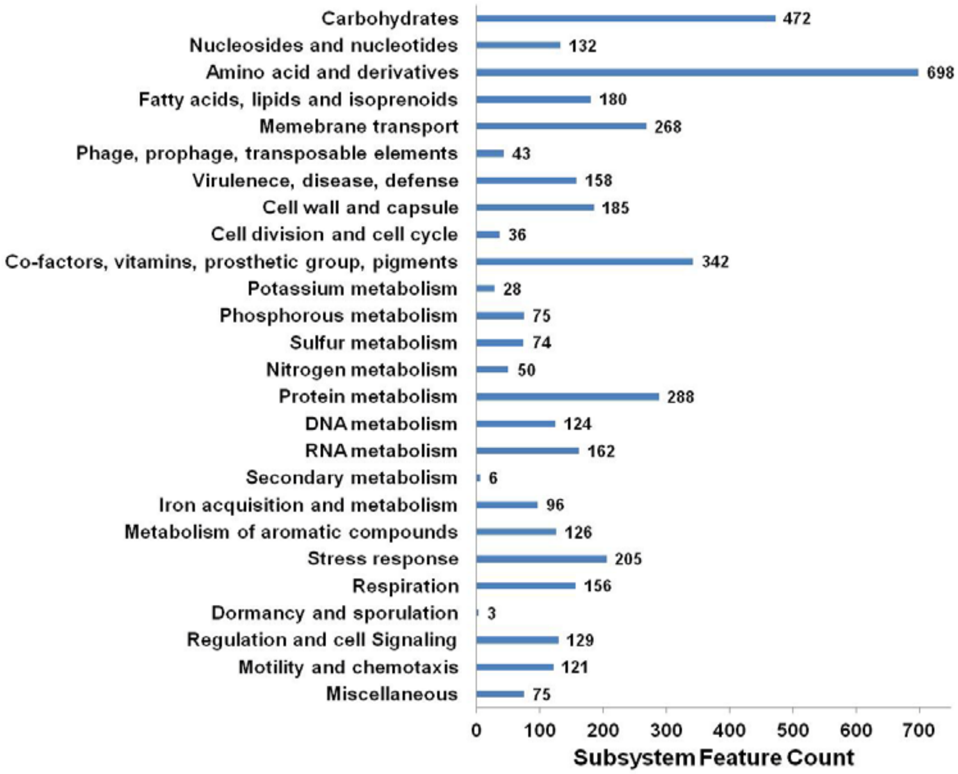

Figure S1: Genome sequencing and features of *Pseudomonas* sp. strain C5pp are depicted in panel (A) while subsystem features are depicted in panel (B). Numbers indicate the gene count.

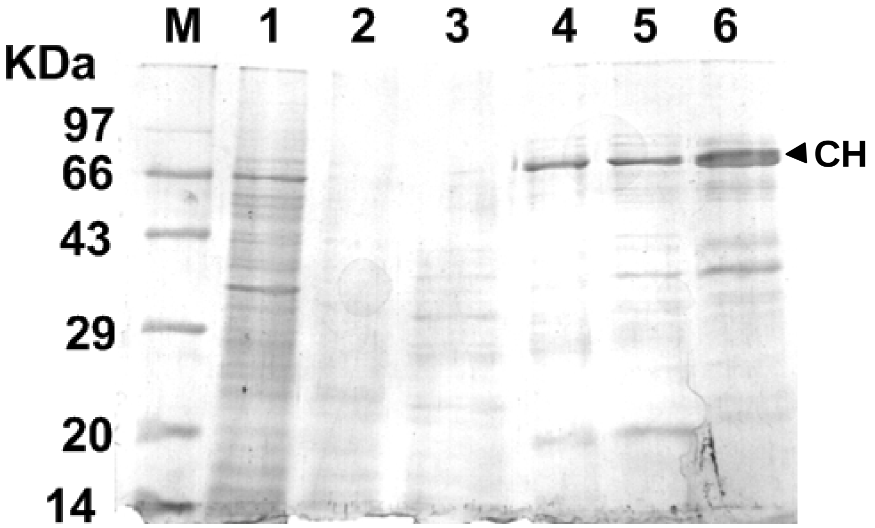

Fig. S2: Partial purification of carbaryl hydrolase (CH) from *Pseudomonas* sp. strain C5pp. CH was purified from cells grown on carbaryl (0.1 %). Lanes: 1, lysate; 2, Q-Sepharose unbound; 3, Phenyl-Sepharose unbound; 4, 50 % ammonium sulphate saturated supernatant; 5, P-Sepharose fraction; 6, Sephacryl S200 fraction. ◄ indicates protein band for CH.

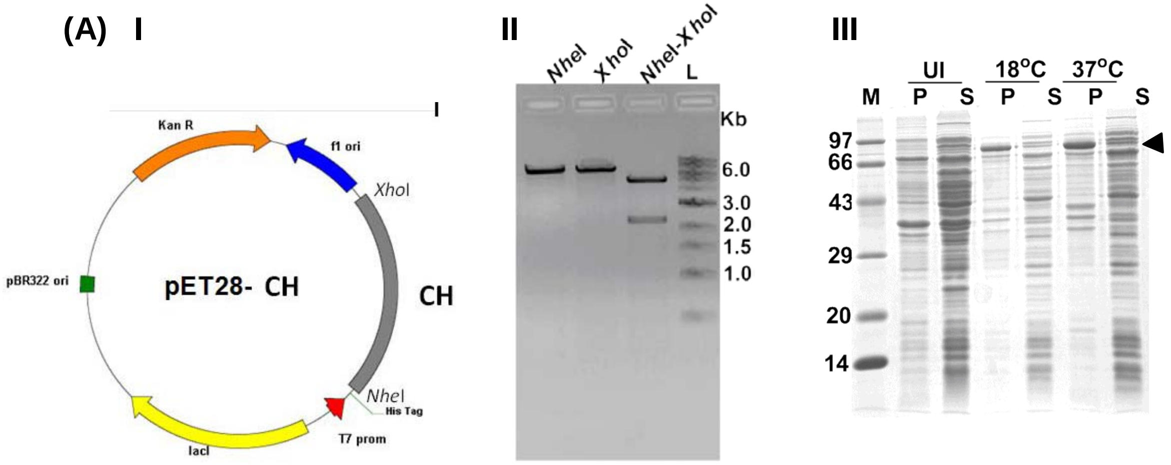

(A) I
II
III
Kan R
f1 ori
XhoI
pBR322 ori
pET28- CH
CH
NheI
His Tag
T7 prom
lacI
NheI
XhoI
NheI-XhoI
L
Kb
6.0
3.0
2.0
1.5
1.0
UI
18°C
37°C
M
P
S
P
S
P
S
97
66
43
29
20
14

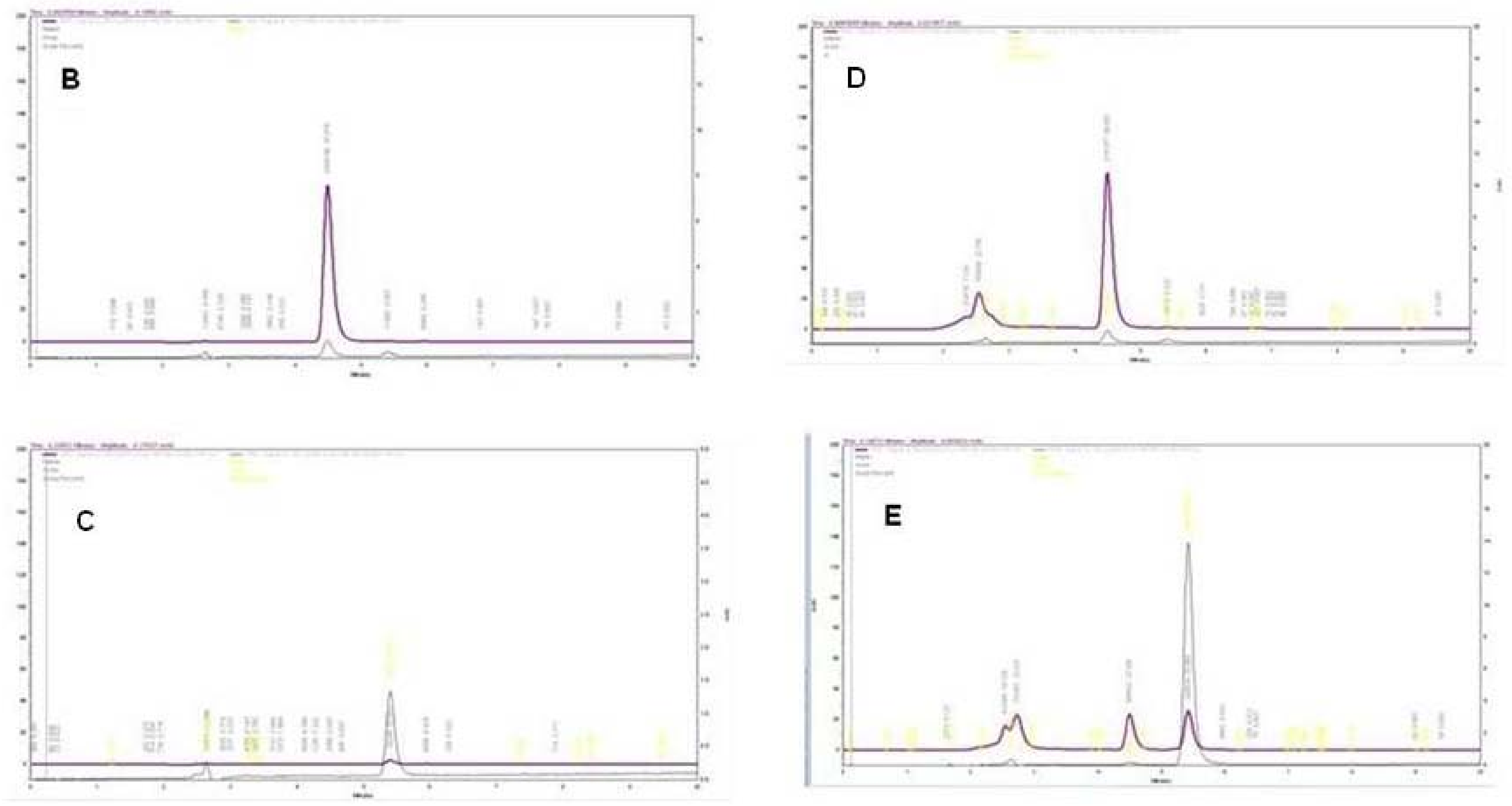

Fig. S3: Functional analysis of carbaryl hydrolase. Cloning and expression of carbaryl hydrolase (CH): (**A-I**) A map of CH construct, pET28a-CH and (**A-II**) the release of 2.3 kb insert after restriction digestion with *Nhe*I and *Xho*I. (**A-III**) SDS-PAGE analysis of CH expression from uninduced ‘UI’ and induced ‘I’ *E. coli* BL21(DE3) harboring pET28a-CH. Induction was carried out using 100 µM IPTG at 18°C for 16 h. ‘P’ indicates pellet or insoluble protein fraction, ‘S’ denotes supernatant or soluble protein fraction and ‘M’ indicates protein mol. wt. markers. Solid arrow head indicates the CH protein (~83 kDa). HPLC analysis for carbaryl hydrolase. The standard carbaryl (**B**, 400 µM, purple line), 1-naphthol (**C**, 50 µM, black line) showed a retention time of 4.4 min and 5.2 min, respectively. The HPLC profile of reaction mixture of lysate prepared from (**D**) *E. coli* BL21(DE3)-pET28 and (**E**) *E. coli*

BL21(DE3)-pET28a-CH. The reaction mixture containing lysate from *E. coli* BL21(DE3)-pET28-CH showed an additional peak corresponding to RT 5.2 (1-naphthol) in comparison to the reaction mixture containing lysate from *E. coli* BL21(DE3)-pET28 which showed single peak of RT 4.4 (carbaryl).

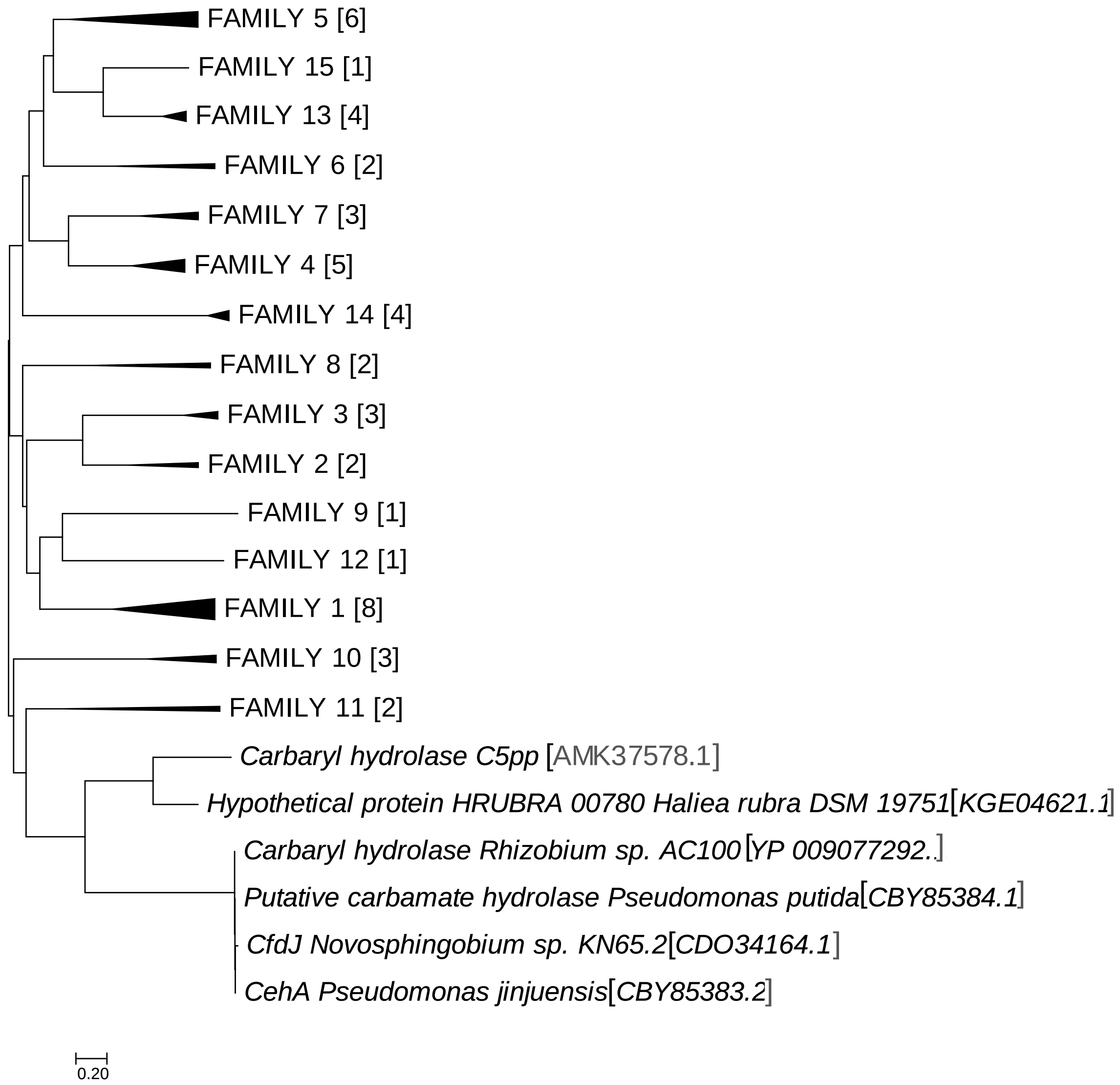

Figure S4: The phylogenetic analysis of CH from *Pseudomonas* sp. strain C5pp with members of fifteen different families of esterase. The numbers in brackets indicates the number of that particular family included for phylogenetic analysis.

```
                                        10        20        30        40        50        60        70        80        90       100       110
                                ....|....|....|....|....|....|....|....|....|....|....|....|....|....|....|....|....|....|....|....|....|....|
H rubra DSM19751                ------------------------------MRTLAMVRSTCARSILESRKS-----SISPCAFIAR----------AACLVMVGILSLGKTDVAEAHAGHPLGVKAIAWH
A algicola                      --------------------------------------------------------------------------------------------------------------
C Solibacter                    --------------------------------------------------------------------------------------------------------------
T bouteillei VB521301           --------------------------------------------------------------------------------------------------------------
R lutea                         --------------------------------------------------------------------------------------------------------------
F limi BUZ 3                    --------------------------------------------------------------------------------------------------------------
S linguale DSM 74               --------------------------------------------------------------------------------------------------------------
Rhizobium sp. AC100             ------------------------------------MDQPFKPDRRQLLAGATLLLGASHWPVFASTDAIEPQPYFASVKRAVEQLAAMGQPIADADMARLASLFSRED-
P fluorescens                   ------------------------------------MDQPFKPDRRQLLAGATLLLGASHWPVFASTDAIEPQPYFASVKRAVEQLAAMGQPIADADMARLASLFSRED-
Poribacteria bacterium WGA-3G    ------------------------------------MRFVLIGWMLLSFFIS--MGS---ADELDRVAGIEFQPLAAATQRLIEALDYLGSPLSENDLAAIEVALMSEDH
S acidiphila DSM 18658          ------------------------------MSLPSVWRRSLSPGLLLLLLAG--TAAPAHADRLPQVDEVEFQPLSAQVRRVVEAYDLLGQPLPPRLKARLENALNGSDQ
A bacterium KBS 96              ----------------------------------MAIKSFLLPLALAAFALPPLWAQAPHVHPADALPPVPLQPLAQQVRRLEDALSFLGQPLPARTRAAINSAIAAPDE
C flavus                        ----------------------------------MNSRFILLAAFLAPLVG--------FAEDLPVITGVEPQPLTAQVHRLTDALALLGSPLTKDEMDALTVAAAVADP
S paludicola KE                 -------------------------------------------------------------------MGRVEQQPLAAATERLIQALEFVGSPMDAETRTKLEESLKLPSA
Rhizobium sp. AC100             ------------------------------------MDQPFKPDRRQLLAGATLLLGASHWPVFASTDAIEPQPYFASVKRAVEQLAAMGQPIADADMARLASLFSRED-
Pseudomonas sp C5pp CH          MAVTANYLRGALAALCVGVSLVGVGQFRLGVESLKWLSPSITRPNPRPEKCRRGSATVAKNGAPARSTIRERHMYVTRLIIHAAIASLMASGPALAHSTGPLGKIEHGWK
Clustal Consensus

                                       120       130       140       150       160       170       180       190       200       210       220
                                ....|....|....|....|....|....|....|....|....|....|....|....|....|....|....|....|....|....|....|....|....|....|
H rubra DSM19751                PDPVIGRTLALNYDAHAGRFIIARRNVGSLVKVEGRDCLTGPYFLFDIRDEFAFDEDATAKLELLFRNTGATGAYVTYDHVIEPDGREVRFDQASREPWHRVTVELDRAR
A algicola                      --------------------------------------------------------------------------------------------------------------
C Solibacter                    --------------------------------------------------------------------------------------------------------------
T bouteillei VB521301           --------------------------------------------------------------------------------------------------------------
R lutea                         --------------------------------------------------------------------------------------------------------------
F limi BUZ 3                    --------------------------------------------------------------------------------------------------------------
S linguale DSM 74               -----------------------------------------------------------------------------------------------MLRTWLAYLHGCTVD
Rhizobium sp. AC100             AEAVIAAEALLERYTLARVTLDKGGMGAAALGGAKRHLVEQGWTAFLVRVSNPSSLTQAFEIYSGSAMPARMFPQASKFAAE--QKAYLADTVR---KEALIERMWLMSQ
P fluorescens                   AEAVIAAEALLERYTLARVTLDKGGMGAAALGGAKRHLVEQGWTAFLVRVSNPSSLTQAFEIYSGSAMPARMFPQASKFAAE--QKAYLADTVR---KEALIERMWLMSQ
Poribacteria bacterium WGA-3G    EQAIADIQKILDAYCLAGVNINPESRVKVKEGPVNKELMQQGWRTFLVKVHNEAGVTAPLAAESENAAPLYKRSTGSPDP----ETTVPKSEIP-----HRFLEIHVYSD
S acidiphila DSM 18658          AQAVHEIQEILDPLCLIGVTINPESRVKVAPGPADANLVQHGWRVFLVKVQNEAGVTAPLAAQSPNAAPVYRRSTGRPDP----KPTVSPADVI-----QRWMDLALFED
A bacterium KBS 96              LAAVTQIETALEPFVLAVVNINGESRVKVERGPAQPALVQSGSRFFLVKIINQGNVTSALNVASPNSGRVFVP--SKNDPSP--KMVLTKADVR-----DRWADISIYNK
C flavus                        ESANAKIQAVLDPHCLFGVDINPEMRVKVVPGRAQPILVERGWATFLVKVANESGATSALNVYSPQAESVYSGGFVRNASDL--KLRPRNGNAEPIKAADLWLDLQTFDK
S paludicola KE                 ADCTEAVEKILDPLCLVAVSINPESRVSVVEGPVKKELVQQGWRTFLVKVHNEAGVTAPLVPESPNLLPVYQRGKKAREKPMTDEKLVQPSDVP-----NRFLDADMFDK
Pseudomonas sp C5pp CH          PSPEIKRTVSLNFDFHGGGNFVSWSPSARMTQHENRQCITGAYLLFDVDDRFAFDIDETILVDLTFFRPETDGFVFSYDQAVQPTAKKVRFDANNKGSWHTETVRLDRAR
Clustal Consensus

                                       230       240       250       260       270       280       290       300       310       320       330
                                ....|....|....|....|....|....|....|....|....|....|....|....|....|....|....|....|....|....|....|....|....|....|
H rubra DSM19751                FANRKYGGTDFGIAAAGAAGAGTPGRDHQVTLCDLKLTLDRS-ARSPESGELVLSLADDAGKAVTARVGIYDEEG-----RAPLPGKSAMIIDRYSDRVRHLSLLGSKGT
A algicola                      -------------------------------------------------SIKVTVTDQNTGKPTAVRAKITNSD-------GYVAG------IPKEAISIMYGRDDKAER
C Solibacter                    ------------------MRSSMAVRVGVAGVFG--FALVQFAQVSREATLTVRIVDAATGRATPVRVRLEDSNRVRPRVRGAVAVSDSAIPVPKQAIAVMWGQNDRAQG
T bouteillei VB521301           -------MSRFRILDRNRWRGGSLLASLLVGWLG---LVSAVAQTAQSGELVVEVVTGNPTKVTPVRVRLTQQG-------RAVKL------LPPEAIGVMYGLWDHADG
R lutea                         ----------------------MLALGLALG-------GWPRALAQRAGQLRVVVRDAATGKPTPVRVRLTRAGG------GPVPV------VPPEVVAVQYGLWDHADG
F limi BUZ 3                    --------------MHRSWQLTIWLFLVLSGFHLVR-AQSTPERTYKPSRIEVQILDATTKRPTPARIRITQNG-------RVVPL------LPTEAVAVMYGLWDHEDG
S linguale DSM 74               GTAHRNTTAGQPRPLYNGCSTTRFAIGLLCGILCLTSLNDTRAQGKPPGSVHIAVRDGKTGKSTPVRVRLTQAG-------KVVKA------LPAQVAAVSYGLWDHADG
Rhizobium sp. AC100             IEASPVLSGNVVEYKIATIYSRDRGRKTGRLGFGAGILQDNPTWGAVSRASVANPLVTDFVSQPSRDIQLQILD--------DDGVGCMASLMIHDANKHIYPPQVMRLA
P fluorescens                   IEASPVLSGNVVEYKIATIYSRDRGRKTGRLGFGAGILQDNPTWGAVSRASVANPLVTDFVSQPSRDIQLQILD--------DDGVGCMASLMIHDANKHIYPPQVMRLA
Poribacteria bacterium WGA-3G    PPLKASLSGLELEYRIVQLYSRDAGKREAMLGFNVG--QGTQDIGFRSEVPIL------FDCVPAVNVTLEVLD--------FDGQPTTAGFIIRDALNRVYPSRGRRLA
S acidiphila DSM 18658          RPMSRSLSGLALEYRLLQIASRDSGQREAKIRFDVG--QGSQDLGSRSEVDIL------FRCEPAVPVTLDVQDE------NDDGRPVTASFLFRDAQGRVYPSQTRRLA
A bacterium KBS 96              PPMETRLSGLAVEYQILEIYSRDAGQRSADISFNVG--QGTQDIGFRNDMLVL------FHADAARQIKLHVLD--------ENGKPSIASLTIRDRWNRLYPNPSKRLA
C flavus                        PPLKATLSGLRLEYRLVSLYSRDAGQREATLSVDVG--QGTQDLGFRSEMPVL------FTAKPAREIELRVHD--------EHGVPTTGSFLIHDQAQRVYPSTAKRLA
S paludicola KE                 QPLKPGLSGLELEYRILQLYCRDVGRREVSLGFNVG--QGTQDLGFRSTVPLL------FNSVLAVEVVLGIKD--------FDGTPTFAALTFRDARGRVYPNPSRRLA
Rhizobium sp. AC100             IEASPVLSGNVVEYKIATIYSRDRGRKTGRLGFGAGILQDNPTWGAVSRASVANPLVTDFVSQPSRDIQLQILD--------DDGVGCMASLMIHDANKHIYPPQVMRLA
Pseudomonas sp C5pp CH          FANRKYYGTDFAVGGLGSQLTRS-NDGGEVTLCDVKVRREASKPQAPSPGKFALQVLDEAGRPTAARVGLYRSDG-----WAPLAGRSALTVQRYTEYTRNLPMVSVPKG
Clustal Consensus                                                                               .    :
```

```
                                        340       350       360       370       380       390       400       410       420       430       440
                                ....|....|....|....|....|....|....|....|....|....|....|....|....|....|....|....|....|....|....|....|....|....|
H rubra DSM19751                WPGKGSYVFYVDGHHRQKLPAGRYQLVVGKGPEFHYVNREITIA-EGETLAVTLRPQRWSDLPAAGWYSGDGHIHIARESREKNQ-------GIAALMAAEDIHVANLLQ
A algicola                      YGFQPDSTYYIDGHFNLDLRPGKYKISLSKGFEYLDQAHTIEVK-EGGGNAFSFEMKRWVKMSDLGWYSADDHIHIRR-SPRENP-------LILKWVEAEGLNVGILLQ
C Solibacter                    YAIQPDGSFYVDGAFEVKVPAGRYSLLVTKGNEYTRVTDTVELA-AGAAAAREYKMTRWIDMPGRGWYSSDDHIHLRR-SPGDDH-------AIAQWIAAEDIHVGHLLQ
T bouteillei VB521301          FGFQPDSSFYVNGRFRLKLPAGTYQLSLSKGPEFVDQKHQLVVV-AGKSQKKTLRLTRWIDMAARGWHSTDGHIHIRR-SPREDP-------LLMSWLQAEDVRVGVLLR
R lutea                         YAFQPDSSFYVSGAFSLTLAPGTYDLSLSKGNEYVHQTHRLHIR-SGQVLRQTYALKRWIHMAARNWYSADDHIHIRR-SPRENP-------LLLDWIRAEDVNVGVLLK
F limi BUZ 3                    YAYQPDSSFYVAGKFTLNLPPGTYQLALSRGNEYLRQQQELVVR-PGGAVRKTYTLQRWVNMATRNWYSTDGHIHIRR-SPRENP-------LIMTWLQAEDVNVGALLR
S linguale DSM 74               YAYQPDSSFYVPGQFSLALPAGTYQISLMKGNEYVNQQHTLVVK-PGQMLRKTYTLTRWIHMAARGWYSADDHIHIRR-SPGENK-------SLMQWIQAEDVNVGVLLK
Rhizobium sp. AC100             PDLAFQPHIYRADGETVRLPDGEYHIMVRRGPEYLVQRATVRVS--ASDARIAVRLKRWIDPAKWGWYSGDTHLHAAGCAHYMLPTEGVAPETMIRHIRGEALSVGEVLT
P fluorescens                   PDLAFQPHIYRADGETVRLPDGEYHIMVRRGPEYLVQRATVRVS--ASDARIAVRLKRWIDPAKWGWYSGDTHLHAAGCAHYMLPTEGVAPETMIRHIRGEALSVGEVLT
Poribacteria bacterium WGA-3G    PDFFFHNQIYRHSGESVLLPPGEYTVEYTRGPEYKVKTQTVTVP-NAETHQETFHLERWIHIAAEGWRSGDHHVHAAGCAHYESPTEGVSPEDMMRHILGEDLNVGCVLS
S acidiphila DSM 18658          PDFFFHPQVYRQSGETVLLASGTYTVEYNRGPEYLTLKKTITVP-RAAAHRESFHLKRWAHLAKRNWFSGDHHIHAAGCSHYESPTEGVGPESMMRHILGEDLNVGCVLS
A bacterium KBS 96              PDFFFQPQIYRADGESIELPPGYYTVVATGGPEYNQHTKEFSVDAKGPDDLS-FKLDRWIDPSKFGWYSGDHHVHAAGCSHYQNPAEGVLPKDMMRQISGEKLNVGAVLT
C flavus                        PDFGFQPQIYRADGDKIRLPDGNYTIEFSRGPESIPETRPVHVDA-GTSELA-FQVRRWIDLAQLGWWSGDHHIHAAGCAHYTNPTEGVLAQDMMRHCLGEDLKVGCNLT
S paludicola KE                 PDFFFHEQIYRGDGESVMLPPGDYTVTVQRGPEYRVDQLPLKVTGNAVSQRQEISLRRWIHAAKRGWISGDHHVHAAGCSHYDSPTEGVTPGDMLRHILGEDLNIGCVLS
Pseudomonas sp C5pp CH          WSANGRYVFFVDTNYAADVPAGEYDLFVMKGPEYRIQKRKVRIA-PGTPQSIQVKMERWTDLPKQGWLSGDDHIHIGRSTPAENDP-------KILAYMRAEDLGVANLLE
Clustal Consensus                            :        :  * * :     * *        . :    .        **  .  .  .* * * *:*    :              :     .* : :.  *

                                         450       460       470       480       490       500       510       520       530       540       550
                                ....|....|....|....|....|....|....|....|....|....|....|....|....|....|....|....|....|....|....|....|....|....|
H rubra DSM19751                MGNLRRWYYPQYAFG---------------------EAGVFQSGSYLLASGQEAP--RSAHLGHTIGLNASSFHWDENR-----QFIYSDVAEALREGGVFGYAH---
A algicola                      MGDFWATYFSQYAFG---------------------EDGTYGQNESLLSTGQEDP--RTHQIGHTIALVADDFVRQQKD-----YYEYDKIFDKVHELNGITGYAH---
C Solibacter                    MGDFWTLYFAQYAFG---------------------ERGRYAEAGRILSPGQEDP--RTPEIGHTISLGADDFARAQGD-----YYSFDRLFDRVHELGGVSGFAH---
T bouteillei VB521301          MGDFWETYYPQYAFG---------------------ESGVYRKDNYLLVSGQEDP--RTPELGHALGYGASDKVRFSKE-----YYHYDTVFDKLHQLGGLTGYAH---
R lutea                         MGDFWETYYPQYAFG---------------------EKGMYQQGPFLLMSGQEDP--RTPELGHALGFGASGSVRYRQD-----YYLYDRVFDELHRRGGLTGYAH---
F limi BUZ 3                    MGDFWETYYPQYAYG---------------------EKGNYQQGNYLLTTGQEDP--RTPELGHAFAIGASNRVRQRDR-----YYFFDEVFDKIHQLGGLTGYAH---
S linguale DSM 74               MGDFWETYYPQYAFG---------------------VAGNHRDGNYLLTPGQEDP--RTPELGHALGFGATGSVRYKQD-----YYYYDKVFDELHKRGGLTGYAH---
Rhizobium sp. AC100             WAGGYSYQKQFFSGAAISPEARLEHPQLQAANNADWKPMPTPKDRESMLRYDLEISGFPSSLSGHLILMRLKELSYPGTHAVEDWPSWNLPVAKWAKAQGALVGYTHCSF
P fluorescens                   WAGGYSYQKQFFSGAAISPEARLEHPQLQAANNADWKPMPTPKDRESMLRYDLEISGFPSSLSGHLILMRLKELSYPGTHAVEDWPSWTLPVAKWAKAQGALVGYTHCSF
Poribacteria bacterium WGA-3G    WGPCWYFQKQFFDG-------------------KVHELSTD---DYVMRYDVEVSGFPSSHAGHLSLLGLTEDDYSGTERIEEWPSWDLPVLQWAKEQGAVTGFSHSGW
S acidiphila DSM 18658          WGPCWYAQKANFDG-------------------KLHPLSTP---ASLMRYDIEVSGFPSSHAGHLCLLRLKEDDYPNTTRIEEWPSWDLPVLTWGKSQGGVVGFSHSGW
A bacterium KBS 96              WGPDYYYQKQFFSG-------------------HDDPLSLP---NERMHYDLEVSGFPSSHAGHLVLLGLKDQDYPHTRKIEDWPTWDLPILKWAKQQGAVVGFAHSGW
C flavus                        WGPCFDYQKQFFTG-------------------HEDKVSQF---PYLLRYDIEVSGFGSHRSGHLCLLRLRDQIYPGGDSKNHWPTLGLNTLRWAKAQGALVGPAHSGW
S paludicola KE                 WGPCWYTQKRYFEG-------------------KTSALSRP---NYLMRYDVEVSGFPSSHAGHLCLLKLIEDDYPGTTLLEEWPSWTQPVLEWGQKQGGVVGYSHSGW
Pseudomonas sp C5pp CH          AGNLKGSQFKQYGFG---------------------KEGHYGNGGHFLVSGQESP--RTSHRGHSIGLNAKKFHWPEKD-----YFIYDATSDMIRADGGLWGYAH---
Clustal Consensus                 .          :                                        :  . * .    :    **                               :  ..: * :*

                                         560       570       580       590       600       610       620       630       640       650       660
                                ....|....|....|....|....|....|....|....|....|....|....|....|....|....|....|....|....|....|....|....|....|....|
H rubra DSM19751                ---------------------------------VALADSFELQRGLPLGVALGYVDFL------------EILQMGVLN----TRLYYDFLNLGFRLLPSAGSDYPYI--
A algicola                      ---------------------------------QGM--SFNGSRGMTLDVLSGKVDFL------------ELLQFCVNGGPLLTQNYYHFLDLGYKLTATAGSDFPWCGL
C Solibacter                    ---------------------------------QGM--SFHGYRGMALTTLRGKTDFL------------ELAQFCVPEGPLALEHYYEFLDLGIKLTALAGSDFPWCGQ
T bouteillei VB521301          ---------------------------------HAE--TFHGYRGLTLDGLRGKVDVL------------EILQYCVDANPLRTDHYYHLLDLGFPVTATAGSDFPWCGQ
R lutea                         ---------------------------------QAE--SFHGYRGLTLDGLRGKVDLL------------EILQYCVSADPLRTEHYYHLLDLGIPLTATAGSDFPWCGH
F limi BUZ 3                    ---------------------------------QAE--TFHGYRGLILDGLRGKVDAL------------ELLQFCVDEQPLHTEHYYHMLDLGIPLTATAGSDFPWCGQ
S linguale DSM 74               ---------------------------------QAE--SFHGYRGLTLDGLRGKVDML------------ELLQYCVSDEPLHTENYYRMLDLGFPLTATAGSDFPWCGH
Rhizobium sp. AC100             GMIVG--------------------------TNALPNYEIPTFNSVGTNEAIVGVTHG----------AVDFLAGCNGPPAAELNAWYHLLNCGYTLMMAGETDYPCIVP
P fluorescens                   GMIVG--------------------------TNALPNYEIPTFNSVGTNEAIVGVTHG----------AVDFLAGCNGPPAAELNAWYHLLNCGYTLMMAGETDYPCIVP
Poribacteria bacterium WGA-3G    GLKV--------------------------DGDELPNYNMPPFDGIGANEYIVDVVHD----------AVDFISTVDTPAVWELNIWYHTLNCGFRTRISGETDFPCIY-
S acidiphila DSM 18658          GLKT--------------------------ADGKLPSYEIPPFDGIGANEYIVDVVHD----------AVDFISAVDTPAPWELNIWYHTLNCGYRTRISGETDFPCIY-
A bacterium KBS 96              GLQ--------------------------VSDRALPSFEMPGFDGIGANEYIVDVT----EPD-----AVDFISAGDTPFVWELSIWYHTLNVGFRTRIAGETDFPCIT-
C flavus                        GLQP----------IPEDDPQRLSPKNPTRTTTELPNYIIPPFSGIGANEYIVDVTHKLPGPDGKPTPAVDFMSMVDTPYPWELNIWYHTLNVGFRTRISGETDFPCIY-
S paludicola KE                 GLALPDVMPDGSRKFSDKPNGGASAGWQGKAAEKLPDLAMPRFDGIGANEFIVTATNG----------TCDFISAVDTPAIWELNIWYHTLNCGMRTRISGETDFPCIY-
Pseudomonas sp C5pp CH          ---------------------------------VAL-DIFNVDYGLALDVPRGKVDFV------------EMLQYGTMN----TAYLYDFLNMGFKLLPSAGSDYPYI--
Clustal Consensus                                                   :     .:         .            ::               *  *: *      . :*:*
```

```
                                        670       680       690       700       710       720       730       740       750       760       770
                                ....|....|....|....|....|....|....|....|....|....|....|....|....|....|....|....|....|....|....|....|....|....|
H rubra DSM19751                ------------DVAGSDRLYVKLT----KPFSRENWFSAWTSGRSFVSNA--PVIEFSVN-------------------------GDEAGSELSLERGAT------IA
A algicola                      GPLFGTEDPSWNARIGNVRFYTWID----GKFEYASWKKNLKEGHTFVSSG--PMLVFSID-------------------------DHLPGDEIKLSAPKK------VS
C Solibacter                    GP--GP-----AAQIGNARFYTYTG----GELNFARWMAGMKAGHTFVTTG--PLLTLTVN-------------------------RKMPGDSVDVTKGST------VS
T bouteillei VB521301           DHNHGS--PEHSARIGNARFYVFTE----KPLTNQAWKEGLKAGHTVVTSG--PMLDFRIN-------------------------NARPGDRLNVKKEEK------LT
R lutea                         DHDHGP--PEHSARIGNARFYTYIN----GPLTPAAWQAGVAAGHTFVTSG--PILDLQVNSVA----------------------KQGTSRPGDRVAVKKGST------LT
F limi BUZ 3                    DHGHGP--PERTSRIGNARFYTFIN----GPLTYQNWRKAVQTGHTFVSSG--PILDFSATTAD-------------------RR-KAIPGDQLDLKRTDK------LT
S linguale DSM 74               DHDHGP--PEQSARLGNARFYTYLR----EPFSYSAWKAAVAAGHTFVTSG--PILDLTAN-------------------------QALPGDRLALTKGEK------LT
Rhizobium sp. AC100             SV---------DSRPGIGRTYVKLPQRPVDDAGYSAWVDGLENGHLYWGDGRTHFLHFTVNGRESGKQP-------LAFVRASNVRVSATIAAMLELTPSPE-----TVQ
P fluorescens                   SV---------DSRPGIGRTYVKLPQRPVDDAGYSAWVDGLENGHLYWGDGRTHFLHFTVNGRESGKQP-------LAFVRASNVRVSATIAAMLELTPSPE-----TVQ
Poribacteria bacterium WGA-3G    -----------GERVGLGRIYVKMP---DGPLDFDAWVAGLKDGRSYVGDGKSHLLDFTVGGVPVGEEIATGEISQLNLEAPSTVTVTARVAARLG--ETPNLEIK-NRS
S acidiphila DSM 18658          -----------DERVGLGRVYVKLA---DGKLDFDRWVEGIKLGRSYVSDGKSHLVDFRVDDVAVGEAG-----SERPLAKPGTVKVSAQVAAYLEPSLTPALEALRNKP
A bacterium KBS 96              -----------DDRVGRGRSYVKVD----GPLTYASWLSGLKAGRSYVSDGRAHLMDFTVNGVEIGTHG-----SEAPLSSPGVAHATVRVAALLDSTPHPE---IQSLP
C flavus                        -----------GERVGLGRSYVKLP----PKWSYEDWCEGIRAGRNYVSDGFSHLLDFRVNGAQMGEEG-----SELRLPAPAQVHVTARVAGHLNPAPNEE---IRQAP
S paludicola KE                 -----------GDKVGLGRVYVKLPL--DQPVDYDTWVQGVKDGRSYCGDGLSHLFDFAVNGFEAGMTGPSGKSSQLDLKEPGKVRITCEAAALLAEKPTPQSDAIRAKR
Pseudomonas sp C5pp CH          -----------DFPGAERVYVDVG----GSPSPSAWFDGLRSRRSFITNW--LSADYSVN-------------------------GDKKATEYHIRAGDE------IT
Clustal Consensus                              *  * *.              *       :                                              .

                                        780       790       800       810       820       830       840       850       860       870       880
                                ....|....|....|....|....|....|....|....|....|....|....|....|....|....|....|....|....|....|....|....|....|
H rubra DSM19751                ISATARVNPARDSLTELELVVHGDVVASERSAE---GKEALHLQHSLK--LDRSLWFAIRTVGKHGTIAHTSAYYVTVDGTQRTWKQESVEDIAARFIQELKALGESSPD
A algicola                      IKAKALGNKDQIPLSKLEIIAHGKVLKEITPNGDVQSLENMELNFELE--VEKGTWIAARCFAGPNQVAHTTPIYITVNDG-GFHNPETFNDYLSLNEKYLDELETELEQ
C Solibacter                    IAAEALAPAT---VGTVEIVGHGKVLAKASGR----------VALSLP--VDHGMWIAARCDAGNGMVAHTTPVYVTVNGG-GFENRANYRENLAKMEGYLKELEGELAS
T bouteillei VB521301           ISVQAFGQEKQVPLERIELVGHGKVLASVTKNQPGQSASQLILNLELDT-LTRGIWLAARCFGGPQQAAHTTPIYVTVDGS-GFHNPETADRYLKLSERYLREIEQELEK
R lutea                         IRAEALGEATQEPLTALELVAHGRVLARVTPQQSGQSTARLVVQLELPD-VQQGLWIAARCYGGPRSAAHTTPVYVSVDGG-GFHNPETAQAYLTKSEAYLRELEAELDT
F limi BUZ 3                    VTVHAYGHADQVPLSALEVVSHGNVIGRITPTDAGQSAAHLSLDVSLES-IQRGQWIAARCYAGPGQAAHTTPIYVTVDGG-SFHNPETAPRYLDLCEKYLTEIEQALTG
S linguale DSM 74               IKAEAFGHAQQTPLNALELVAHGKVIGRVTKHDPGQSAGHLTITLNLPA-LTHGLWVAARCYGEGKQAAHTTPVYISLDGG-GFHNPETIGSYLTQSEQYLLELEKELDT
Rhizobium sp. AC100             NASEEPWHIEHARIGNSRKVAVELIVNGVAVKRQEMLADGSLQQISFKEKIDRSSWIAMRVLG----SGHTYPVFVTIGNKPIRASRRSAEWLRTCVDALWNEKHSLIRD
P fluorescens                   NASEEPWHIEHARIGNSRKVAVELIVNGVAVKRQEMLADGSLQQISFKEKIDRSSWIAMRVLG----SGHTYPVFVTIGNKPIRASRRSAEWLRTCVDALWNEKHSLIRD
Poribacteria bacterium WGA-3G    LDQQPYWDVERARIAESQKVPVELIVNGQTVETQEIVADGKVVPVQFEASIDKSSWVALRIFP----SSHTNPVFVIVDGKPIRASRRSAQWCLDAVEVCWSQKVNRIRE
S acidiphila DSM 18658          LDVQPYWDVERARIGAGRTVPVEVIVNGLPVARTEIVADGTFRDVAFEIAIDRSSWVALRIYP----TSHTNPVFVPVDGKPIRASKKSAEWCLKSVDQCWSKKERAIKP
A bacterium KBS 96              YDKEPYWTIERARIGDSREVPVEFIVNGAVAARKQVIADGAVHDVSFDVPISRSSWVAVRILP----AAHTNPIFVTVAGEPIRASRASAEWCRNAVHQCWTQKSPRIAV
C flavus                        VGQKPYWNIERARIADSQTVPVEVIVNGQSLGTQRIAANGTLQDVVFDVPIEGSSWVALRILP----SSHTNPIFVLVDGQPIRASKRSAEWCRKCVDQCWSQKERTYAA
S paludicola KE                 LDEKPYWHLERARIGDTQIVPVELIVNGVPVERKTIPADGSLQNLTFDYDLKQSSWIALRIMP----SCHTNPVFVEVAGKPVRASRSSADWCLKAVDVCWNAKQRAIRE
Pseudomonas sp C5pp CH          VDAVIRANPDFDAIDRVELVGHGEVLKLVSGTS---HASELRLLHKFK--PSGSMWLAVRAYGKGRALLHTSPIYVYVDGNKDFSDRQKLAGLADKYRKTLAEFRNSTPR
Clustal Consensus                            :   . :    ::                    :      . *.* *         ** . :: : .     .  .

                                        890       900       910       920
                                ....|....|....|....|....|....|....|....|....|..
H rubra DSM19751                VHADFESFDTADVMARQWDNAKPILKEQTERAIQTFQALARESRAR-
A algicola                      PNDRVD---------MRAWWYRDPLQKRIAETRKIIETLRKK-----
C Solibacter                    PGTSVD---------SQTFRHKAQLQREIAEARGKLRELGGSAGLGN
T bouteillei VB521301           ESDKPE---------FQLWHYKKGLKTRIAETREVIEGLKKRHEK--
R lutea                         HRDNPE---------FRAWYYKQPLKTRIEETRQVIADLRKRLVKP-
F limi BUZ 3                    PRPETE---------LQAWKYRDGLQTRIVEVRRILDDLRKKYKTP-
S linguale DSM 74               HSDNPE---------FRVWYYKKGLKTRIEETREVIRVLRRK-----
Rhizobium sp. AC100             TE-------------------LADAAAAYDHARQTYDRIIAESDLT-
P fluorescens                   TE-------------------LADAAAAYDHARQTYDRIIAESDLT-
Poribacteria bacterium WGA-3G   DE-------------------RDAAREAYDIASQTYQKILGESDID-
S acidiphila DSM 18658          NE-------------------RDAAAAAYDKARNAYKTILAEAVED-
A bacterium KBS 96              DQ-------------------LGAARTAYDRAEEVYKKLEAECSR--
C flavus                        AE-------------------LPDARAAYDHAREVYDRLITECTAN-
S paludicola KE                 -------------------RTSAKEAYNKAREVYAKVLGEATGE-
Pseudomonas sp C5pp CH          LEDEGERFAVDDLILARWNVVKPQLDKAIDDAIAIYDRLAAH-----
Clustal Consensus                                              .      :
```

Figure S5: Amino acid alignment of carbaryl hydrolase and its homologs. The boxed region represents the proposed conserved motif for the new family of esterase. The region highlighted in grey (447-594) of CH from strain C5pp represents phosphohydrolase type domain (PHP) as predicted by HMM library and genome assignment server[51].

The protein included for alignment are : *Haliea rubra* DSM 19751 [hypothetical protein HRUBRA_00780 KGE04621], *Arenibacter algicola* [hypothetical protein, partial WP_031445335], *Candidatus Solibacter usitatus* [hypothetical protein WP_011686006], *Tolypothrix bouteillei* VB521301 [hypothetical protein DA73_95705 KGG57257], *Rudanella lutea* [hypothetical protein WP_019987978], *Fibrisoma limi* [hypothetical protein WP_009284322], *Spirosoma linguale* [hypothetical protein WP_012927934], *Rhizobium* sp. AC100 [Carbaryl hydrolase WP_032490241, YP_009077292], *Pseudomonas fluorescens* [putative carbamate hydrolase CBY85381], *Poribacteria bacterium* WGA-3G [hypothetical protein WP_027451923], *Singulisphaera acidiphila* [hypothetical protein WP_015249644], *Acidobacteriaceae bacterium* KBS 96 [hypothetical protein WP_026336787], *Chthoniobacter flavus* [hypothetical protein WP_006978103], *Schlesneria paludicola* [hypothetical protein WP_010588133], Carbaryl hydrolase from *Pseudomonas* sp. strain C5pp [AMK37578].

(A)

```
                                 10        20        30        40        50        60        70        80        90
                         ....|....|....|....|....|....|....|....|....|....|....|....DHY..|....|....|....|....|....|
12DHNDO (AMK37580)     1 MAQIVAGFMLPHDPLIAAMPDAPPQDK--RDNCMNAYAKIADRLRELC--VDTVIVIGDDHYT-----IHGPACIPSCLIGI--GDVEGP 79
EDO (ACT53261)         1 MAKIVGGFMMPHDPLIPATPTAPPAAQ--REICMHAYAIIVERLRALQ--VDTVVVIADDHYT-----LNGPYCIPMAMIGI--GDIEGP 79
PhnC (AAD09870)        1 MAKIVGGFMMPHDPLIPATPTAPPAAQ--REICMHAYAIIVERLRALQ--VDTVVVIADDHYT-----LNGPYCIPMAMIGI--GDIEGP 79
CarBb (AAY56341)       1 ----MGKIVAAGGTSHILMSPKGCEES--AARVVNGIAELGRRLKEAR--PDVLVIITSDHMFNINLSMQPRFVVGIADSYTPMGDMDIP 82
LigB (AAA17728)        1 MARVTTGITSSHIPALGAAIQTGTSDNDYWGPVFKGYQPIRDWIKQPGNMPDVVILVYNDHASAFDMNIIPTFAIGCAETFKPADEGWGP 90
DHPADO (CAH38155)      1 MGKLSLAAKITHVPSMYLSELPGRHHG-CRAEAIRGHQAIGERCRALG--VDTIVVADTHWLVNAGYHVNCNGHFAGVYTSNELPHFIRD 87
ADO-a (AAB71525)       1 -MTIVSAFLVPGSPLPQLRADVPGWHN-----FQQAMRQAGAALKESR--PDVVLIYSTQWFAVLDEIWLTRRHSEDIHVDENWHEFGEL 82
MpcI (CAA36665)        1 --MPIQLECLSHTPLHGYV--DPAPEV--VAEVERVQAAARDRVRAFD--PELVVVFAPDHFNGFFYDVMPPFCIGAAATAI--GDFKS- 79
DHPPDO (AAB81314)      1 --MKQALLCMSHSPLLHHL--DPPADV--KASVEAAFDQARAFVHNFD--PDVIVNFGPDHYNGFFYDLMPPFCIGYKAKGS--GDYDS- 79
EDO (CAA06875)         1 --MTLALVCTSHSPLLEFN--NPPPEV--RTEVDRAFAQARQFIEEYD--PDLVVSFAPDHYNGFFYKLMPSFCIGFEASGV--GDFGS- 79
Clustal Consensus      1           .  .                              .      : ::    .                         .    3

                                100       110       120       130       140       150       160       170       180
                         ....|....|....|....|....|....|....|....|...DHG...|....|....|....|....|....|....|....|....|
12DHNDO (AMK37580)    80 WEEWLGIKRDVVINNEPLAHHIMSYGFDNGVDWAVS--KAMILDHSTSIPIHYAVRPAGGVR--AIPVYINSGCEPLISSRRAYEIGKVI 165
EDO (ACT53261)        80 YEPWLGIPRAQIENNRPLAHHIMQYGLEYGIDWAVS--KSLVLDHSATVPIHYAVRPVKGMR--AIPVYLNTGIEPFITSWRAHEIGRVI 165
PhnC (AAD09870)       80 YEPWLGIPRAQIENNRPLAHHIMQYGLEYGIDWAVS--KSLVLDHSATVPIHYAVRPVKGMR--AIPVYLNTGIEPFITSWRAHEIGRVI 165
CarBb (AAY56341)      82 --------RDLVPGSREVGRAIALQADEDGFDLCQA--EEYSLDHGIMIPILFMG--MKEIP--VVPVIVNINTDPIPSARRCVALAESI 158
LigB (AAA17728)       91 R------PVPDVKGHPDLAWHIAQSLILDEFDMTIM--NQMDVDHGCTVPLSMIFGEPEEWPCKVIPFPVNVVTYPPPSGKRCFALGDSI 172
DHPADO (CAH38155)     87 -------MRYEYPGNPALGHLIAASANERGIGTRAHEIDSLELEYGTLVPMRYMNADQRFKV------VSIAGWCMWHALDESRRFGEAL 164
ADO-a (AAB71525)      83 P--------YSLFSDVDLANACVEACRSQGVPARGADYKAYPIDTGTIVACTAMGIGTEALP------LVVASNNLYYNGEATENIGAIA 158
MpcI (CAA36665)       79 -------LAGKLPVPADLALSLAESVMAADIDVALS--HRMQVDHGCADALAALTGSLHRYP--VIPVFINSVAPPMATLRRARLLGDAV 158
DHPPDO (AAB81314)     79 -------FAGELNVPEAMAEDLAQFVMDQGLDIAIS--RQMEVDHGAVQPMEIIYGDVASKP--LIPVFVNSVARPFVKVARVRKFGEAV 158
EDO (CAA06875)        79 -------SAGRLDVPSALAEQMAQSVLDQGVDIAVS--LEMEVDHGAVQPLEILLGGIATKP--VIPIFVNSVAAPFAPMKRIRLLGEAV 158
Clustal Consensus      3                  :.          .           :: .   .                                   :.    7

                                190       200       210       220       230       240       250       260       270
                         ....|....|....|....|GXSH ....|....|....|....|....|....|....|....|....|....|....|....|....|
12DHNDO (AMK37580)   166 SEAVACWK-GNERVAIFGTGGLSHWPGMAEMG-------------------------------------------KVNPEWDHQVMELVK 211
EDO (ACT53261)       166 GDAVANWQ-GDERVAIYGTGGLSHWPGMAEMG-------------------------------------------AINEGWDRKIMKLVA 211
PhnC (AAD09870)      166 GDAVANWQ-GDERVAIYGTGGLSHWPGMAEMG-------------------------------------------AINEGWDRKIMKLVA 211
CarBb (AAY56341)     159 RQAIEKRTPDGCRVAVVGAGGLSHWLSVPRHG-------------------------------------------EVSEKFDHMVMDELV 205
LigB (AAA17728)      173 RAAVESFP-EDLNVHVWGTGGMSHQLQGPRAG-------------------------------------------LINKEFDLNFIDKLI 218
DHPADO (CAH38155)    165 RHAIDAS---DANVAFLASGSLSHRFNDNGSPEEAIHMISR----------------------------------EFYRQVDLRVVELWK 217
ADO-a (AAB71525)     159 AKAALDQ---GKRVAVIGVGGLSGSVFTSQIDPLDDHIVK-----------------------------------PEEDEWNRQILSLLE 210
MpcI (CAA36665)      159 GRFLSRA---GKRVLVVGSGGISHEPPVPELAGASEEVAERLIAGRNPSPES-AARQARTVAAAKSFVAGDSHLHPLNPEWDRAFLSLLA 244
DHPPDO (AAB81314)    159 GAYFKNS---DKKVLFIGSGGLSHDPPVPQIATADEAQRKMLTDGRNPTPQARAARQQRVIDTAVKFAADEADIMDLNPEWDRGFLDVCA 245
EDO (CAA06875)       159 GTFLKNF---DGNVLLLGSGGLSDPPVPRLEAATGEQRKMLLGCNHPTAAARAVRQQRVIDTAKAFTRGEAGIMDLAPEWDRQLLDILA 245
Clustal Consensus      7           . .* . . *.:*                                                          :  .:.    13

                                280       290       300       310       320       330       340       350
```

```
                                  ....|....|....|....|....|....|....|....|....|....|....|....|....|....|....|....|....|....
12DHNDO (AMK37580)           212 RGDVEALVFMSDEEILNNAGNGGLEIKNWICAMGALA----GWKGEIIAYEAVTEWVCGCGYMEMKPS--------------------- 275
EDO (ACT53261)               212 QGDVESLIALSDEEILRDGGNGGIEIKNWICAMGALG----ACRGEVIAYEPVAEWVCGCGYMEMKVA--------------------- 275
PhnC (AAD09870)              212 QGDVESLIALSDEEILRDGGNGGIEIKNWICAMGALG----ACRGEVIAYEPVAEWVCGCGYMEMKVA--------------------- 275
CarBb (AAY56341)             206 RGNAEKLVAMGNEAIIDQGGNAGVEILTWIMAAVASE----ASSGEKVFYEAMTQWFTGIGGMEFHVK--------------------- 269
LigB (AAA17728)              219 S-DPEELSKMPHIQYLRESGSEGVELVMWLIMRGALP----EKVRDLYTFYHIPASNTALGAMILQPEETAGTPLEPRKVMSGHSLAQA 302
DHPADO (CAH38155)            218 QGDFATFCKMLPEYNAHCHGEGGMHDTAMLLGLLGWD-------RYDKPVEIVTDYFASSGTGQINAIFPLP----------------- 282
ADO-a (AAB71525)             211 SGNLPALKEQIPHYVKAARVDMGFKHFNWVLGAIG------GEFRSAKVYHYGAIYGSGAAVVEFNL---------------------- 271
MpcI (CAA36665)              245 SGELTAVDGMTNDAITRDGGKSAHEIRTWVAAFGALA-AYGPYRASLDFYRAIPEWIAGFATMHAEPAAV------------------- 313
DHPPDO (AAB81314)            246 SGRIEDFDRYTADDMDAVAGHSSHEVRNWVAAYSALR-ACGEYEIAYEFYRPIKEYISGFAVTTAILRDI------------------- 314
EDO (CAA06875)               246 SGELERLDAWTAKEMAATAGNSAHEVRTWVAAHNALKAAAGQYTVTSEYYRPIPEYIAGFAVTTAVPVHKTAGLR-------------- 320
Clustal Consensus            13        .               . .    :    .                       . .                             14
```

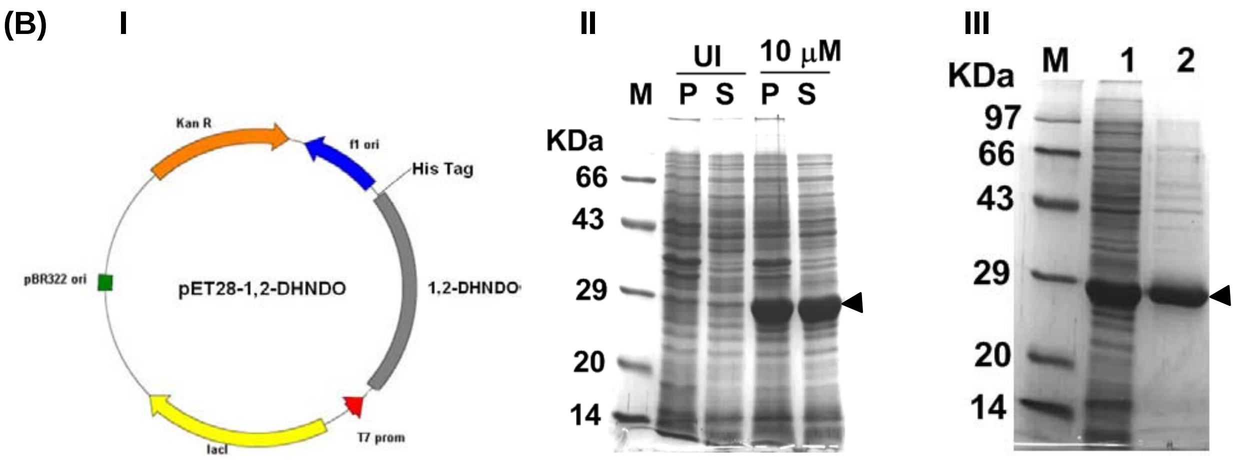

Fig. S6: (A) The alignment of 12DHNDO from *Pseudomonas* sp. strain_strain C5pp with other extradiol dioxygenase (EDO) reveals the presence of the conserved DHG and GXSH motifs which may be involved in the formation of the iron coordination sphere (grey boxed region). The numbers in parentheses indicates the protein accession id. Enzyme abbreviations: 12DHNDO, 1,2-dihydroxynaphthalene dioxygenase, *Pseudomonas* sp. strain C5pp; EDO, extradiol dioxygenase, *Burkholderia* sp. C3; PhnC, extradiol dioxygenase, *Burkholderia sartisoli*; CarBb, *Pseudomonas* sp. XLDN4-9; LigB, protocatechuate 4,5-dioxygenase-α subunit, *Sphingomonas paucimobilis*; DHPADO, 3,4-dihydroxyphenylacetate 2,3-dioxygenase, *Burkholderia pseudomallei* K96243; ADO-a, 2-aminophenol-1,6-dioxygenase alpha subunit, *Pseudomonas pseudoalcaligenes* ; MpcI, dioxygenase, *Cupriavidus necator*; DHPPDO, 2,3-dihydroxyphenylpropionate 1,2-dioxygenase, *Rhodococcus globerulus*; EDO, extradiol dioxygenase, *Rhodococcus* sp. I1. (**B-I**) A map of 12DHNDO construct, pET28-12DHNDO. (**B-II**) SDS-PAGE analysis of 12DHNDO expression from uninduced 'UI' and induced 'I' *E. coli* BL21(DE3) harboring pET28a-12DHNDO. Induction was carried out using 10 μM IPTG at 30°C for 4 h. 'P' indicates pellet or insoluble protein fraction, 'S' denotes supernatant or soluble protein fraction and 'M' indicates protein mol. wt. markers. Solid arrow head indicates the 12DHNDO protein (~30 kDa). (**B-III**) Partial purification of 12DHNDO using one-step NiNTA affinity chromatography, lane 1; CFE, lane 2; partially purified 12DHNDO.

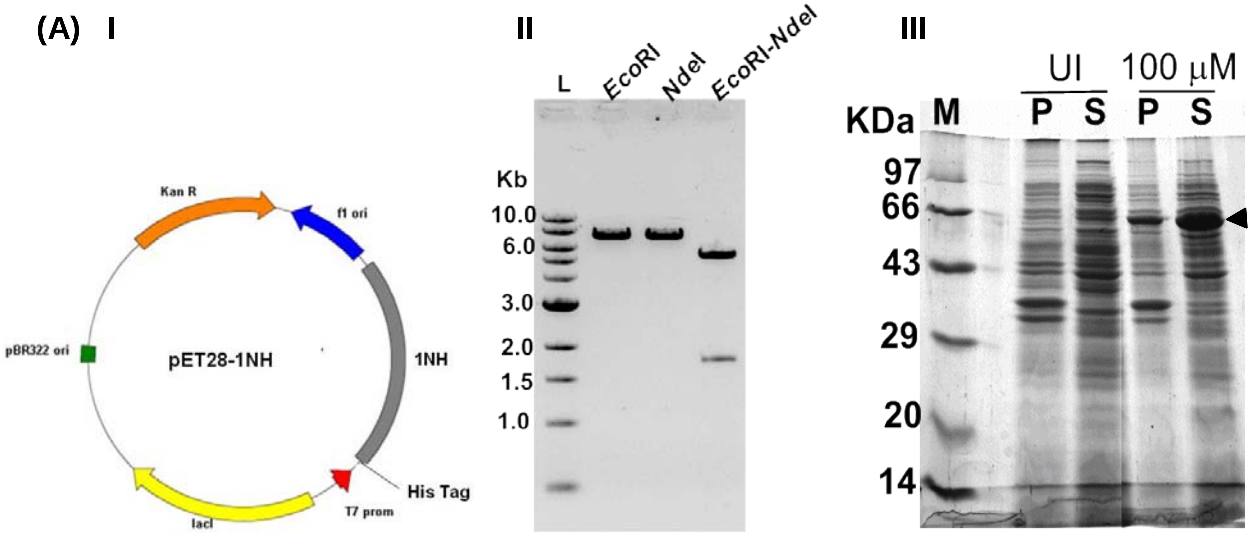
(A) I
Kan R
f1 ori
pBR322 ori
pET28-1NH
1NH
His Tag
T7 prom
lacI
II
L
EcoRI
NdeI
EcoRI-NdeI
Kb
10.0
6.0
3.0
2.0
1.5
1.0
III
UI
100 μM
KDa
M
P
S
P
S
97
66
43
29
20
14

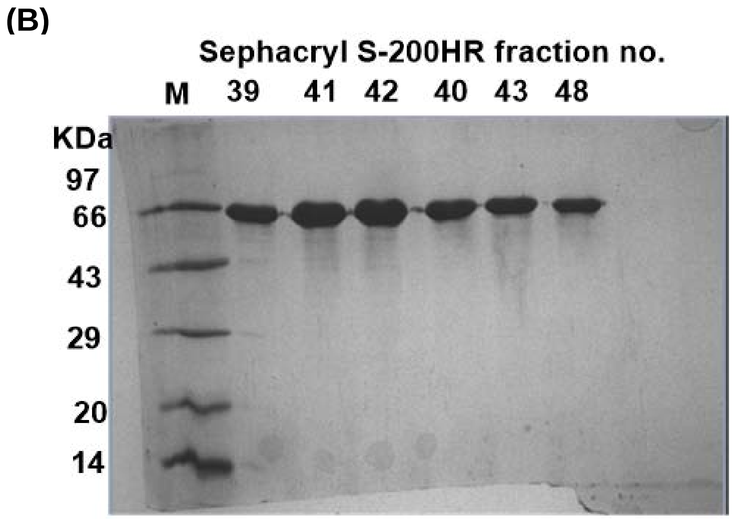
(B)
Sephacryl S-200HR fraction no.
M
39
41
42
40
43
48
KDa
97
66
43
29
20
14

(C)

```
                ....|....| ....|....| ....|....| ....|....| ....|....| ....|....| ....|....| ....|....|
                        10         20         30         40         50         60         70         80
AMK37581.1      MLKNIFLKDE IRKVSVPVFI VGAGPSGLTT AALLAKYGVK VLAVSQHSGT ANSPRAHITN TRAMEVFRDL GIEEKVYDVA
wt1NH           ---------- --KVSVPVFI VGAGPSGLTT AALLAK---- VLAVSQHSGT ANSPRAHITN TRAMEVFRDL GIEEKVYDVA
r1NH            ---------- ---------- ---------- ---------- VLAVSQHSGT ANSPRAHITN TRAMEVFRDL GIEEKVYDVA
Clustal Co                                                  ********** ********** ********** **********

                ....|....| ....|....| ....|....| ....|....| ....|....| ....|....| ....|....| ....|....|
                        90        100        110        120        130        140        150        160
AMK37581.1      SVEEMENVVW CTRLAGQELA RLSSWGYGDD RRSEYLTASP SKMCNAAQHV LEPVLLDVAR ESGAEVLFNT EVLEIEQSSE
wt1NH           SVEEMENVVW CTRLAGQELA RLSSWGYGDD RRSEYLTASP SKMCNAAQHV LEPVLLDVAR ESGAEVLFNT EVLEIEQSSE
r1NH            SVEEMENVVW CTRLAGQELA RLSSWGYGDD RRRSEYLTAP SKMCNAAQHV LEPVLLDVAR ESGAEVLFNT EVLEIEQSSE
Clustal Co      ********** ********** ********** ********** ********** ********** ********** **********

                ....|....| ....|....| ....|....| ....|....| ....|....| ....|....| ....|....| ....|....|
                       170        180        190        200        210        220        230        240
AMK37581.1      KVYALVRNRE TGEEYIVESD YAVGADGARS LVARSLGFEF EGKMGEGHAV NCWFDADLSK YCADRKAVLF PTLQPGNDDW
wt1NH           KVYALVRNRE TGEEYIVESD YAVGADGARS LVARSLGFEF EG-------- ---------K YCADR----- ----------
r1NH            KVYALVRNRE TGEEYIVESD YAVGADGAR  ---------- ---------- ---------- ---------- ----------
Clustal Co      ********** *********  *********

                ....|....| ....|....| ....|....| ....|....| ....|....| ....|....| ....|....| ....|....|
                       250        260        270        280        290        300        310        320
AMK37581.1      IGGGTFICVK PWNDWILLFM YNPDNADAET NEEAVLERIY TLIGDRDVDV NIKAISKWTV NNMFAKSMVK ERIVLMGDAA
wt1NH           ---------- ---------- ---------- -------KIY TLIGDRDVDV NIKAISKWTV NNMFAKS--- -RIVLMGDAA
r1NH            ---------- ---------- ---------- --------IY TLIGDRDVDV NIKAISKWTV NNMFAK--- ---IVLMGDAA
Clustal Co                                               ** ********** ********** ******       ********

                ....|....| ....|....| ....|....| ....|....| ....|....| ....|....| ....|....| ....|....|
                       330        340        350        360        370        380        390        400
AMK37581.1      HRHPPAGGLG TNTCVQDAFN LSWKLAYIIN GYAGQDLLQS YSDERQPVAK QMVNRAITAM VNMQAVPQAL GFFPGQSEED
wt1NH           HRHPPAGGLG TNTCVQDAFN LSWKLAYIIN GYAGQDLLQS YSDERQPVAK ---------- ---------- ----------
r1NH            HRHPPAGGLG TNTCVQDAFN LSWKLAYIIN GYAGQDLLQS YSDERQPVAK --------- ---------- ----------
Clustal Co      ********** ********** ********** ********** **********

                ....|....| ....|....| ....|....| ....|....| ....|....| ....|....| ....|....| ....|....|
                       410        420        430        440        450        460        470        480
AMK37581.1      GWDALEVFSS PTEEGKTRRK KMREALELQN WQFNGIGGEA GHHYLSSHAI VSDGTTAEFN SENSQVHIHP TTTPGATLPH
wt1NH           ---------- ---------- ---------- ---------- ---------- ---------- ---------- ----------
r1NH            ---------- ---------- ---------- ---------- ---------- ---------- ---------- ----------
Clustal Co                                                                                        ****

                ....|....| ....|....| ....|....| ....|....| ....|....| ....|....| ....|....| ....|....|
                       490        500        510        520        530        540        550        560
AMK37581.1      AWLQRNGVNV SSIDLVGNGK FTIITGLGGD AWLRSAAKVS ESLGIDVNLV SISVDGDITD HYGAWADVSE IEDDGCLLVR
wt1NH           ---QRNGVNV SSIDLVGNGK FTIITGLGGD AWLRS----- ---------- ---------- ---------- ----------
r1NH            -----NGVNV SSIDLVGNGK FTIITGLGGD AWL------ ---------- ---------- ---------- ----------
Clustal Co           ***** ********** ********** ***

                ....|....| ....|....| ....|....|
                       570        580        590
AMK37581.1      PDRFVAYRSK GLVSDTDAAI SQAFRAILCL
wt1NH           -------RSK GLVSDTDAAI SQAFRA----
r1NH            -------RSK GLVSDTDAAI SQAFR-----
Clustal Co             *** ********** *****
```

Figure S7: Functional analysis of 1-naphthol 2-hydroxylase (1NH).
(**A-I**) A map of 1NH construct, pET28a-1NH and (**A-II**) the release of 1.8 kb insert after restriction digestion with *Nde*I and *Eco*RI. (**A-III**) SDS-PAGE analysis of 1NH expression from uninduced 'UI' and induced 'I' *E. coli* BL21(DE3) harboring pET28a-1NH. Induction was carried out using 100 µM IPTG at 18 °C for 16 h. 'P' indicates pellet or insoluble protein fraction, 'S' denotes supernatant or soluble protein fraction and 'M' indicates protein mol. wt. markers. Solid arrow head indicates the 1NH protein (~66 kDa). (**B**) SDS-PAGE analysis of r1NH fractions eluted from Sephacryl S200-HR. (**C**) Amino acid alignment of translated 1-naphthol 2-hydroxylase (1NH) from database (AMK37581.1) with the peptide sequences

obtained after MALDI-TOF/TOF analysis of 1NH purified from *Pseudomonas* sp. strain C5pp (wt1NH) and recombinant 1NH (r1NH) cloned, expressed and purified from *E. coli*. The ClustalW consensus is shown as star. The sequence highlighted in grey represents N-terminus and peptide sequence obtained from purified wt1NH.

(A)

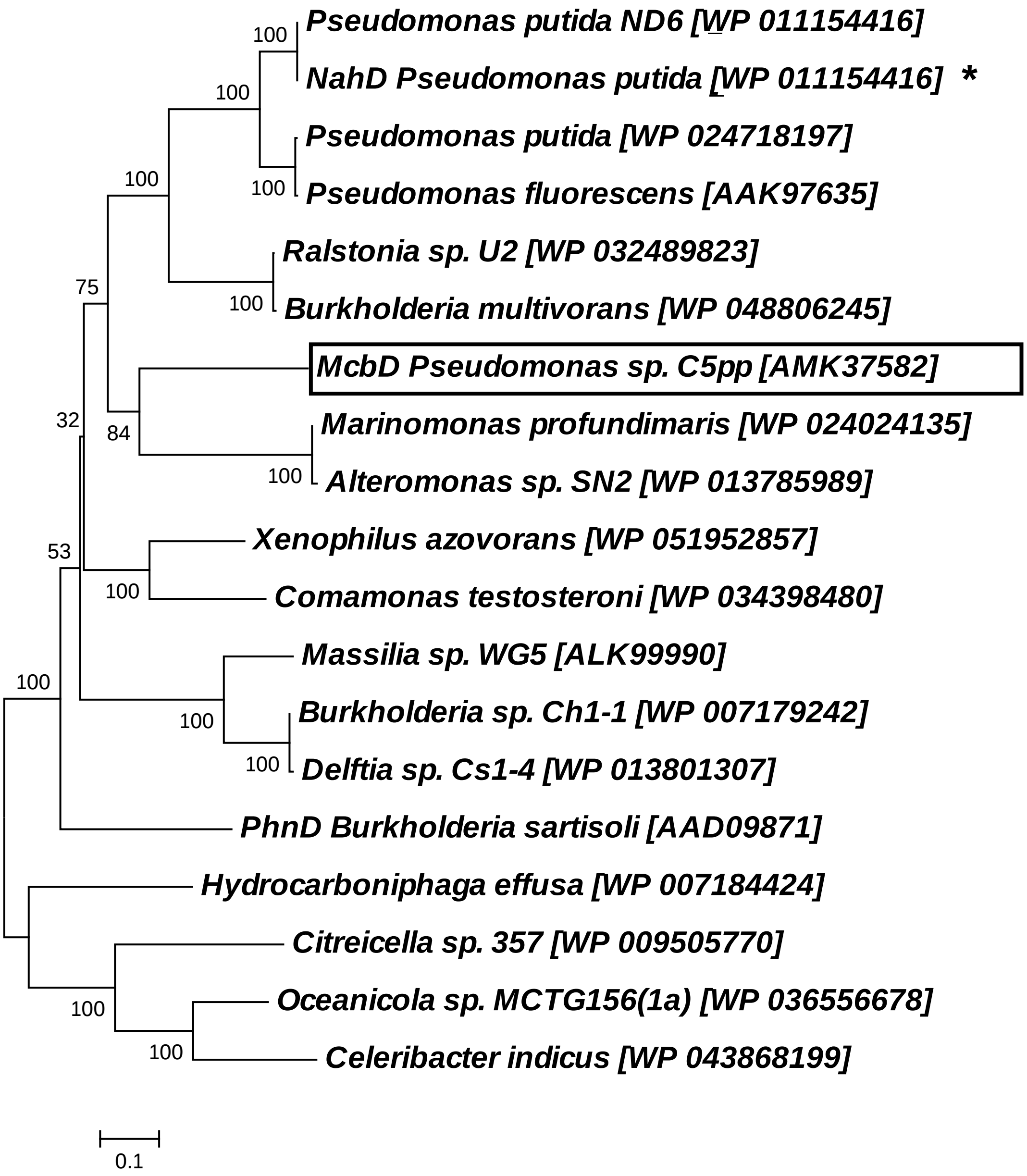
Pseudomonas putida ND6 [WP 011154416]
NahD Pseudomonas putida [WP 011154416] *
Pseudomonas putida [WP 024718197]
Pseudomonas fluorescens [AAK97635]
Ralstonia sp. U2 [WP 032489823]
Burkholderia multivorans [WP 048806245]
McbD Pseudomonas sp. C5pp [AMK37582]
Marinomonas profundimaris [WP 024024135]
Alteromonas sp. SN2 [WP 013785989]
Xenophilus azovorans [WP 051952857]
Comamonas testosteroni [WP 034398480]
Massilia sp. WG5 [ALK99990]
Burkholderia sp. Ch1-1 [WP 007179242]
Delftia sp. Cs1-4 [WP 013801307]
PhnD Burkholderia sartisoli [AAD09871]
Hydrocarboniphaga effusa [WP 007184424]
Citreicella sp. 357 [WP 009505770]
Oceanicola sp. MCTG156(1a) [WP 036556678]
Celeribacter indicus [WP 043868199]
100
100
100
100
75
100
32
84
100
53
100
100
100
100
100
100
0.1

(B)

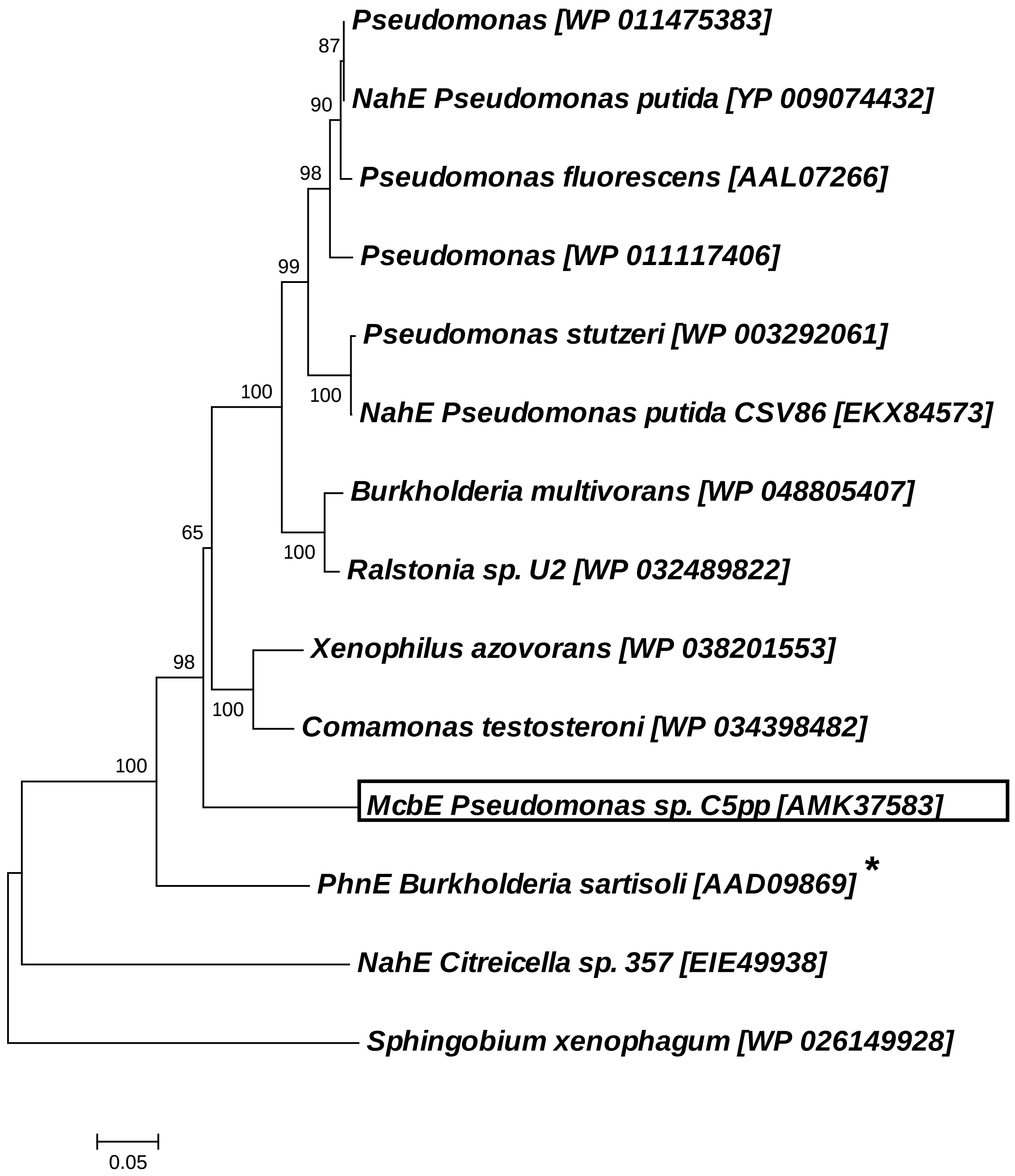
Pseudomonas [WP 011475383]
87
NahE Pseudomonas putida [YP 009074432]
90
Pseudomonas fluorescens [AAL07266]
98
Pseudomonas [WP 011117406]
99
Pseudomonas stutzeri [WP 003292061]
100
100
NahE Pseudomonas putida CSV86 [EKX84573]
Burkholderia multivorans [WP 048805407]
65
100
Ralstonia sp. U2 [WP 032489822]
Xenophilus azovorans [WP 038201553]
98
100
Comamonas testosteroni [WP 034398482]
100
McbE Pseudomonas sp. C5pp [AMK37583]
PhnE Burkholderia sartisoli [AAD09869] *
NahE Citreicella sp. 357 [EIE49938]
Sphingobium xenophagum [WP 026149928]
0.05

(C)

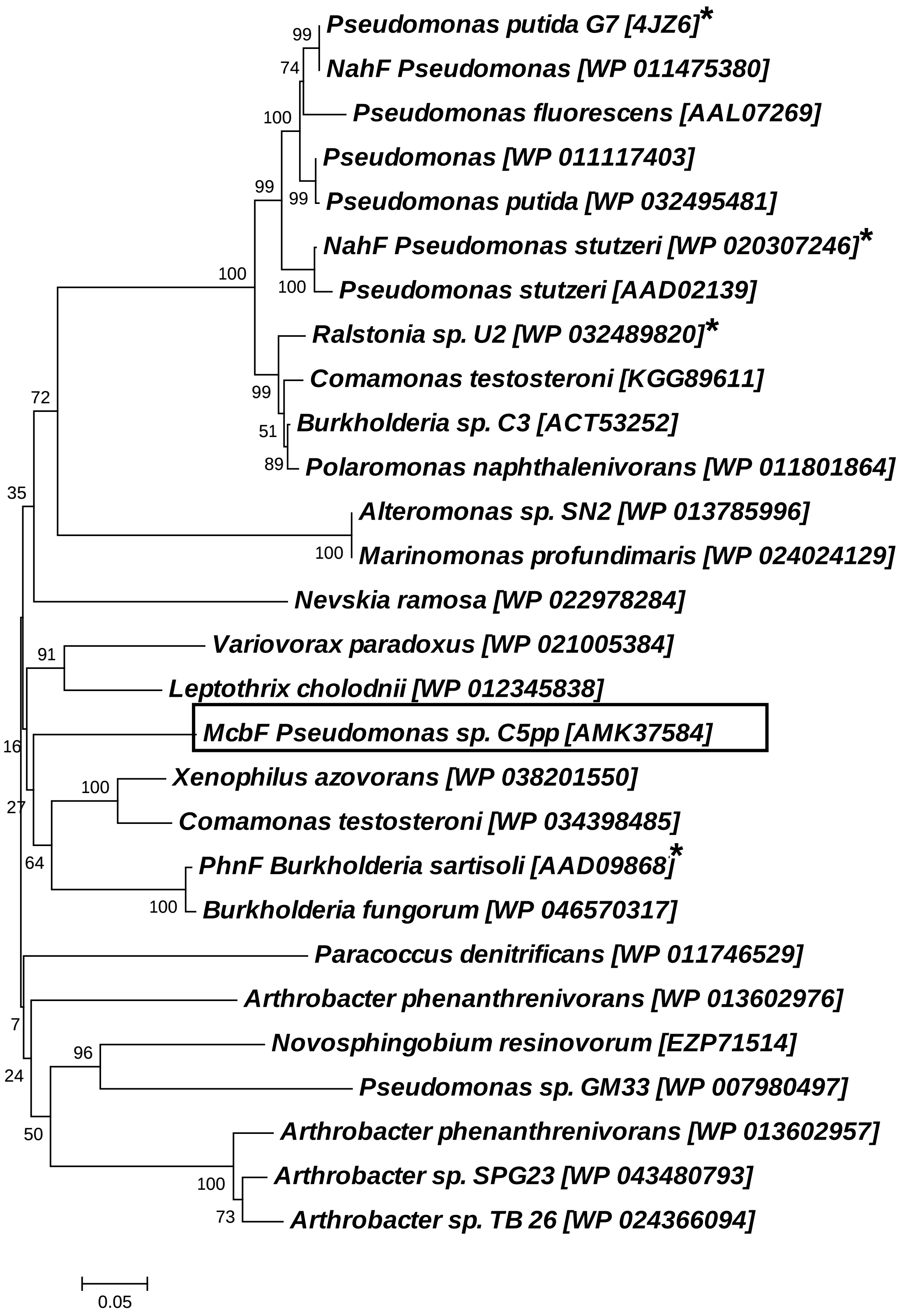
Pseudomonas putida G7 [4JZ6]*
NahF Pseudomonas [WP 011475380]
Pseudomonas fluorescens [AAL07269]
Pseudomonas [WP 011117403]
Pseudomonas putida [WP 032495481]
NahF Pseudomonas stutzeri [WP 020307246]*
Pseudomonas stutzeri [AAD02139]
Ralstonia sp. U2 [WP 032489820]*
Comamonas testosteroni [KGG89611]
Burkholderia sp. C3 [ACT53252]
Polaromonas naphthalenivorans [WP 011801864]
Alteromonas sp. SN2 [WP 013785996]
Marinomonas profundimaris [WP 024024129]
Nevskia ramosa [WP 022978284]
Variovorax paradoxus [WP 021005384]
Leptothrix cholodnii [WP 012345838]
McbF Pseudomonas sp. C5pp [AMK37584]
Xenophilus azovorans [WP 038201550]
Comamonas testosteroni [WP 034398485]
PhnF Burkholderia sartisoli [AAD09868]*
Burkholderia fungorum [WP 046570317]
Paracoccus denitrificans [WP 011746529]
Arthrobacter phenanthrenivorans [WP 013602976]
Novosphingobium resinovorum [EZP71514]
Pseudomonas sp. GM33 [WP 007980497]
Arthrobacter phenanthrenivorans [WP 013602957]
Arthrobacter sp. SPG23 [WP 043480793]
Arthrobacter sp. TB 26 [WP 024366094]
99
74
100
99
99
100
100
99
51
89
72
35
100
91
16
27
100
64
100
7
96
24
50
100
73
0.05

(D)

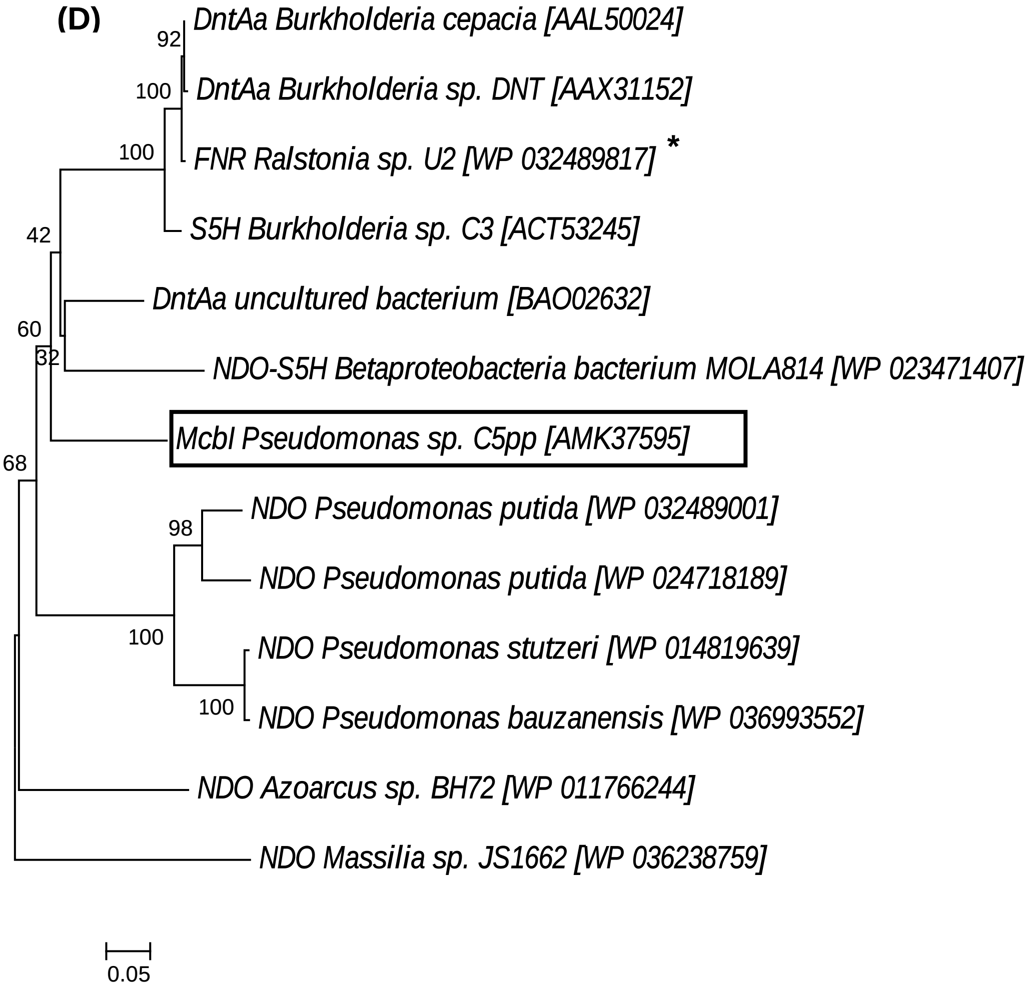
DntAa Burkholderia cepacia [AAL50024]
92
100
DntAa Burkholderia sp. DNT [AAX31152]
100
FNR Ralstonia sp. U2 [WP 032489817] *
S5H Burkholderia sp. C3 [ACT53245]
42
DntAa uncultured bacterium [BAO02632]
60
32
NDO-S5H Betaproteobacteria bacterium MOLA814 [WP 023471407]
McbI Pseudomonas sp. C5pp [AMK37595]
68
NDO Pseudomonas putida [WP 032489001]
98
NDO Pseudomonas putida [WP 024718189]
100
NDO Pseudomonas stutzeri [WP 014819639]
100
NDO Pseudomonas bauzanensis [WP 036993552]
NDO Azoarcus sp. BH72 [WP 011766244]
NDO Massilia sp. JS1662 [WP 036238759]
0.05

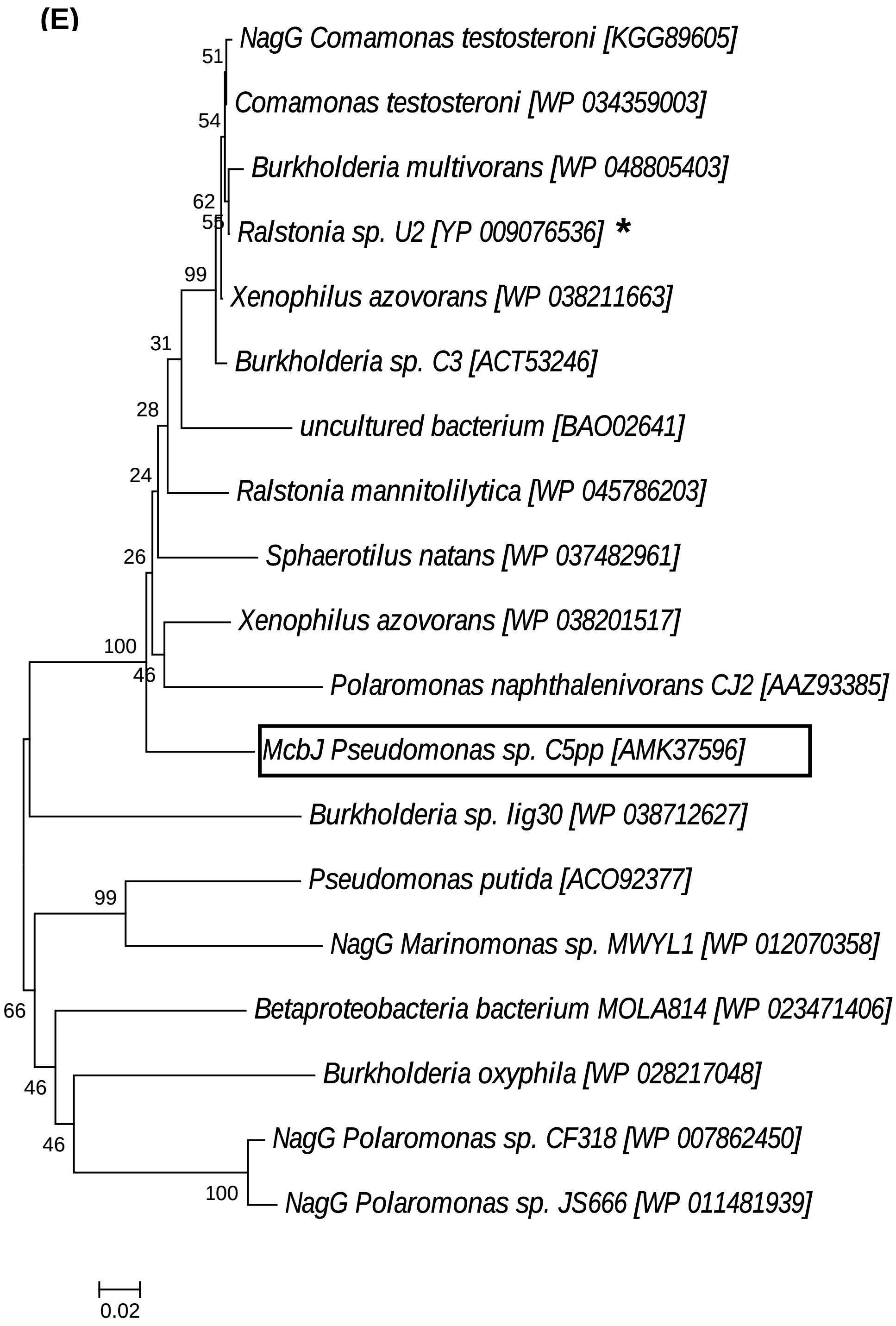
(E)
NagG Comamonas testosteroni [KGG89605]
Comamonas testosteroni [WP 034359003]
Burkholderia multivorans [WP 048805403]
Ralstonia sp. U2 [YP 009076536] *
Xenophilus azovorans [WP 038211663]
Burkholderia sp. C3 [ACT53246]
uncultured bacterium [BAO02641]
Ralstonia mannitolilytica [WP 045786203]
Sphaerotilus natans [WP 037482961]
Xenophilus azovorans [WP 038201517]
Polaromonas naphthalenivorans CJ2 [AAZ93385]
McbJ Pseudomonas sp. C5pp [AMK37596]
Burkholderia sp. lig30 [WP 038712627]
Pseudomonas putida [ACO92377]
NagG Marinomonas sp. MWYL1 [WP 012070358]
Betaproteobacteria bacterium MOLA814 [WP 023471406]
Burkholderia oxyphila [WP 028217048]
NagG Polaromonas sp. CF318 [WP 007862450]
NagG Polaromonas sp. JS666 [WP 011481939]
51
54
62
55
99
31
28
24
26
100
46
99
66
46
46
100
0.02

(F)

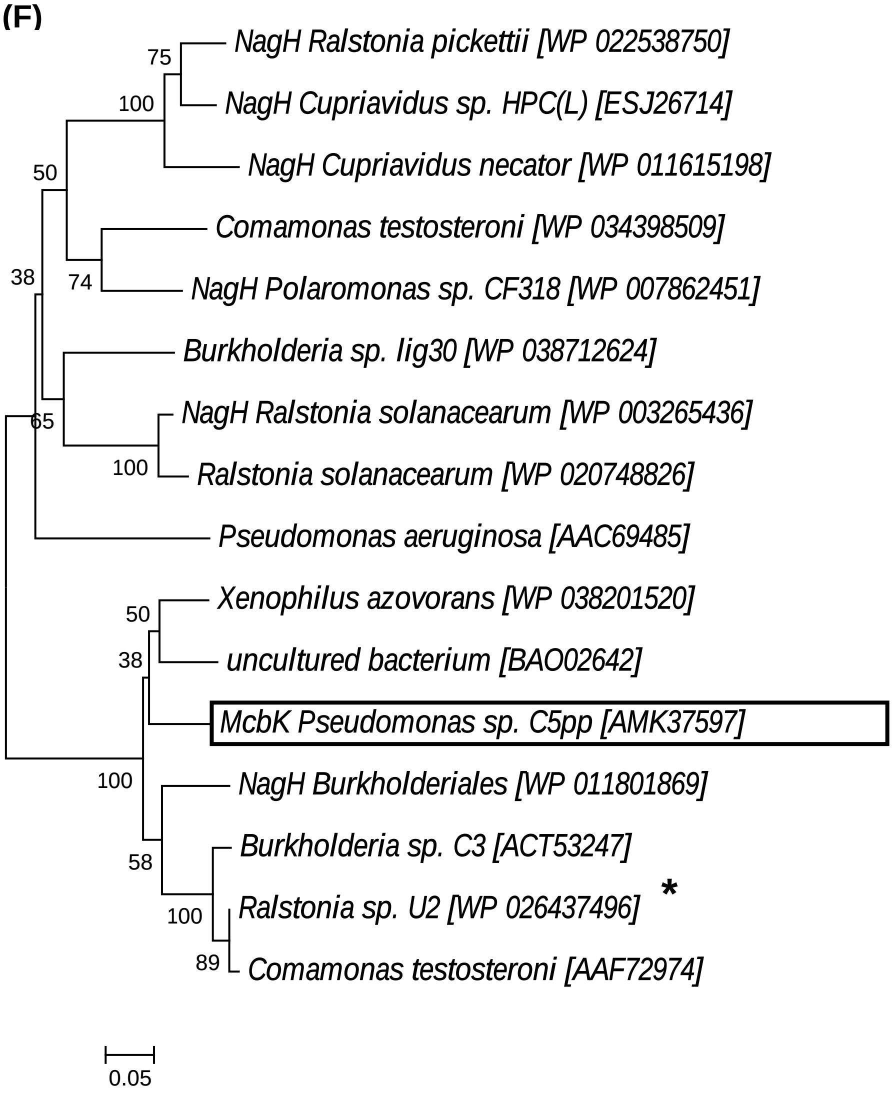
NagH Ralstonia pickettii [WP 022538750]
NagH Cupriavidus sp. HPC(L) [ESJ26714]
NagH Cupriavidus necator [WP 011615198]
Comamonas testosteroni [WP 034398509]
NagH Polaromonas sp. CF318 [WP 007862451]
Burkholderia sp. lig30 [WP 038712624]
NagH Ralstonia solanacearum [WP 003265436]
Ralstonia solanacearum [WP 020748826]
Pseudomonas aeruginosa [AAC69485]
Xenophilus azovorans [WP 038201520]
uncultured bacterium [BAO02642]
McbK Pseudomonas sp. C5pp [AMK37597]
NagH Burkholderiales [WP 011801869]
Burkholderia sp. C3 [ACT53247]
Ralstonia sp. U2 [WP 026437496]
*
Comamonas testosteroni [AAF72974]
75
100
50
38
74
65
100
50
38
100
58
100
89
0.05

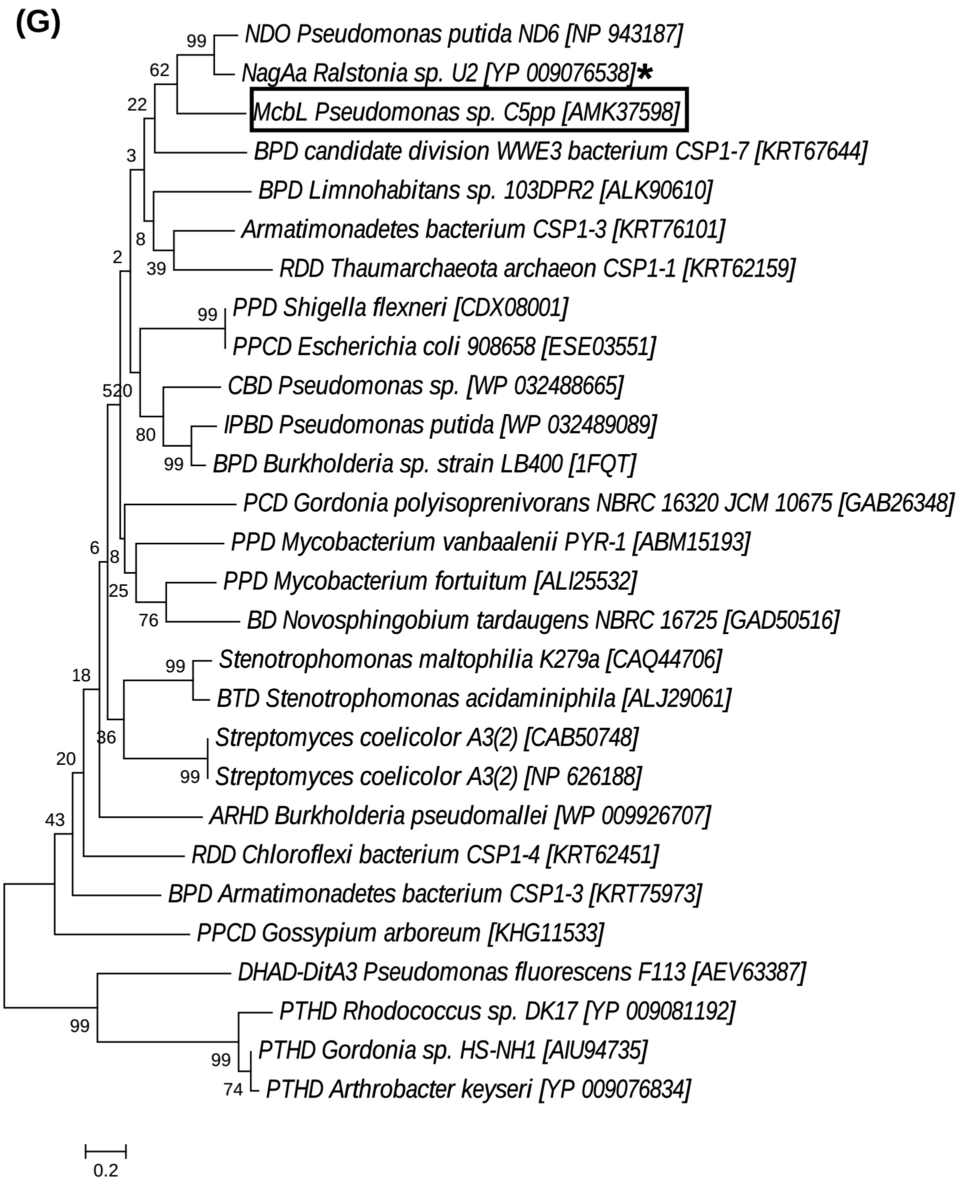

Fig. S8: Phylogenetic analysis of **(A)** 2-hydroxychromene-2-carboxylate isomerase; (**B**) *trans*-o-hydroxybenzylidenepyruvate hydratase-aldolase; (**C**) salicylaldehyde dehydrogenase and (**D**) Salicylate 5-hydroxyalse, reductase component; (**E**) Salicylate 5-hydroxyalse, oxygenase large subunit; (**F**) Salicylate 5-hydroxyalse, oxygenase small subunit and (**G**) Salicylate 5-hydroxyalse, ferredoxin-component from *Pseudomonas* sp. strain C5pp with their respective homologs. The sequences were retrieved from NCBI database and aligned using MEGA6 with Neighbour-joining algorithm. The numbers indicate accession ids. Asterisk (*) indicates that protein is functionally characterized.

DNtAa, dintrotoluene dioxygenase ferredoxin-reductase; FNR, ferredoxin NADPH-reductase; S5H, salicylate 5-hydroxylase; NDO, naphthalene dioxygenase; PPCD, 3-phenylpropionate/cinamic acid dioxygenase; PPD, phenylpropionate dioxygenase; PCD, p-cumarate dioxygenase; BPD, biphenyl dioxygenase; RDD, Rieske (2Fe-2S) domain dioxygenase; BD, benzene dioxygenase; BTD, benzene-toluene dioxygenase; PTHD, phthalate dioxygenase; ARHD, aromatic ring hydroxylating dioxygenase; CBD, chlorobenzene dioxygenase; IPBD, isopropylbenzne dioxygenase; NDO, naphthalene dioxygenase; NagAa, ferredoxin component of salicylate 5-hydroxylase; DHAD-DitA3, dehydroabietic acid dioxygenase

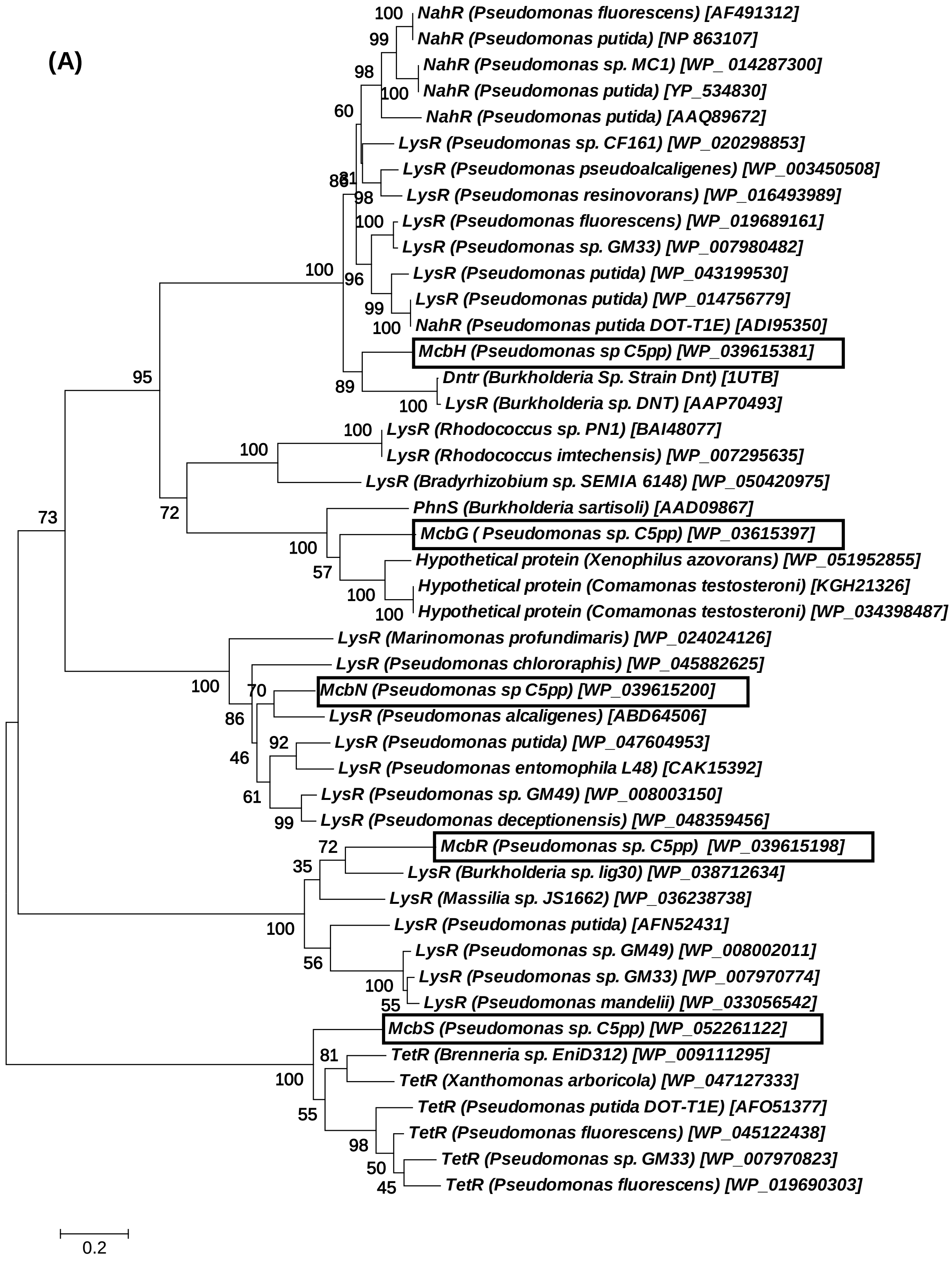
(A)
NahR (Pseudomonas fluorescens) [AF491312]
NahR (Pseudomonas putida) [NP 863107]
NahR (Pseudomonas sp. MC1) [WP_ 014287300]
NahR (Pseudomonas putida) [YP_534830]
NahR (Pseudomonas putida) [AAQ89672]
LysR (Pseudomonas sp. CF161) [WP_020298853]
LysR (Pseudomonas pseudoalcaligenes) [WP_003450508]
LysR (Pseudomonas resinovorans) [WP_016493989]
LysR (Pseudomonas fluorescens) [WP_019689161]
LysR (Pseudomonas sp. GM33) [WP_007980482]
LysR (Pseudomonas putida) [WP_043199530]
LysR (Pseudomonas putida) [WP_014756779]
NahR (Pseudomonas putida DOT-T1E) [ADI95350]
McbH (Pseudomonas sp C5pp) [WP_039615381]
Dntr (Burkholderia Sp. Strain Dnt) [1UTB]
LysR (Burkholderia sp. DNT) [AAP70493]
LysR (Rhodococcus sp. PN1) [BAI48077]
LysR (Rhodococcus imtechensis) [WP_007295635]
LysR (Bradyrhizobium sp. SEMIA 6148) [WP_050420975]
PhnS (Burkholderia sartisoli) [AAD09867]
McbG (Pseudomonas sp. C5pp) [WP_03615397]
Hypothetical protein (Xenophilus azovorans) [WP_051952855]
Hypothetical protein (Comamonas testosteroni) [KGH21326]
Hypothetical protein (Comamonas testosteroni) [WP_034398487]
LysR (Marinomonas profundimaris) [WP_024024126]
LysR (Pseudomonas chlororaphis) [WP_045882625]
McbN (Pseudomonas sp C5pp) [WP_039615200]
LysR (Pseudomonas alcaligenes) [ABD64506]
LysR (Pseudomonas putida) [WP_047604953]
LysR (Pseudomonas entomophila L48) [CAK15392]
LysR (Pseudomonas sp. GM49) [WP_008003150]
LysR (Pseudomonas deceptionensis) [WP_048359456]
McbR (Pseudomonas sp. C5pp) [WP_039615198]
LysR (Burkholderia sp. lig30) [WP_038712634]
LysR (Massilia sp. JS1662) [WP_036238738]
LysR (Pseudomonas putida) [AFN52431]
LysR (Pseudomonas sp. GM49) [WP_008002011]
LysR (Pseudomonas sp. GM33) [WP_007970774]
LysR (Pseudomonas mandelii) [WP_033056542]
McbS (Pseudomonas sp. C5pp) [WP_052261122]
TetR (Brenneria sp. EniD312) [WP_009111295]
TetR (Xanthomonas arboricola) [WP_047127333]
TetR (Pseudomonas putida DOT-T1E) [AFO51377]
TetR (Pseudomonas fluorescens) [WP_045122438]
TetR (Pseudomonas sp. GM33) [WP_007970823]
TetR (Pseudomonas fluorescens) [WP_019690303]
0.2

(B)

```
                                             10        20        30        40        50        60        70        80        90       100
                                     ....|....|....|....|....|....|....|....|....|....|....|....|....|....|....|....|....|....|....|....|
McbG [Pseudomonas sp. C5pp]          ------MGYKNRSLEISTLRGINMNLLPILAELLRMESVTKASERLHLTQSTVSGSLKQLRGIFKDDLLVQRGRGMVLTEKAKRLKPEVDRMIELVE-KL 93
PhnS [Burkholderia sartisoli]        ----------MSNQVVRNLRSVNMNLLPVLGELLRCPNVSHAANRLHLTQSTVSGSLKQLRLLFEDDLLVQRGREMVLTEKAKELRPAVERLLEQAS-RL 89

McbH [Pseudomonas sp. C5pp]          ----------------MELRDIDLNLLVIFHQLLVDRKVSTAAEKLGLTQPAVSNALKRLRTLLQDELFVRTSRMMEPTPYAQQLAEPIAYALSTLQGAL 84
NahR [Pseudomonas putida]            ----------------MELRDLDLNLLVVFNQLLVDRRVSITAENLGLTQPAVSNALKRLRTSLQDPLFVRTHQGMEPTPYAAHLAEPVTSAMHALRNAL 84

McbN[Pseudomonas sp. C5pp]           ---------------MDWSRRLRLRHLHLLVSLAETGSLSDTARLAHTTQPGLSKWLKELEEDVGATLFERHARGLRPTAHGQLLLGHARRILSEMARAQ 85
HbzR [Pseudomonas alcaligenes]       MICSHAPIRIKIYAMFDWTRRLRLRHLHLLVSLAETGNMSDTARLAHTTQPGLSKWLKELEEDVGVPLFERHARGLKPTVHGQTLLGHARRILSEMARAQ 100
Clustal Consensus                                       * : :. * :: .*     :: ::.    **. :*  **.*.  .   *: :  : :  *  .  *       :        26

                                            110       120       130       140       150       160       170       180       190       200
                                     ....|....|....|....|....|....|....|....|....|....|....|....|....|....|....|....|....|....|....|....|
McbG [Pseudomonas sp. C5pp]          FEVDHFDPETATTNFQIATADYVSALVTKNLGVYLEAHSPNISLT-LKPTPGTSAKELQIGSLDLIICPDLPTNWESCGLKKGDE--RFSHQVFMHDKMV 190
PhnS [Burkholderia sartisoli]        FCAETFDPATAANCFRIATADYVSALVTSRLGPVLQANAPGVSIT-LTPTPGTTAKDLRLGVVDLIICPNRRENWEACGIAAHDP--EFCHEVFIRDRLV 186

McbH [Pseudomonas sp. C5pp]          NQQVTFEPKTSNRNFTLAMTDIGEIYFMPKLMDTLADLAPNIKISTVRNSEISLSDEMQSGAVDLAIG----------LLPSLQA--GFFQRRLFHHRYV 172
NahR [Pseudomonas putida]            QHHESFDPLTSERTFTLAMTDIGEIYFMPRLMDVLAHQAPNCVISTVRDSSMSLMQALQNGTVDLAVG----------LLPNLQT--GFFQRRLLQNHYV 172

McbN[Pseudomonas sp. C5pp]           HNLEALQ-EGDSRSVALGTSPASAPSLVPDAITYFLRQHPRSRVQLQESTMNALLERLEQGELDVVVG----------RLDSYQPRATLRSELLYHEPMQ 174
HbzR [Pseudomonas alcaligenes]       HNLEMLH-EGESRSVALGTSPAAAPSLVPDAITYFLRRYPKARVQLQESTMSTLLDRLEQGQLDVVIG----------RLDNYQPRASLCSERLHSEPMR 189
Clustal Consensus                         :.       . :. :      .       :    *   :     :  :  . :. * :*: :            :   :    :  . :  .    44

                                            210       220       230       240       250       260       270       280       290       300
                                     ....|....|....|....|....|....|....|....|....|....|....|....|....|....|....|....|....|....|....|....|
McbG [Pseudomonas sp. C5pp]          AIQCGRHRAAGRP-IELIDYLTRPHAIYCRTDGKETIEQEALSRLGLKQH-IQISVPYFTLLPQLLEGTDLISIVPESIARLYGDIHSIDVFPPPMNFLH 288
PhnS [Burkholderia sartisoli]        AIQSANRSLAERP-LALADYLARPHAMYCRTDGQPTIEQETLAQMGLVQR-IQFLVPYFTLLPQLVVDSHLVALIPLSLANHYARLFPLDVFEPPVPFPS 284

McbH [Pseudomonas sp. C5pp]          CMYRKDHPIAGQS-LTAEKFCELEHVRVISANTGHGEVEKYLARAGIKRS-VRLEVPHFVAVGYILQKTDLIATVPERFAACCVSPFGLSFSPAPLNLPE 270
NahR [Pseudomonas putida]            CLCRKDHPVTREP-LTLERFCSYGHVRVIAAGTGHGEVDTYMTRVGIRRD-IRLEVPHFAAVGHILQRTDLLATVPIRLADCCVEPFGLSALPHPVVLPE 270

McbN[Pseudomonas sp. C5pp]           VVARPGHPLSGRRELGWEDLLAYDWILWPEGTPIRSRLDMALTQAGLKPLPCRVESSSLMGNLWLLQNSDMISVASGRVAEHFTARGLVVSLDFELETQG 274
HbzR [Pseudomonas alcaligenes]       VVARPGHPLSSRDGLGWEELYAHDWLIWPEGTPIRSKLDNALANAGQPPLPYRVESSSLTANLWLLQNSDMLSVASGSVAKHFIARGLLAPLDFELDAEG 289
Clustal Consensus                     :    :  : .  :                       :  ::. *      :.  . :     ::  :.:::  .  .*        :      :     63

                                            310       320       330       340       350       360       370       380       390       400
                                     ....|....|....|....|....|....|....|....|....|....|....|....|....|....|....|....|....|....|....|....|
McbG [Pseudomonas sp. C5pp]          ST-------------------------------------------------------------------------------------------------- 290
PhnS [Burkholderia sartisoli]        LDLVMIGACHRADRADLYWLRKIVRDSASSFLDIGNEGGCLETASACAPTDVDVTTAGTPLLPPRALARPGHGENRYRVSEESVSPSSSALISFPQ---- 380

McbH [Pseudomonas sp. C5pp]          IAINLFWHAKYHRDP------ANRWLRQLIFDLFAD---------------------------------------------------------------- 300
NahR [Pseudomonas putida]            IAINMFWHAKVPQGPSQYLVAGNRCLTCLRIEKKSEPINVMGSDLIQPACALAGNGGSSSTGSSRRRCCAAHQPSRRRRSARPKMTAPSVELFWFFAGSQ 370

McbN[Pseudomonas sp. C5pp]           SIGMCWRDEPLMDGSVLDLLDCLRQAANTPIRHLQPTPPIA----------------------------------------------------------- 315
HbzR [Pseudomonas alcaligenes]       SIGMCWRDEPLQDSSTQELLDCLRQASRVPIRELRTVAALE----------------------------------------------------------- 330
Clustal Consensus                                                                                                                         63
```

```
                                  ....
McbG [Pseudomonas sp. C5pp]       ---- 290
PhnS [Burkholderia sartisoli]     ---- 380

McbH [Pseudomonas sp. C5pp]       ---- 300
NahR [Pseudomonas putida]         DREN 374

McbN[Pseudomonas sp. C5pp]        ---- 315
HbzR [Pseudomonas alcaligenes]    ---- 330
Clustal Consensus                      63
```

Fig. S9: **(A)** The phylogenetic analysis of regulatory proteins (McbG, McbH, McbN, McbR and McbS) present in the Supercontig-A in *Pseudomonas* sp. strain C5pp. The numbers in parentheses indicate the protein accession id. Amino acid alignment of transcriptional regulators proposed to be involved in carbaryl degradation (*mcb*G, *mcb*H and *mcb*N) with PhnS (AAD09867), NahR (YP_534830) and HbzR (ABD64506)-LysR type transcriptional regulators. **(B)** Amino acid alignment of transcriptional regulators proposed to be involved in carbaryl degradation (*mcb*G, *mcb*H and *mcb*N) with PhnS (AAD09867), NahR (YP_534830) and HbzR (ABD64506)-LysR type transcriptional regulators. The amino acid residues identical in all the sequences are shaded in black. The grey shaded region represents helix-turn-helix (HTH-type) domain involved in DNA binding as predicted by InterPro (http://www.ebi.ac.uk/interpro/). The region in box depicts coinducer recognition domain. The shaded nucleotides in the box represent the changes observed in the strain C5pp sequence.

Table S1: Sequence analysis of G1 sub-clones

Sub-clone size (~)		Sequence obtained (nt) / % coverage	Most similar gene product	Amino acid identity (%) / Query coverage
12 kb	M13FP	1194 / 10	TetR family transcriptional regulator (*Pseudomonas* WP_007910277.1)	66/63
	M13 RP	1032 / 8.6	Putative LysR transcriptional regulator (*Pseudomonas alcaligenes* NCIMB 9867 ABD 64506.1)	81/83
8 kb	M13 FP	1199 / 15	Putative transposase (*Pseudomonas veronii* AF173167.3)	99/77
	M13 RP	1245 / 15.5	2,4-dichlorophenol 6-monooxygeanse (*Cupriavidus basilensis*)	58/77
5 kb	M13 FP	1205 / 21	Putative transposase (*Pseudomonas veronii* AF173167.3)	99/75
	M13 RP	1165 / 19	Hypothetical protein (*Pseudomonas aeruginosa* BWH051 WZP06642.1)	100/16
2.8 kb	M13 FP	1225 / 43.7	Reverse transcriptase (*Pseudomonas putida* WP013970361.1)	98/26
	M13 RP	1202 / 43	Hypothetical protein (*Pseudomonas aeruginosa* WZP023100449.1)	82/57
2.5 kb	M13 FP	1209 / 48.3	Putative LysR transcriptional regulator (*Pseudomonas alcaligenes* NCIMB 9867 ABD 64506.1)	25/75
	M13 RP	1182 / 47.3	Ser/Thr protein phosphatase (*Pseudomonas stutzeri* WP014821613.1)	83/73
1.8 kb	M13 FP	1140 / 63	Integrase (*Pesudomonas pelagia*)	91/96
	M13 RP	1155 / 64	ISPsy14 transposase (*Pseudomonas syringae* pv actinidae ICMP 19096 EPN_26475.1)	97/88

Table S2: Primers used for primer walking and gap filling reactions for assembling contigs involved in carbaryl degradation

Region to be amplified	Primers		Expected size (bp)
	Name	5’ Sequence 3’	
Contig47-contig76	F1	ATTGGGGATTGGTGCAAGCAGC	~250
	R1	GACCCGACCTTGGGCGATGCG	
Contig76-Contig61	F2	ACGCATGGATATACGCTCCACG	~250
	R2	TTGAGACGGCGGGCGACGGTACTGACG	
Contig61-Contig62	F3-1	CATGGTCCCCAGCAATGTGGTCCGG	
	R3-1A	AAGTGACGATCCTGCGACAGTTTGCCG	
	F3-1C	AAGGCTTCCAATACAGCTGG	
	R3-1C	TGCGGTAATCGATGTCTTCC	
Contig62-Contig92	F6	AATTGAAGCGCGAAGGAAAGC	~650
	R6	AAGACTGTGAAGAGCGGACAACC	
Contig92-Contig68	F5	TTGTCGATATCCTCCACTTCC	~850
	R5	CTTTCCTTCGCGCTTCAATTCG	
Contig68-Contig83	F4	CATCCGATAAGGAGCAACGAGTTTAGG	~750
	R4	TGTTCATCCTGGACTGGCTGC	

Table S3: Analysis of genes present on Supercontig-A involved in carbaryl degradation

**Start....End** (direction of transcription)	**Start....End**	**Gene / Protein**	**Size (bp)**	**Protein Homolog**	**Coverage / Identity**	**E-value**
**Supercontig-A**	**Contig 83**					
(-) 334…1368	(-) 334…1368	Transposase	1035	IS110 transposase [*Pseudomonas putida*] (AJ288910)	100/93	0
(-) 1476...4442	1476...	Transposase	2967	Transpsoase [Gammaproteobacteria] (WP_001138014)	100/99	0
	**Contig 68**					
(-) 4446...5060	(+) 4692...5306	Transpsoase	615	TnpR [*Pseudomonas aeruginosa*] (ACY75537)	100/98	0
(-) 5381....6394	(+) 3358....4371	Integrase	1014	Integrase/recombinase [*E. coli* BIDMC 82] (EZQ51649)	100/100	0
(+) 6324....7331	(-) 2421...3428	Aminoglycoside nucleotidyl transferase	1008	Aminoglycoside nucleotidyl transferase ANT3 [*E. coli*] (WP_001297038)	100/100	0
(+) 7836....8675	(-) 1077....1916	Dihydropterate synthase	840	Dihydropterate synthase [*Acinetobacter baumannii* A424] (AFB76381)	100/100	0
(+) 8803....9303	(-) 449....949	N-acetyltransferase	501	GCN5-related N-acetyltransferase [*Desulfurispirillum indicum* S5] (ADU64769)	100/100	0
	**Contig 92**					
(-) 9735....10556	(-) 2....832	Transposase	822	MULTISPECIES: transposase [Gammaproteobacteria] (WP_000259708)	100/100	0
(+) 9780....10574	(+) 29....823	Transposase	795	MULTISPECIES: transposase [Gammaproteobacteria] (WP_001375121)	100/100	0

	**Contig 62**					
(-) 11174…13483	(-) 545....2854	Carbaryl hydrolase (CH)	2310	Hypothetical protein HRUBRA_00780 [*Haliea rubra* DSM 19751] (KGE04621)	92/40	0
(-) 13993....15066	(-) 3364.... 4437	MetA-pathway phenol degradation-like protein	1074	MetA-pathway phenol degradation-like protein [*Pseudomonas denitrificans*] (WP_015476950)	100/81	1e-176
(-) 15330....16157	(-) 4701....5528	1,2-Dihydroxynaphthalene dioxygenase (12DHNDO)	828	MULTISPECIES: protocatechuate 3,4-dioxygenase [Bacteria] (WP_025527768)	99/74	2e-150
(-) 16616....18388	(-) 5987....7759	1-Napthol 2-hydroxylase (1NH)	1773	2,4-Dichlorophenol 6-monooxygenase [*Burkholderia zhejiangensis*] (KDR27163)	97/55	0
(-) 18424....19017	(-) 7795....8388	2-Hydroxychromene 2-carboxylate isomerase	594	2-Hydroxychromene 2-carboxylate isomerase [*Marinomonas profundimaris*] (WP_024024135)	97/55	e-173
(-) 19114....20121	(-) 9077....9492	*trans*-o-hydroxybenzylidenepyruvate hydratase-aldolase	1008	Aldolase [*Xenophilus azovorans*] (WP_038201553)	98/79	0
(-) 20166....21638	(-) 9537....11009	Salicylaldehyde dehydrogenase (SalDH)	1473	Salicylaldehyde dehydrogenase [*Comamonas testosteroni*] (KGH21325.1)	100/77	0
(+) 21951....22082	(+) 11322....11453	Mobile element protein	132	Transposase [*Pseudomonas* sp. RL] (WP_027591894)	100/84	5e-15
(+) 22083....22232	(+) 11454....11603	Mobile element protein	150	Transposase IS3 [*Comamonas testosteroni*] (KGH23063)	100/92	5e-24
(+) 22254....22556	(+) 11625....11927	Mobile element protein	303	ISCja1 transposase *orf*B [*Pseudomonas putida* CSV86] (EKX86906)	99/86	5e-53\
(+) 22784....23656	(+) 12155....13027	LysR	873	LysR-type transcriptional regulator, PhnS [*Burkholderia sartisoli*] (AAD09867.1)	95/58	2e-158
(+) 23785....23883	(+) 13156....13254	Transposase	99	ISPssy transposase [*Pseudomonas syringae* pv. actinidiae] (KPZ20061)	100/91	1e-11

	**Contig 76**					
(-) 24407...25162	(-) 128....883	Mobile element protein	756	ISPre4_aa2; IS21 Family [*Pseudomonas resinovorans*] (BAH10054)	100/93	e-128
(-) 25183....26697	(-) 904....2418	Mobile element protein	1515	ISPre4_aa1; IS21 Family [*Pseudomonas resinovorans*] (WP_011077976)	100/90	0
	**Contig 61**					
	318....1	Molybdopterin subunit	318	Nicotinate dehydrogenase medium molybdopterin subunit [*Desulfosporosinus meridiei*] (WP_014902240)	88/31	9.4
(+) 26912...27726	(+)....804	Major Facilitator superfamily	747	MFS transporter [*Pseudomonas* sp. GM78] (WP_008055311)	95/70	2e-113
(+) 27794....28822	(+) 871....1899	Transcriptional regulator	1029	Transcriptional regulator [*Pseudomonas aeruginosa*] (WP_057380974)	100/99	0
(+) 29352....30254	(+) 2429....3154	Transcriptional regulator (NahR)	903	Naphthalene degradation LysR-family transcriptional activator [*Pseudomonas* sp. CF161] (WP_020298853)	100/74	7e-163
(+) 30637....31623	(+) 3714....4700	Naph 1,2-dioxygenase/sal 5-hydroxylase, ferredoxin--NAD(P)(+) reductase	987	Oxidoreductase component of 2,4-dinitrotoluene dioxygenase DntAa [uncultured bacterium] (BAO02623.1)	100/72	e-46
(+) 31694....32956	(+) 4771....6033	Salicylate 5-hydroxylase large oxygenase component	1263	Salicylate-5-hydroxylase large oxygenase component [*Burkholderia* sp. C3] (ACT53246)	100/90	0
(+) 32959....32444	(+) 6036....6521	Salicylate 5-hydroxylase small oxygenase component	486	Salicylate 5-hydroxylase small oxygenase component [*Xenophilus azovorans*] (WP_038201520)	100/87	2e-87
(+) 33453....33767	(+) 6530....6844	Naphthalene 1,2-dioxygenase/salicylate 5-	315	Naphthalene 1,2-dioxygenase [*Polaromonas naphthalenivorans*]	99/71	5e-49

		hydroxylase, ferredoxin component		(WP_011801868)		
(+) 34512....35873	(+) 7589....8950	4-Hydroxy benzoate transporter	1362	Major facilitator transporter [*Tistrella mobilis*]	95/56	e-162
(-) 36297...37322	(-) 9374...10399	Transposase	1026	IS1383_aa1; IS110 Family [*Pseudomonas putida*] (AAC98740)	100/83	e-160
(-) 37599....38129	(-) 10676....11206	Transposase	531	IS1384_aa1; IS5 Family [*Pseudomonas putida*] (AAC98743)	87/79	7e-67
(-) 38140....39393	(-) 11217....12470	Reverse transcriptase	1254	MULTISPECIES: group II intron reverse transcriptase/maturase [*Pseudomonas*] (WP_039615390)	99/99	0
(-) 40082....40978	(-) 13159....13938	Integrase	897	Integrase [*Pseudomonas pelagia*] (WP_022963153)	100/92	2e-175
	**Contig 76**					
(+) 41193….42707	(-) 904....2418	Mobile element protein	1515	ISPre4_aa1; IS21 Family [*Pseudomonas resinovorans*] (WP_011077976)	100/90	0
(+) 42728...43483	(-) 128....883	Mobile element protein	756	ISPre4_aa2; IS21 Family [*Pseudomonas resinovorans*] (BAH10054)	100/93	e-128
	**Contig 47**					
	(-) 411....1	MFS Transporter	411	MULTISPECIES: MFS transporter [*Pseudomonas*] (WP_019470817)	100/81	3e-71
(-) 44099....44896	(-) 489....1286	Protein involved in meta-pathway of phenol degradation	798	MULTISPECIES: phenol degradation protein [*Pseudomonas*]	100/77	4e-155
	(-) 1423....1827	Hypothetical protein	405	MULTISPECIES: hypothetical protein [*Pseudomonas*] (WP_033902220)	100/99	0
(+) 45279....46343	(+) 1669....2733	Transposase	1065	Protein containing transposase DDE	100/99	0

				domain [*Pseudomonas aeruginosa* PA7] (ABR81870)		
(+) 47243...47878	(+) 3368....3835	Transcriptional regulator (TetR)	636	TetR family transcriptional regulator [*Brenneria* sp. EniD312] (WP_009111295)	96/64	0
(+) 47951....49450	(+) 4341....5840	Fusaric acid resistance protein	1500	Fusaric acid resistance protein [*Brenneria* sp. EniD312] (WP_009111294)	97/99	0
(+) 49447....50667	(+) 5837....7057	Hemolysin	1221	Hemolysin D [*Brenneria* sp. EniD312] (WP_009111293)	100/84	0
	6987....7637	Unknown	651	Unknown		
(+) 50646....52196	(+) 7066....8586	Arabinose ABC transporter permease	1521	Arabinose ABC transporter permease [*Brenneria* sp. EniD312] (WP_009111292)	98/79	3e-165
(-) 52238....53164	(-) 9554....8628	Transcriptional regulator (LysR)	927	LysR family transcriptional regulator [*Burkholderia* sp. lig30] (WP_038712634)	99/68	3e-88
	8898....9353	Hypothetical protein	456	Unknown		
(+) 53278....54135	(+) 9668....10525	Pirin and Cupin2 superfamily related protein	858	Hypothetical protein [*Novosphingobium lindaniclasticum*] (WP_021235888)	98/60	4e-114
(+) 54259....54879	(+) 10649…11269	Hypothetical protein	621	Hypothetical protein [*Oceanicola* sp. MCTG156(1a)] (WP_036557598)	99/83	2e-123
(-) 55126…55767	(-) 11516....12157	Maleyl pyruvate isomerise	642	Maleylacetoacetate isomerase [*Pseudomonas chlororaphis*] (WP_025810436)	99/73	8e-107
(-) 55914....56612	(-) 12304....13002	Fumaryl pyruvate hydrolase	699	5-Carboxymethyl-2-hydroxymuconate isomerase [*Pseudomonas chlororaphis*] (WP_025810433)	100/81	2e-138
(-) 56627....57682	(-) 14072....13017	Gentisate dioxygenase	1056	Gentisate 1,2-dioxygenase [*Pseudomonas* sp. GM48] (WP_033063630)	99/81	0

(-) 57689....58093	(-) 14483....14079	Hypothetical protein	405	Hypothetical protein [*Actinomyces dentalis*] (WP_026409902)	36/49	0.27
(+) 57861....58811	(+) 14251....15201	Transcriptional regulator (LysR)	951	Putative Lys-R type transcriptional regulator [*Pseudomonas alcaligenes*] (ABD64506)	98/79	3e-165
(+) 58969....59739	(+) 15359....16129	Transposase	771	Transposase [*Pseudomonas stutzeri*] (WP_036998747)	99/90	7e-42
(+) 60029...60907	(+) 16419...17297	Ser/Thr protein phosphatase	879	Ser/Thr protein phosphatase [*Pseudomonas stutzeri*] (WP_014821613)	83/84	5e-149
(+) 62680....63459	(+) 19070....19849	Transposase	780	ISAeme21; IS481 Family [*Aeromonas media*] (AHX59485)	99/71	2e-128
	19160....19876	Hypothetical protein	717	Hypothetical protein		
(-) 63456....64481	(-) 20871....19846	Transposase	1026	Transposase [*Azotobacter vinelandii*] (WP_012699613)	100/93	5e-177
(+) 64269....64712	(+) 20659....21102	Integrase	444	Integrase [*Burkholderia phymatum*] (WP_012406582)	100/100	0
(+) 64582....66120	(+) 20972....22510	Transposase	1539	MULTISPECIES: transposase [*Pseudomonas*] (WP_039615185)	100/100	0
(+) 67787....68575	(+) 24177....24965	Inositol monophosphatase	789	Inositol monophosphatase [*Pseudomonas moraviensis*] (WP_024014393)	98/60	8e-103
(-) 70494....71057	(-) 27447....26884	Recombinase	564	Recombinase [*Pseudomonas* sp. GM78] (WP_008059144)	93/42	5e-59
(+) 71084....72577	(+) 27474....28967	Hypothetical protein	1494	Hypothetical protein [*Pseudomonas putida*] (WP_049275073)	63/53	3e-78
(-) 72574....73491	(-) 29881....28964	Hypothetical protein	918	Hypothetical protein [*Pseudomonas putida*] (WP_046817441)	93/42	5e-59
(+) 74005....74655	(+) 30395....31045	Hypothetical protein	651	TnsA endonuclease [*Pseudomonas* sp. LAIL14HWK12:I9] (WP_027615650)	87/34	4e-24
(+) 74659....76332	(+) 31049....32722	Hypothetical protein	1674	Integrase [*Pseudomonas* sp. GM78] (WP_008059139)	99/46	4e-149
